# Distinct *in vitro* utilization and degradation of porcine gastric mucin glycans by human intestinal bacteria

**DOI:** 10.1093/femsec/fiaf066

**Published:** 2025-06-23

**Authors:** Carol de Ram, Maryse D Berkhout, Carolina O Pandeirada, Jean-Paul Vincken, Guido J E J Hooiveld, Clara Belzer, Henk A Schols

**Affiliations:** Laboratory of Food Chemistry, Wageningen University & Research, 6700 AA Wageningen, the Netherlands; Laboratory of Microbiology, Wageningen University & Research, 6700 EH Wageningen, the Netherlands; Laboratory of Food Chemistry, Wageningen University & Research, 6700 AA Wageningen, the Netherlands; Laboratory of Food Chemistry, Wageningen University & Research, 6700 AA Wageningen, the Netherlands; Division of Human Nutrition and Health, Wageningen University & Research, 6700 EH Wageningen, the Netherlands; Laboratory of Microbiology, Wageningen University & Research, 6700 EH Wageningen, the Netherlands; Laboratory of Food Chemistry, Wageningen University & Research, 6700 AA Wageningen, the Netherlands

**Keywords:** mucin glycan degradation, human gut microbiota, ecological interactions, *Akkermansia muciniphila*, *Ruminococcus torques*, *Bacteroides thetaiotaomicron*

## Abstract

Mucin glycan degradation and utilization by microbes colonizing the human intestine is an essential host–microbe interaction. In this study, degradation and utilization of porcine gastric mucin glycans by *Akkermansia muciniphila, Ruminococcus torques, Bacteroides thetaiotaomicron*, co-cultures, and a synthetic bacterial community were investigated over time. Liquid chromatography–tandem mass spectrometry *O-*glycan patterns revealed that all three monocultures removed sialic acid residues. Furthermore, *R. torques* first targeted fucosylated *O-*glycans, while *A. muciniphila* and *B. thetaiotaomicron* equally favoured fucosylated and non-fucosylated *O*-glycans. *A. muciniphila, R. torques*, and *B. thetaiotaomicron* favoured degradation of first core 2 *O*-glycan structures relative to core 1 *O*-glycan structures. Co-cultures, compared to monocultures, demonstrated different *O-*glycan degradation patterns suggesting distinct ecological interactions between the bacteria. Although extensive *O-*glycan degradation was observed by the monocultures and co-cultures, only the synthetic community completely degraded all *O-*glycans within 24 h. Regarding degradation of the constituent *N-*glycans, matrix-assisted laser desorption ionization-time-of-flight mass spectrometry showed that *A. muciniphila* and *R. torques* can partly degrade *N-*glycans, *B. thetaiotaomicron* can completely degrade high-mannose *N-*glycans, and the synthetic community can degrade all *N-*glycans. The utilization of mucin glycans was observed by production of different metabolites among the bacteria. These results indicate that degradation of mucin glycans depends on microbial interactions and ecological networks.

## Teaser

The degradation and utilization of mucin glycans depend on microbial composition and the interactions between the present bacteria.

## Introduction

The human gastrointestinal tract (GIT) is covered by a single layer of epithelial cells, which are protected by a thick intestinal mucus layer. The mucus layer of the human GIT consists of an inner and an outer layer. The outer layer harbours mucosal bacteria that have a profound influence on intestinal health by degrading and utilizing mucins, the main functional components of the mucus layer (Koropatkin et al. 2012). Mucin degradation is an essential part of the normal turnover process necessary to maintain intestinal health (Van Herreweghen et al. 2018). Degradation of mucin glycans results in production of metabolites, including short-chain fatty acids (SCFAs) that have positive effects on host energy metabolism (Den Besten et al. 2013). Furthermore, specific mucin glycan-degrading bacteria, such as *Akkermansia muciniphila*, are associated with beneficial properties, including stimulation of mucus production and host immune regulation (Paone and Cani 2020, Pan et al. 2022). However, excessive mucin degradation can be harmful to the intestinal environment as it can disrupt the mucus layer and thereby provide direct access of the bacteria to the intestinal epithelial cell layer (Pan et al. 2022). As a first protection against extensive degradation by the microbiota, mucin glycans are often decorated with terminal substituents that can only be removed by specific bacterial enzymes (Raba and Luis 2023).

**Figure 1.**
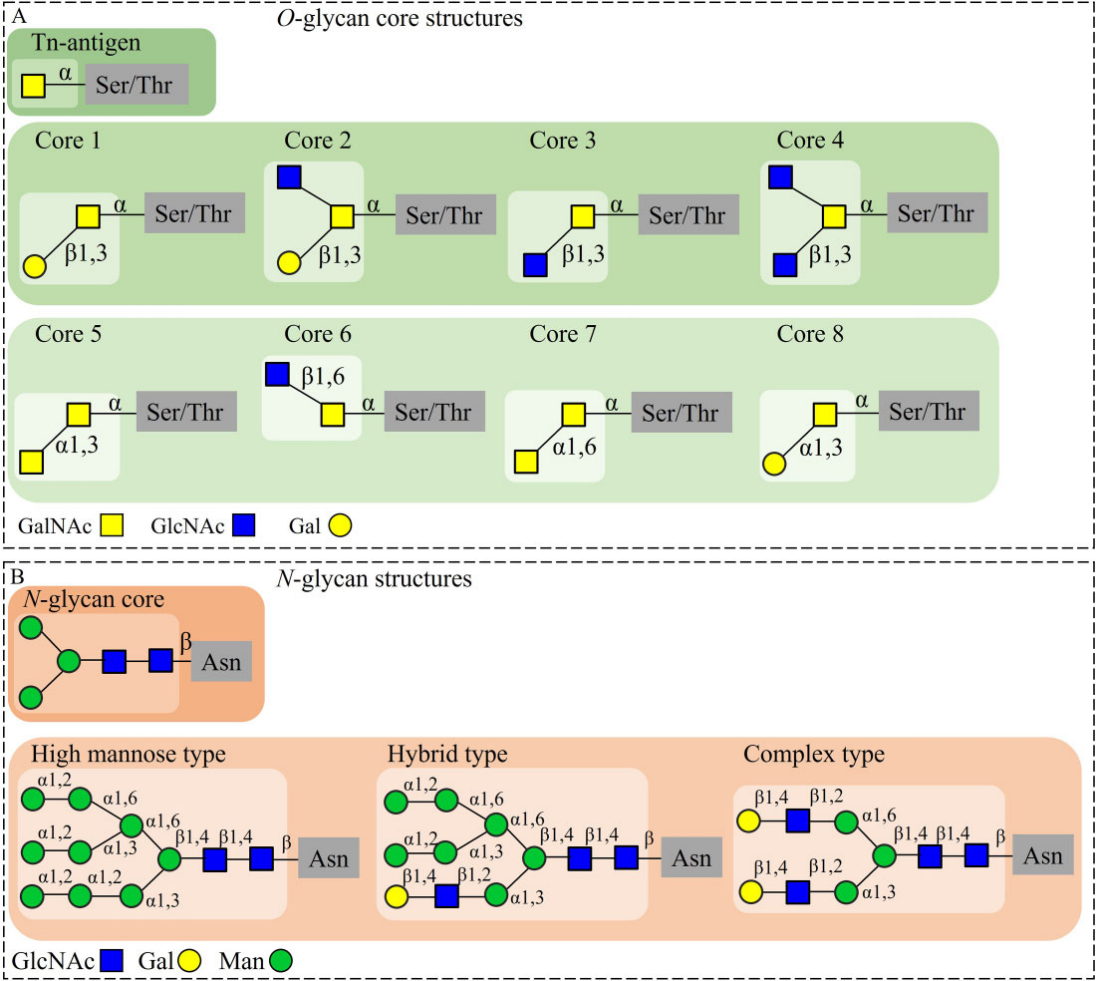
(A) *O-*glycan core structures present in mucins of the human GIT: the Tn-antigen (GalNAc-α-Ser/Thr) and the eight *O-*glycan core structures of which core structures 1–4 are the most abundant structures in mucins from the human GIT. (B) *N-*glycan structures present in mucins of the human GIT: the *N-*glycan core (GlcNAc_2_Man_3_-β-Asn) and the three recognized mammalian *N-*glycan structure types formed by elongation of the core by specific monosaccharide additions.

Mucin glycans mostly consist of glycans *O-*linked to the core protein (*O-*glycans) (Bell and Juge 2021). *O*-Glycosylation is initiated via an *N-*acetylgalactosamine (GalNAc) residue α-linked to the hydroxyl group of serine (Ser) or threonine (Thr) residues on mucin proteins (Fig. 1A), forming the Tn-antigen. Further glycosylation of the Tn-antigen through the GalNAc core residue results in eight different *O-*glycan core structures (Fig. 1). The core structures are defined by the substitution of galactose (Gal), *N-*acetylglucosamine (GlcNAc), and/or GalNAc units to the core GalNAc moiety and core structures can be further elongated by addition of Gal, GlcNAc, and GalNAc and decoration by terminal fucose (Fuc), sialic acid (Sia), or sulphate (Saldova and Wilkinson 2020). The majority of mucin *O-*glycans in the mucus layer of the human GIT is constituted by *O-*glycan core structures 1–4 (González-Morelo et al. 2020).

In addition to *O-*glycosylation, the mucin core protein is also *N-*glycosylated, although in low levels (Luis and Hansson 2023). Human colonic mucus, for example, contains ~50 times more *O-*glycans compared to *N-*glycans (Luis and Hansson 2023). In *N-*glycosylation, a GlcNAc unit is attached to the nitrogen atom of an asparagine (Asn) side chain, upon which another GlcNAc unit and three mannose (Man) units are attached, forming the *N-*glycan core (Fig. 1B) (Reily et al. 2019). By extension of this core structure, three (mammalian) *N-*glycan structure types are recognized: high-mannose type, hybrid type, and complex type (Fig. 1B). These structures can be further elongated by addition of Man, Gal, GlcNAc, or GalNAc sugars, after which the elongated branches can be extended by addition of terminal Fuc, Sia, or sulphate (Corfield 2018).

Both mucin *O-* and *N-*glycans can function as signalling molecules, as a binding site for the intestinal microbiota occupying the mucus layer, and as a carbon source for bacterial growth (Reily et al. 2019, Gao et al. 2021). Bacteria can degrade and utilize mucin glycans via a repertoire of specific carbohydrate active enzymes (Bell and Juge 2021, Drula et al. 2022). The monosaccharides released during mucin glycan degradation can be utilized by the bacteria themselves or they can be used by other intestinal bacteria present (cross-feeding) (Bell and Juge 2021). Bacterial species recognized for mucin glycan degradation and their ability to grow on mucin as sole carbon source (*in vitro*) include *A. muciniphila, Ruminococcus* spp., and *Bacteroides* spp. In addition, they also represent three of the important main phyla recognized in the human GIT (Geerlings et al. 2018). *A. muciniphila* (Verrucomicrobiota) and *Ruminococcus torques* (Bacillota) are known as mucin glycan-degrading specialists. Such specialists are adapted to the mucus environment and thrive on degradation of mucin glycans. *Bacter oides thetaiotaomicron* (Bacteroidota), in contrast, is known as a glycan-degrading generalist. Generalists, in comparison to specialists, can utilize a broader range of glycan sources including a wide variety of dietary glycans in addition to mucin glycans (Berkhout et al. 2022, Glover et al. 2022).

Previous research has shown that *A. muciniphila, R. torques*, and *B. thetaiotaomicron* are able to grow on porcine gastric mucin (PGM), a substrate often used as a model for human intestinal mucin (Hoskins et al. 1985a, Derrien et al. 2004, Kostopoulos et al. 2021, Davey et al. 2023, Schaus et al. 2024). *A. muciniphila* can completely degrade all PGM *O-*glycans, but cannot utilize all of the released monosaccharides and disaccharides for its own growth (Bakshani et al. 2025). It was recently proposed that *R. torques* is a keystone mucin degrader as it demonstrates broad mucin and *O-*glycan degradation abilities. The released glycans can be used for *R. torques* its own metabolism or they can be used by other species (Schaus et al. 2024). Although the growth of *B. thetaiotaomicron* on PGM is poor, it can degrade some of the PGM *O-*glycans and utilize released sugars (González-Morelo et al. 2020, Schaus et al. 2024, Bakshani et al. 2025). In addition, increased growth of *B. thetaiotaomicron* was observed when grown in co-culture with *A. muciniphila* or *R. torques* (Kostopoulos et al. 2021, Schaus et al. 2024). The above studies highlight that these intestinal bacteria employ different strategies for degradation and utilization of mucin glycans to thrive in the mucus layer (Tailford et al. 2015, Crouch et al. 2020, Bell and Juge 2021, Berkhout et al. 2022). This is most likely due to different enzymatic degradation abilities of glycosidic linkages by these bacteria towards *O-*glycans, as previously reported (Glover et al. 2022, Schaus et al. 2024). However, detailed insight into how the mucin glycans are degraded by these bacteria remains limited. Furthermore, the intrinsic relationship among the three mucin glycan degraders *A. muciniphila, R. torques*, and *B. thetaiotaomicron* in terms of mucin glycan degradation and utilization remains poorly understood.

This study investigated PGM *O-* and *N-*glycan degradation and utilization capabilities of the intestinal mucin glycan-degrading bacteria *A. muciniphila, R. torques*, and *B. thetaiotaomicron* in monoculture, co-culture, and as part of a mucin-degrading synthetic community (MDSC) (Berkhout et al. 2024). The focus of this study was on *O-*glycans as these are the primary substituents of mucins, but *N-*glycan degradation was also touched upon. *O*-Glycan degradation during 24 h incubation on PGM was monitored by porous graphitised carbon liquid chromatography coupled to mass spectrometry (PGC-LC-MS/MS) analysis of the remaining *O-*glycans after their release from PGM after (partial) fermentation. Utilization of PGM glycans was assessed based on the production of SCFAs and organic acids as fermentation metabolites. *N*-Glycan degradation was studied by matrix assisted laser desorption ionization time-of-flight-(MALDI-TOF)-MS analysis. In this way, we strived to broaden the understanding of the complex interactions occurring between mucin glycans and the intestinal microbiota.

## Materials and methods

### Chemicals

Water, acetonitrile (ACN), methanol, acetic acid, and trifluoroacetic acid (TFA) all ULC/MS-CC/SFC grade (≥99%) were obtained from Biosolve (Dieuze, France). 2,5-Dihydroxybenzoic acid (DHB; ≥99.5%) was obtained from Bruker (Bremen, Germany). Maltodextrin [degree of polymerization (DP) 1–20] from potato starch (≥99%) was obtained from Avebe (Veendam, The Netherlands). 1,4-α-d-Maltopentaose 99% (MP5, DP5), mucin type III from porcine stomach partially purified powder (PGM), ammonium bicarbonate (NH_4_HCO_3_) BioUltra ≥98%, sodium borohydride (NaBH_4_) caplets 98%, sodium chloride (NaCl) ≥98%, sodium hydroxide (NaOH) ≥98% pellets, sodium dodecyl sulphate (SDS) ≥97%, IGEPAL® CA-630 molecular biology grade, PNGase F enzyme from *Elizabethkingia meningoseptica* proteomics grade, and Supelco Supelclean Envi-Carb PGC solid phase extraction (SPE) cartridges (250 mg, 3 ml) were obtained from Sigma–Aldrich (Darmstadt, Germany). Sep-Pak Vac 6cc C18 reversed phase (RP) SPE cartridges (500 mg, 6 ml) were obtained from Waters (Eschborn, Germany). Sodium sulphate (Na_2_SO_4_) ≥99%, BGB 0.2 ml PP short thread vials 32 × 11.6 mm, and BGB ND9 short thread screw caps with slitted septa silicone/PTFE were obtained from Thermo Scientific (San Jose, CA, USA). Eppendorf® Safe-Lock microcentrifuge tubes (1.5 and 2.0 ml) were obtained from VWR (Darmstadt, Germany). Incubation of samples at set temperature was done using an Eppendorf Thermomixer Comfort (Oldenburg, Germany). A Thermo Scientific Savant centrifugal evaporator was used for drying the samples. For SPE, a Vacuum manifold from Waters (Massachuttes, MA, USA) and a vacuum gas pump from VWR were used.

### Bacterial strains and mucin medium preparation

Individual bacterial strains used in this study included *A. muciniphila* (DSM 22959), *R. torques* (ATCC 29149), and *B. thetaiotaomicron* (DSM 2079). Co-cultures thereof were prepared in a 1:1 ratio (v/v, *A. muciniphila* and *R. torques, A. muciniphila* and *B. thetai otaomicron, R. torques* and *B. thetaiotaomicron, A. muciniphila* and *R. torques* and *B. thetaiotao micron*). The MDSC used in this study contained *A. muciniphila* (DSM 22959), *Ruminococcus gnavus* (ATCC 29149), *R. torques* (ATCC 27756), *Bacteroides fragilis* (DSM 2151), *B. thetaiotaomicron* (DSM 2079), *Phocaeicola vulgatus* (ATCC 8482), *Bacteroides caccae* (DSM 19024), *Anaerostipes caccae* (DSM 14662), *Agathobacter rectalis* (ATCC 33656), *Faecalibacterium duncaniae* (DSM 17677), *Roseburia intestinalis* (DSM 14610), *Desulfovibrio piger* (DSM 749), and *Blautia hydrogenotrophica* (DSM 10507) (Berkhout et al. 2024).

Bacterial monocultures, co-cultures, and the MDSC were cultured in serum bottles with a 80:20 N_2_:CO_2_ headspace pressurized at 1.5 atmospheric pressure (atm) and basal anoxic medium supplemented with 1 g/l yeast extract, prepared according to Plugge (2005). The carbon source was either crude type III PGM (0.5% w/v, described below), glucose (Glc, 20 mM), or GlcNAc (20 mM). All cultures were incubated at 37°C without shaking (Berkhout et al. 2024).

A 5% (w/v) crude mucin stock solution was prepared by stirring 5.0 g of type III PGM at room temperature for 60 min. Subsequently, the solution was centrifuged at 10 000 × *g* for 10 min. The supernatant was dispersed in serum bottles, capped with butyl rubber stoppers, gas exchanged to a N_2_ gas phase, and autoclaved for 20 min at 121°C. The final PGM concentration in the medium was 0.5% (w/v). Glc and GlcNAc were filter sterilized prior to addition to the medium (Berkhout et al. 2024).

### Preparation of the bacterial pre-cultures

Pre-cultures of *B. caccae, B. fragilis, B. thetaiotaomicron, P. vulgatus, R. gnavus, R. torques, A. caccae, F. duncaniae, A. rectalis*, and *B. hydrogenotrophica* were prepared with Glc as the carbon source, whereas *A. muciniphila* was pre-cultured on GlcNAc supplemented with 3 g/l l-Thr, and *D. piger* was precultured with 20 mM sodium sulphate (Na_2_SO_4_, 99+%, Acros Organics/Thermo Fisher Scientific) and a 80:20 H_2_:CO_2_ headspace pressurized at 1.5 atm. In addition, pre-cultures of *A. muciniphila* and *R. torques* were prepared using crude PGM (0.5% w/v) as the carbon source (Berkhout et al. 2024).

### Bacterial growth experiment conditions

A total of 11 combinations of microbes and carbon sources were inoculated in triplicate at a volume of 1% (v/v) bacteria and incubated for 24 h at 37°C. For *A. muciniphila* and *R. torques*, the pre-cultures grown on PGM were used for inoculation in conditions with mucin as the carbon source. Between 0 and 12 h incubation, sampling was performed every 3 h for glycan analysis and a final sampling was performed after 24 h. The samples were stored at −20°C. Then, the samples for glycan analysis were autoclaved for 20 min at 121°C and stored at −20°C until further use. Microbial growth was followed by OD600 measurement (Implen OD600 DiluPhotometer, München, Germany). As negative controls, similar experiments were performed using crude PGM medium without bacteria and using GlcNAc/Glc as the carbon source with *A. muciniphila, R. torques*, or *B. thetaiotaomicron*. Relative abundance of bacteria was measured at 12 and 24 h by 16S rRNA gene amplicon sequencing based on previously described protocol by Shetty et al. (2022) (number of 16S rRNA gene copies per bacterium is described in Supporting information (SI) Table S1) (Berkhout et al. 2024). The total bacterial abundance was determined by quantitative polymerase chain reaction (qPCR) as described previously by Berkhout et al. (2024). Standards were prepared with *B. fragilis* DNA by amplification of the 16S rRNA gene, samples were diluted to 1 ng/uL, and qPCR was performed with primers BACT-F-1369 (5′-CGGTGAATACGTTCYCGG) and PROK-R-1492 (5′-GGWTACCTTGTTACGACTT) according to Suzuki et al. (2000) and Berkhout et al. (2024). Results were analysed with CFX Manager v3.1 (Bio-Rad) to calculate the 16S rRNA gene copy number/µl as described by Berkhout et al. (2024).

### Analysis of metabolites produced during bacterial growth on PGM

High-performance liquid chromatography (HPLC) was used to measure SCFAs, other organic acids, and ethanol (EtOH) produced during the incubation described in the 'Bacterial growth experiment conditions' section as an indicator of the activity of the bacteria (three replicates for all timepoints, except for timepoint 3 h of replicates 2 and 3). The protocol was based on a previously described method by Kong et al. (2021). Bacterial supernatant samples (autoclaved) were diluted three times with ultra-pure water and 10 µl was injected onto the HPLC system. The analysis was performed using an Ultimate 3000 HPLC system (Dionex, Sunnyvale, USA) in combination with an Aminex HPX-87H column (Bio-Rad Laboratories Inc., Hercules, CA, USA). Acids were detected by a refractive index detector (RI-101, Shodex, Yokohama, Japan) and a UV detector set at 210 nm (Dionex Ultimate 3000 RS variable wavelength detector). Elution of the compounds was performed at 0.6 ml/min and 40°C using 50 mM sulphuric acid. Calibration curves of succinate, formate, acetate, 1,2-propanediol, propionate, butyrate, EtOH, and isovalerate (0.001–4 mg/mL, *R*^2^ ≥ 0.98) were used for quantification. Data analysis was performed with Chromeleon^TM^ 7.3.1 software from Thermo Fisher Scientific (Waltham, MA, USA).

### *O*-Glycan release using reductive β-elimination and clean-up by SPE

*O-*Glycans were released and clean-up was performed according to de Ram et al. (2024). In short, 750 µl of 1 M NaBH_4_ in 0.05 M NaOH was added to 250 µl of each autoclaved bacterial supernatant sample (three replicates for all timepoints, except for timepoints 0 and 3 h of replicate 3). Samples were homogenized, 1 µl of 1 mg/mL MP5 was added as internal standard (IS), and samples were incubated overnight (18–22 h) at 40°C at 300 rpm in a Thermomixer. Then, samples were acidified with 30 µl of glacial acetic acid and clean-up was performed using C18 and PGC SPE. As method control, crude PGM was used and the inoculation controls (described in the ‘Bacterial growth experiment conditions’ section) were included as well. Reproducibility as well as minimal variation (SD < 3.0%) of the method was shown previously (de Ram et al. 2024).

### *N*-Glycan release using enzymatic treatment

*N-*Glycans were released and clean-up was performed according to de Ram et al. (2024). In short, 350 µl of 200 mM ammonium bicarbonate and 65 µl of 2% SDS were added to 250 µl of each autoclaved bacterial supernatant sample (three replicates). Samples were homogenized, 1 µl of 1 mg/mL MP5 was added as the IS, and proteins were denatured for 10 min at 65°C. Then, 100 µl 200 mM ammonium bicarbonate, 100 µl 4% IGEPAL, and 15 µl PNGase F enzyme (100 U/mL) were added and samples were incubated at 37°C overnight (18–22 h). Thereafter, EtOH (100%) was added to yield a 80% EtOH solution. The solutions were stored at −20°C for 4 h, then centrifuged at 14 000 × *g* for 30 min and the supernatant was purified by PGC SPE as described previously (de Ram et al. 2024).

### Analysis of released *O*-glycans present in bacterial supernatants by porous graphitized carbon-LC-MS/MS

To follow *O-*glycan degradation by the bacteria over time, released PGM *O-*glycans (see the ‘Co-occurrence network analyses of 16S rRNA and 18S rRNA ASVs’ section) were analysed by PGC-LC-MS/MS according to de Ram et al. (2024). In short, samples were injected onto a Vanquish ultra-high-pressure LC system (Thermo Scientific) equipped with a PGC Hypercarb guard column (10 × 2.1 mm, particle size 3 µM, Thermo Scientific) and a PGC Hypercarb analytical column (150 × 2.1 mm, particle size 3 µM, Thermo Scientific). The mobile phases used were 10 mM ammonium bicarbonate (NH_4_HCO_3_) in water (A) and 10 mM NH_4_HCO_3_ in ACN:water 40:60 (B), and *O-*glycans were eluted with a flow rate of 200 µl/min using a gradient from 2% B to 60% B in 40 min after which the column was equilibrated for 10 min to starting conditions (2% B). The set-up was coupled via a HESI source to a Velos Pro ion trap MS (Thermo Scientific). Data acquisition was performed in negative ion mode (full MS and MS/MS data dependent). Data analysis is elaborated on in the ‘Glycan representation, MS data analysis, and construction of the heatmap’ section.

### Analysis of released *N*-glycans present in bacterial supernatants by MALDI-TOF MS analysis

To follow the *N-*glycan degradation by the bacteria over time, the released PGM *N-*glycans (see the ‘*N*-glycan release using enzymatic treatment’ section) were analysed by MALDI-TOF MS analysis according to de Ram et al. (2024). In short, 1 µl of matrix (DHB 20 mg/mL in 50:50 ACN:water + 0.1% TFA) was spotted manually on a MALDI MTP target plate (Bruker Daltonics, Bremen, Germany) followed by addition of 1 µl of sample. The spots were dried using a hairdryer. MALDI-TOF MS measurements were performed in positive ion mode on an Autoflex MaX instrument (Bruker Daltonics, Bremen, Germany). Data analysis is elaborated in the ‘Glycan representation, MS data analysis, and construction of the heatmap’ section.

### Glycan representation, MS data analysis, and construction of the heatmap

GlycoWorkBench version 1.1 was used for glycan representation and visualization. The glycan structures were represented according to the Symbol Nomenclature for Glycans (Varki et al. 2015). Representative glycan structures were exported from GlycoWorkBench and used in the figures for visualization and clarity of the structures identified in MS data. *O-*Glycan structures were assigned based on careful review of the full MS and fragmentation (MS/MS) data and review of literature regarding mucin *O-*glycans (Hayes et al. 2011, Jin et al. 2017, 2019, Bechtella et al. 2024). *N*-Glycan structures were assigned based on careful review of the MALDI-TOF mass spectra and review of literature regarding mucin *N-*glycans (Van Der Post et al. 2014, Gallego et al. 2023). Glycan MS data analysis was performed in a step-wise manner. First, a qualitative analysis was performed to compare the type of glycan identified by MS among the different samples. Second, a semi-quantitative approach was used to compare the type of glycans present in each sample. For this, the glycan ratios and intensities of the identified glycans were used. The glycan ratios were calculated based on the total peak area (*O-*glycans) or peak intensity (*N-*glycans) of identified glycans and compared between the samples. The *O-*glycan intensities were based on peak areas after manual peak integration using Xcalibur Qual Browser and Xcalibur Quan Browser software v4.5 (Thermo Scientific). The *N-*glycan intensities were based on peak height after manual peak integration using flexAnalysis v1.4 (Bruker). Lastly, the peak area or peak intensity of added IS MP5 was reviewed for each sample and consequently compared with the peak area or intensity of the other identified glycan peaks. This was used for reproducibility and semi-quantitative purposes. The constructed heatmaps were made with GraphPad Prism version 9.3.1. For each sample, the intensity at 0 h was set to 100% and the intensities (based on peak area) at timepoints 3, 6, 9, 12, and 24 h were set relative to this 100%. Glycan degradation was then visualized by comparing the increase/accumulation or decrease at the timepoints relative to 0 h. *O-*Glycan degradation of two biological replicates (designated R1 and R2) was visualized by using the average (individual heatmaps, including R3 in which case 6 h was set to 100%, are shown in the SI). Comparison of fucosylated/non-fucosylated and confirmed core 1, 2, and 4 PGM *O*-glycans was visualized in a bar graph by showing the relative abundance based on the total intensities of each condition. Furthermore, the percentage of each condition compared to the total sum (100%) was visualized as well in a bar graph. The values used were averages of three biological replicates and error bars are shown based on the standard deviation of three biological replicates. For the comparison of fucosylated/non-fucosylated *O*-glycans, suspected chemical degradation product GalNAcα1–3(Fucα1–2)Gal was omitted, and for the comparison of core 1, 2, and 4 PGM *O*-glycans, only non-fucosylated and *O*-glycans ≥*m/z* 587.3 were selected.

## Results and discussion

To study microbial mucin glycan degradation and utilization, monocultures of *A. muciniphila, R. torques*, and *B. thetaiotaomicron*, co-cultures thereof, and an MDSC containing intestinal mucin glycan-degrading bacteria and cross-feeders (as described in the ‘Bacterial strains and mucin medium preparation’ section) were grown on PGM as the sole carbon source during 24 h. Mucin *O-* and *N-*glycans remaining on the protein backbone were chemically or enzymatically released and analysed at different timepoints to study mucin glycan degradation by the bacteria. Analysis of metabolites produced by the bacteria during fermentation on PGM was performed to understand the utilization of mucin glycans.

### Bacterial growth on PGM is determined by the bacterial composition

Bacterial growth was measured by optical density (OD600) from 0 to 24 h for *A. muciniphila, R. torques*, and *B. thetaiotaomicron* in monoculture. (SI Fig. S1 illustrates bacterial growth curves based on OD600.) The increase in OD600 between 0 and 12 h indicates growth of the studied bacteria on PGM as their sole carbon source. The observed growth of *B. thetaiotaomicron* was minimal compared to *A. muciniphila* and *R. torques* (12 h) as confirmed by qPCR analysis. (SI Fig. S2 shows mean qPCR results.) Co-cultures of the three bacteria as well as the MDSC could also grow on PGM as sole carbon source. The co-cultures grew faster and reached a higher cell count compared to their respective monocultures (SI Figs S1 and S2). The presence and relative abundance of each bacterium in the co-cultures and the synthetic community was confirmed by 16S rRNA gene amplicon sequencing. (SI Fig. S3 demonstrates the relative abundances in each culture.)

### PGM *O*-glycan degradation by *A. muciniphila, R. torques*, and *B. thetaiotaomicron* as monocultures, co-cultures, and as part of an MDSC

**Figure 2.**
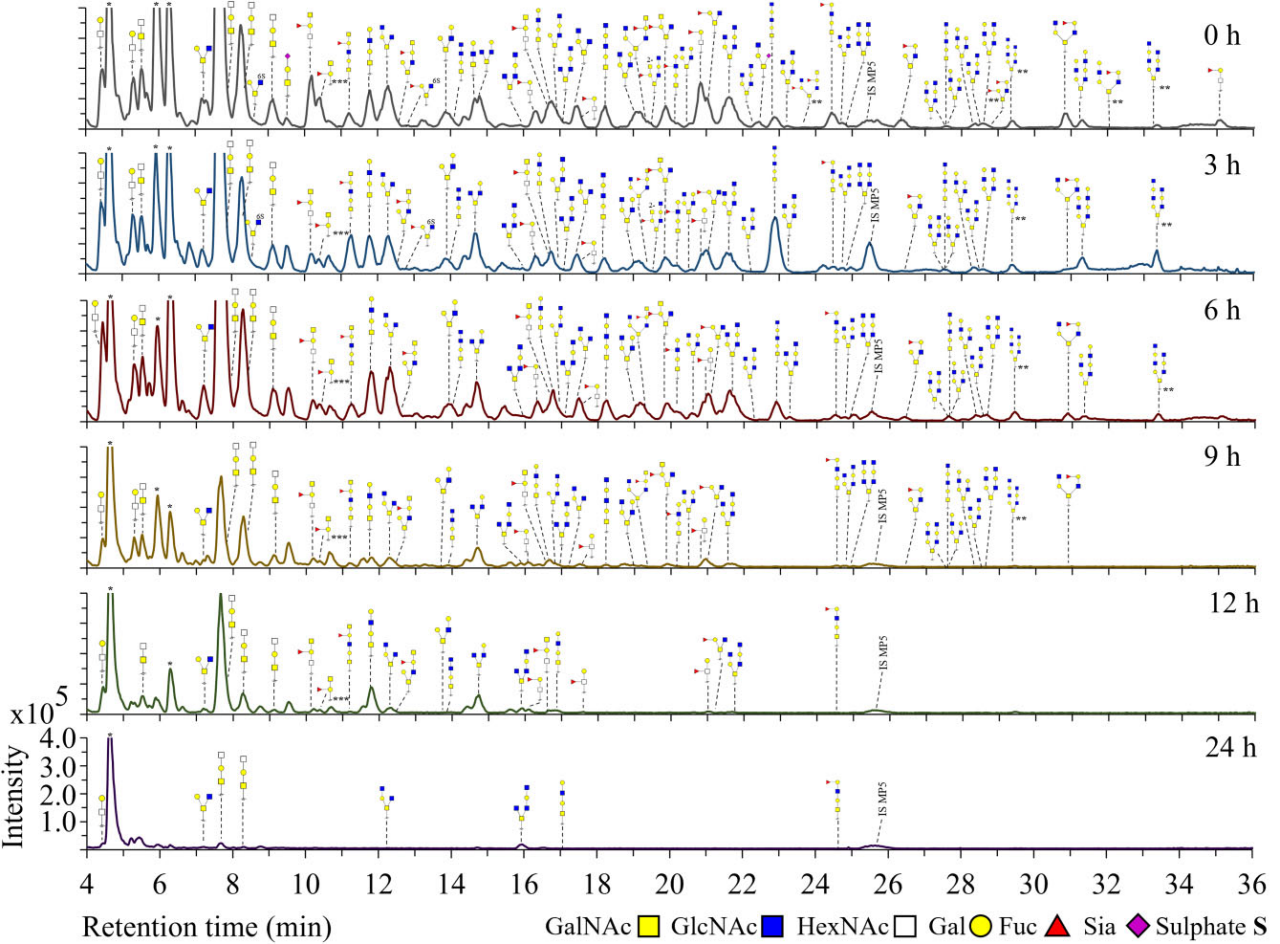
PGC-LC-MS elution patterns showing *O-*glycan structures remaining on the protein backbone after incubation of PGM with *A. muciniphila* for 24 h (replicate 1). *O*-Glycans were released by reductive β-elimination prior to analysis. Structures of *O-*glycans are based on MS/MS fragmentation data. IS MP5: internal standard maltopentaose DP5. *Peaks could not be assigned to PGM *O-*glycans based on *m/z* and MS/MS fragmentation data. **Structure is a suggestion based on *m/z* and the MS/MS fragmentation pattern. ***Structure is suspected to have emerged from chemical degradation.

**Figure 3.**
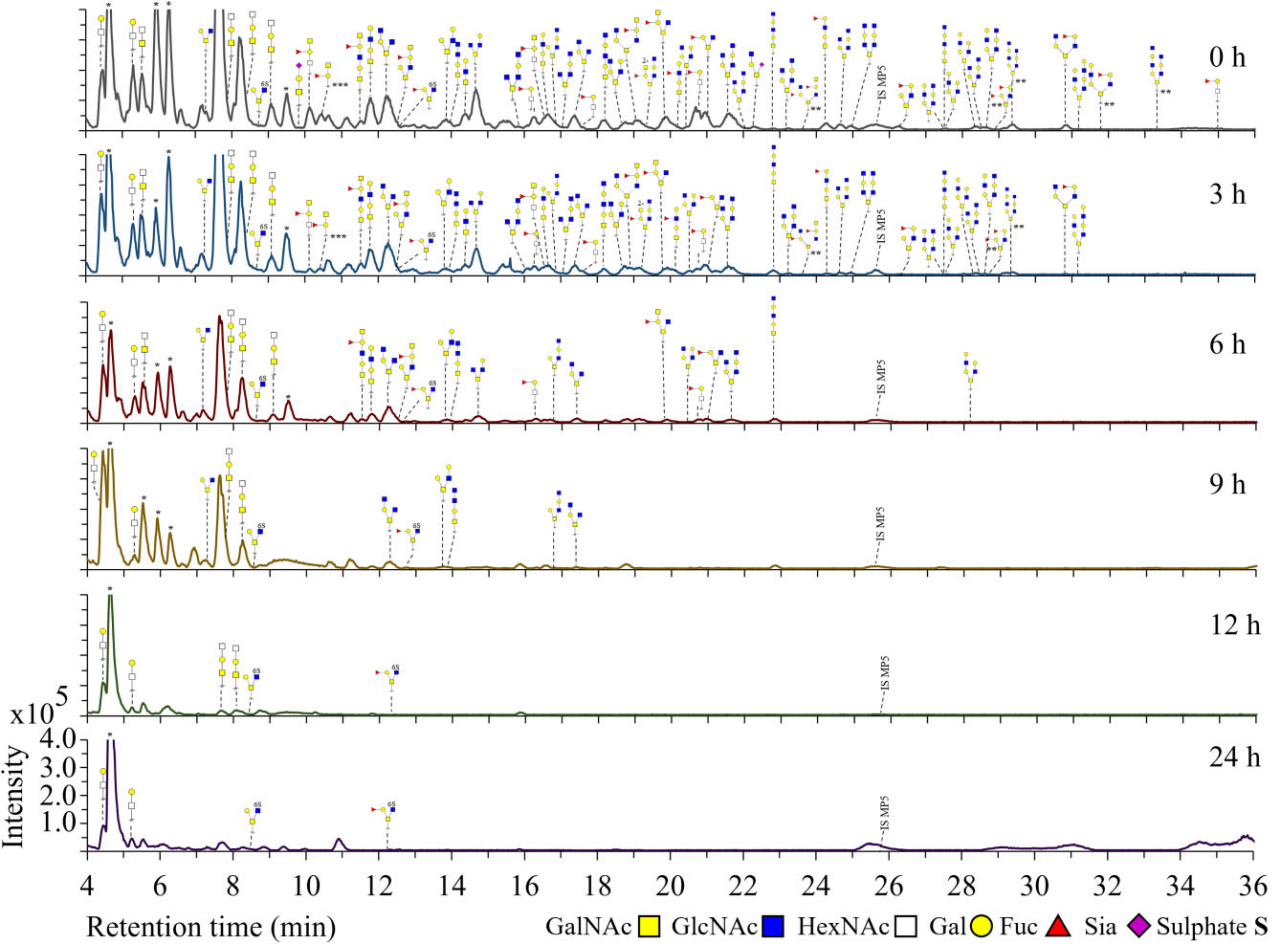
PGC-LC-MS elution patterns showing *O-*glycan structures remaining on the protein backbone after incubation of PGM with *R. torques* for 24 h (replicate 1). *O*-Glycans were released by reductive β-elimination prior to analysis. Structures of *O-*glycans are based on MS/MS fragmentation data. IS MP5: internal standard maltopentaose DP5. *Peaks could not be assigned to PGM *O-*glycans based on *m/z* and MS/MS fragmentation data. **Structure is a suggestion based on *m/z* and the MS/MS fragmentation pattern. ***Structure is suspected to have emerged from chemical degradation.

**Figure 4.**
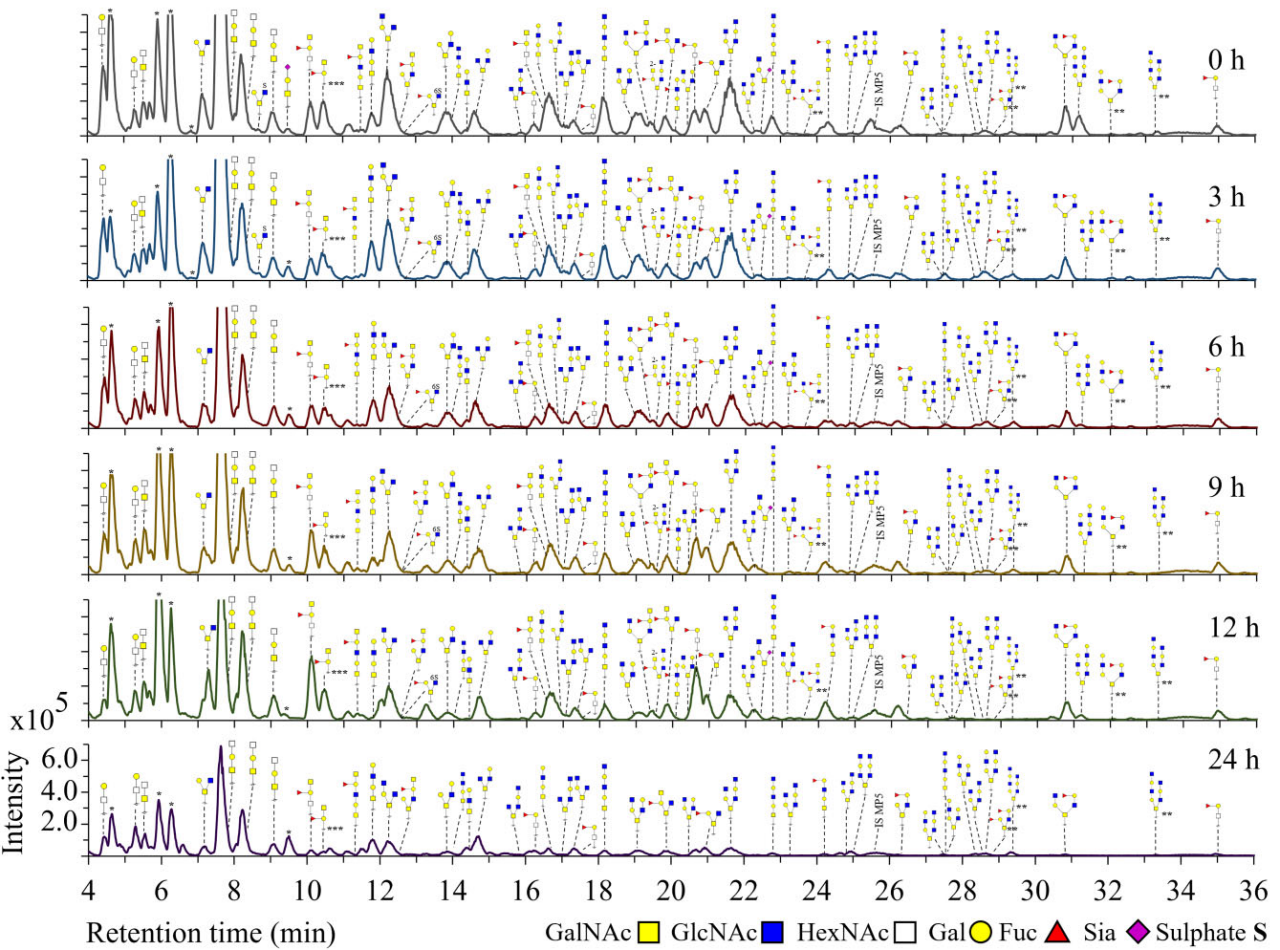
PGC-LC-MS elution patterns showing *O-*glycan structures remaining on the protein backbone after incubation of PGM with *B. thetaiotaomicron* for 24 h (replicate 1). *O*-Glycans were released by reductive β-elimination prior to analysis. Structures of *O-*glycans are based on MS/MS fragmentation data. IS MP5: internal standard maltopentaose DP5. *Peaks could not be assigned to PGM *O-*glycans based on *m/z* and MS/MS fragmentation data. **Structure is a suggestion based on *m/z* and the MS/MS fragmentation pattern. ***Structure is suspected to have emerged from chemical degradation.

**Figure 5.**
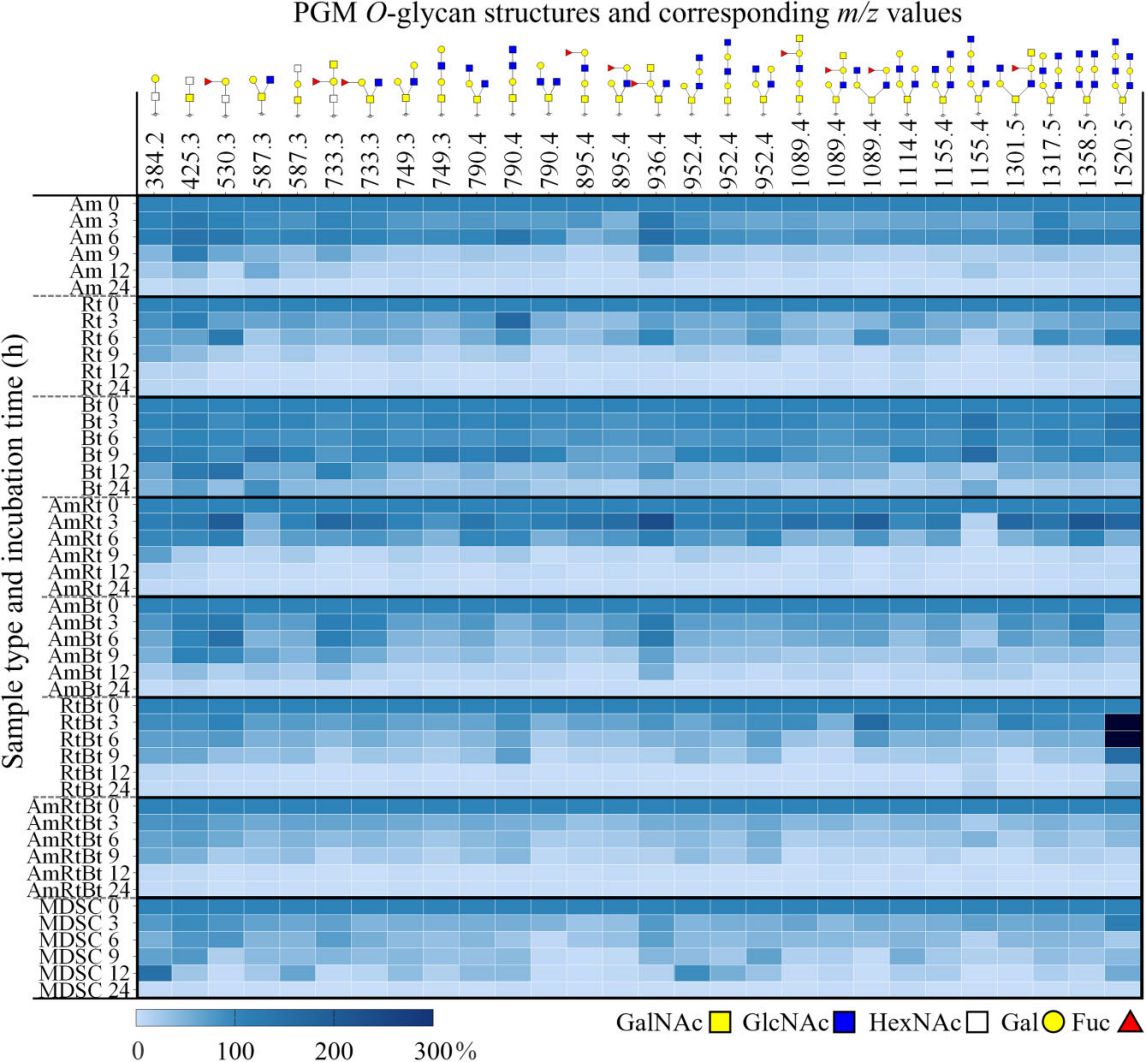
Heatmap of the foremost abundant mucin *O-*glycans resulting from PGM incubation with monocultures *A. muciniphila* (Am), *R. torques* (Rt), *B. thetaiotaomicron* (Bt), co-cultures (AmRt, AmBt, RtBt, AmRtBt), and the MDSC, demonstrating the degradation of *O-*glycans over 24 h incubation. The intensity values (obtained from the peak areas, average of three replicates) of the *O-*glycans at 0 h were set to 100% and the intensity values of 3–24 h are shown relative to the values at 0 h. The left y-axis displays the bacterial culture and the sampling time (h). The top *x*-axis displays the *m/z* values of the corresponding [M-H]^−^  *O-*glycan structures. *O*-Glycans structures are based on acquired fragmentation data (PGC-LC-MS/MS). Sialylated *O-*glycan structures are not shown due to their low abundance and complete degradation within 3–6 h by each culture.

**Figure 6.**
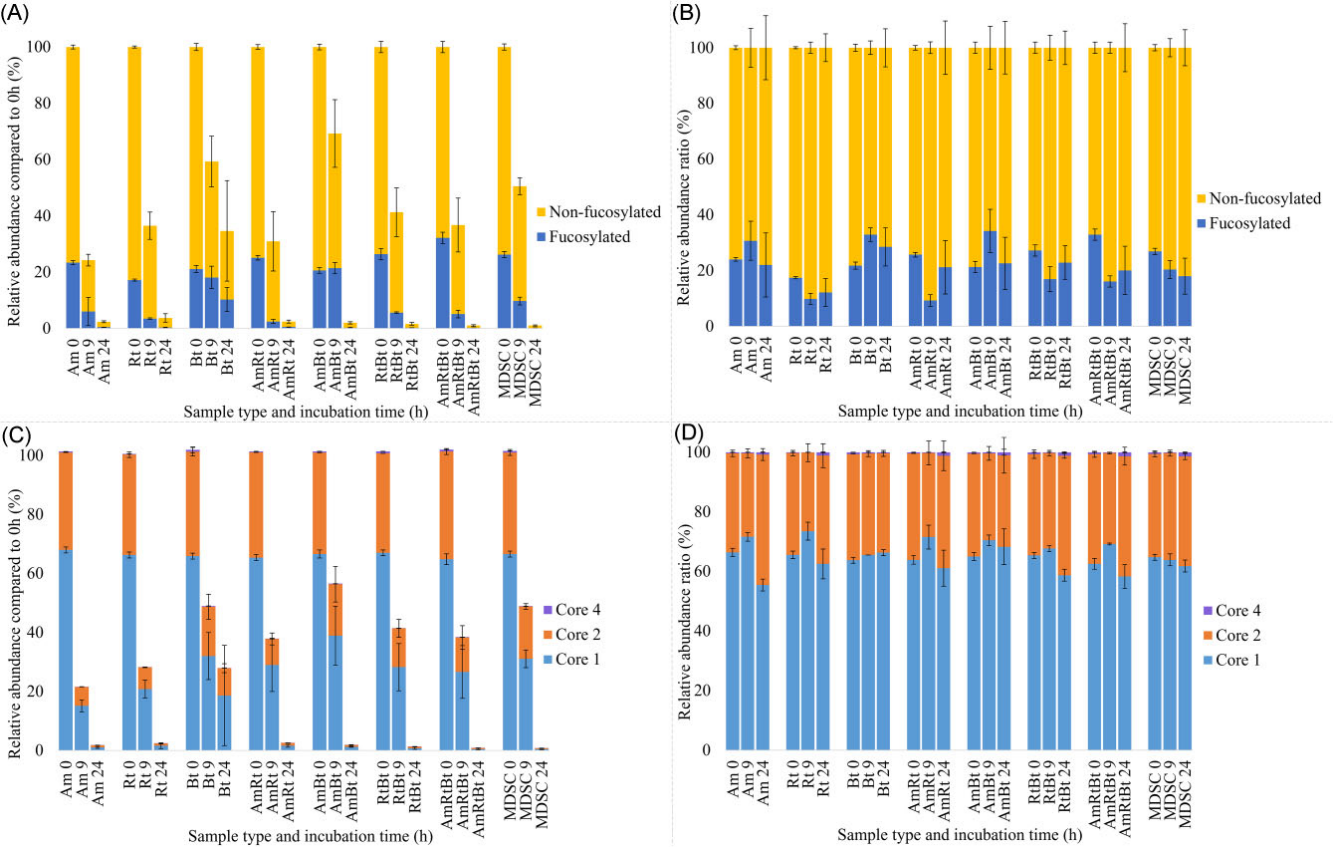
Relative abundance of identified fucosylated and non-fucosylated PGM *O*-glycans and identified core 1, 2, and 4 *O*-glycan structures (only non-fucosylated *O*-glycans and *O*-glycans ≥ *m/z* 587 were selected) based on intensity of the integrated peaks of biological triplicates (values are shown as average ± standard deviation). (A) Relative abundance of identified fucosylated and non-fucosylated PGM *O*-glycans expressed as percentage relative to sum of fucosylated and non-fucosylated *O*-glycans at 0 h per bacterial condition. (B) Percentage of identified fucosylated and non-fucosylated PGM *O*-glycans expressed relative to the sum of fucosylated and non-fucosylated PGM *O*-glycans (100%) per bacterial condition. (C) Relative abundance of identified core 1, 2, and 4 *O*-glycan structures expressed as percentage relative to sum of the core 1, 2, and 4 *O*-glycans at 0 h per bacterial condition. (D) Percentage of identified core 1, 2, and 4 *O*-glycan structures as part of sum of the core 1, 2, and 4 *O*-glycans structures (100%) per bacterial condition.

PGM *O-*glycan degradation by *A. muciniphila, R. torques, B. thetaiotaomicron*, bacterial co-cultures, and the MDSC was analysed using PGC-LC-MS/MS. The degradation of all identified *O-*glycans was compared in detail between the three monocultures (Figs 2–4). Then, the degradation of the foremost abundant *O-*glycans was summarized in a heatmap (Fig. 5) by comparison of the peak area of each *O-*glycan for each individual condition relative to the peak area obtained at 0 h set to 100% (based on the PGC-LC-MS *O-*glycan profiles as shown in Figs 2–4 and SI Figs S4–S8). Moreover, the abundance of fucosylated and non-fucosylated *O*-glycans and different core structures (cores 1–4) was done by comparing their relative abundances over time (Fig. 6). The results are discussed in the following sections. Some structures could potentially emerge as chemical degradation products (0 h) resulting from the sample preparation. Therefore, these structures are visualized using blank symbols if there is discrepancy regarding the monomer (e.g. GalNAc or GlcNAc).

#### *R. torques* shows PGM O-glycan degradation with preference for fucosylated *O*-glycans, whilst *A. muciniphila* and *B. thetaiotaomicron* show a more gradual and generic degradation of PGM *O*-glycans

*A. muciniphila* degraded most of the *O-*glycans after 9 h (Fig. 2, R1, 0–9 h, e.g. fucosylated *m/z* 530.3 GlcNAc_1_Gal_1_Fuc_1_/GalNAc_1_Gal_1_Fuc_1_, *m/z* 587.3 GalNAc_2_Gal_1_/GalNAc_1_GlcNAc_1_Gal_1_, *m/z* 733.3 GalNAc_2_Gal_1_Fuc_1_/GalNAc_1_GlcNAc_1_Gal_1_Fuc_1_, *m/z* 790.4 GalNAc_1_GlcNAc_2_Gal, *m/z* 1098.4 GalNAc_1_GlcNAc_2_Gal_2_Fuc_1_, and *m/z* 1155.4 GalNAc_1_GlcNAc_3_Gal_2_). Both fucosylated and non-fucosylated *O*-glycans were degraded in equal ratios (Fig. 6A and B, Am, 0–24 h). Although the level of sialylation and sulphation reported for PGM is low (Nordman et al. 1997, Jin et al. 2019), sialylated (*m/z* 675.3 Siaα2–3Galβ1–3GalNAc, *m/z* 878.3 Siaα2–6(GlcNAcβ1–3Galβ1–3)GalNAc) and sulphated (*m/z* 667.2 6S-GlcNAcβ1–6(Galβ1–3)GalNAc and *m/z* 813.3 6S-GlcNAcβ1–6(Fucα1–2Galβ1–3)GalNAc) *O-*glycans were degraded by *A. muciniphila* within 6 h (Fig. 2). No specific preference was observed for *A. muciniphila* towards identified core 1 (linear) or core 2 (branched) structures as illustrated by equal degradation of isomers *m/z* 749.3 (Galβ1–4GlcNAcβ1–3Galβ1–3GalNAc and Galβ1–4GlcNAcβ1–6(Galβ1–3)GalNAc) and isomers *m/z* 952.4 (GlcNAcβ1–3Galβ1–4GlcNAcβ1–3Galβ1–3GalNAc, GlcNAcβ1–3Galβ1–4GlcNAcβ1–6(Galβ1–3)GalNAc, and Galβ1–4GlcNAcβ1–6(GlcNAcβ1–3Galβ1–3)GalNAc). When comparing the ratio of core 1 to core 2 structures, however, core 2 structures decreased more overtime compared to core 1 structures (Fig. 6C and D, Am, 0–9 h). The low amount of *O*-glycans left after 24 h should be taken into account when reflecting on the ratios after 24 h (Fig. 6C, Am, 24 h). The increase in intensity of Gal_1_GalNAc_1_/Gal_1_GlcNAc_1_ (*m/z* 384.2) and GlcNAc_1_GalNAc_1_/GalNAc_1_GalNAc_1_ (*m/z* 425.2) after 6–9 h is hypothesised to originate from degradation of longer structures. After 24 h incubation, low abundance of various *O-*glycans (RT 4–17 min, ≤5 glycosyl residues) was still observed suggesting that these glycans (linkages) are not the preferred substrate for *A. muciniphila*.

*R. torques* efficiently degraded fucosylated *O-*glycans within 3 h (Fig. 3, R1, e.g. 530.3 GlcNAc_1_Gal_1_Fuc_1_/GalNAc_1_Gal_1_Fuc_1_, *m/z* 733.3 GalNAc_2_Gal_1_Fuc_1_/GalNAc_1_GlcNAc_1_Gal_1_Fuc_1_, *m/z* 1098.4 GalNAc_1_GlcNAc_2_Gal_2_Fuc_1_) compared to 6 h for most non-fucosylated *O-*glycans (Fig. 3, e.g. *m/z* 587.3 GalNAc_2_Gal_1_/GalNAc_1_GlcNAc_1_Gal_1_, *m/z* 790.4 GalNAc_1_GlcNAc_2_Gal_1_, *m/z* 1155.4 GalNAc_1_GlcNAc_3_Gal_2_). After 6 h, the majority of fucosylated *O-*glycans was completely degraded by *R. torques*, which is also illustrated in Fig. 6A and B showing a decrease in ratio of fucosylated *O*-glycans compared to non-fucosylated *O*-glycans (Rt, 0–24 h). No specific preference was observed for *R. torques* towards identified core 1 or core 2 structures, as illustrated by the degradation of isomers *m/z* 749.3 (Galβ1–4GlcNAcβ1–3Galβ1–3GalNAc and Galβ1–4GlcNAcβ1–6(Galβ1–3)GalNAc) and isomers *m/z* 952.4 (GlcNAcβ1–3Galβ1–4GlcNAcβ1–3Galβ1–3GalNAc, GlcNAcβ1–3Galβ1–4GlcNAcβ1–6(Galβ1–3)GalNAc, and Galβ1–4GlcNAcβ1–6(GlcNAcβ1–3Galβ1–3)GalNAc). When comparing the ratio of core 1 to core 2 structures, however, core 2 structures decreased more overtime than core 1 structures (Fig. 6C and D, Rt, 0–9 h). For *R. torques* as well, the low amount of *O*-glycans remaining after 24 h should be taken into account when reflecting on the ratios after 24 h (Fig. 6C, Rt, 24 h). The present sialylated *O-*glycans were degraded within 3 h by *R. torques*, demonstrating high enzyme production and activity of *R. torques* towards Sia removal (Bell et al. 2019, Crost et al. 2023). The identified sulphated *O-*glycan (*m/z* 667.3) was not degraded by *R. torques*, which can be explained by the absence of sulphatases (Drula et al. 2022, Schaus et al. 2024). After 24 h incubation, only *O-*glycans Gal_1_GalNAc_1_/Gal_1_GlcNAc_1_ (*m/z* 384.2, RT 4–5 min) and GlcNAc_1_GalNAc_1_/GalNAc_1_GalNAc_1_ (*m/z* 425.3, RT 4–5 min) were still observed suggesting that these glycans (linkages) are not the preferred substrate for *R. torques*.

*B. thetaiotaomicron* showed minimal degradation of PGM *O-*glycans within 9 h incubation but after 12 h, *B. thetaiotaomicron* was able to degrade many of the identified *O-*glycans (Fig. 4, R1, 0–12 h). Both fucosylated and non-fucosylated *O*-glycans were degraded by *B. thetaiotaomicron* but the ratio of non-fucosylated decreased compared to fucosylated overtime (Fig. 6A and B, Bt, 0–24 h, Fig. 4, e.g. fucosylated *O-*glycans *m/z* 733.3 GalNAc_2_Gal_1_Fuc_1_/GalNAc_1_GlcNAc_1_Gal_1_Fuc_1_ and *m/z* 1098.4 GalNAc_1_GlcNAc_2_Gal_2_Fuc_1_ and non-fucosylated *O-*glycans *m/z* 790.4 GalNAc_1_GlcNAc_2_Gal_1_ and *m/z* 1155.4 GalNAc_1_GlcNAc_3_Gal_2_). *Bacteroides thetaiotaomicron* degraded both core 1 and core 2 structural isomers equally (e.g. *m/z* 587.3 GalNAc_1_GlcNAc_1_Gal_1_ and *m/z* 952.4 GalNAc_1_GlcNAc_2_Gal_2_) (Fig. 6C and D, Bt, 0–24 h). *Bacteroides thetaiotaomicron* did not show degradation of *m/z* 425.2 (GlcNAcβ1–3GalNAc) within 24 h, which could indicate absence of enzymes that can cleave the GlcNAc_1_GalNAc_1_/GalNAc_1_GalNAc_1_linkage or this specific configuration. *Bacteroides thetaiotaomicron* could degrade the present sialylated and sulphated *O-*glycans within 6 h indicating high enzyme production and activity for removal of these terminal substituents in accordance with literature (Luis et al. 2018, Crouch et al. 2020).

#### Co-cultures and the MDSC show different and more complete PGM *O*-glycan degradation

Different and more complete *O-*glycan degradation profiles were observed after incubation of the co-cultures and the MDSC on PGM for 24 h compared to the monocultures. (Figs 5 and6 and SI Figs S4–S8 show the PGM-LC-MS chromatograms of R1, SI Figs S9–S11 show the individual heatmaps of R1, R2, and R3, and SI Fig. S12 show the fragmentation spectra used for assigning linear and branched structures to the identified *O*-glycans.) Co-culture *A. muciniphila*/*R. torques* (AmRt) degraded all *O-*glycans within 9 h with preferential degradation of fucosylated *O-*glycans (Fig. 5, AmRt, 0–6 h, e.g. *m/z* 1089.4, Fig. 6A and B, AmRt, 0–24 h, and SI Fig. S4). In addition, *O*-glycans that were degraded by both bacteria in monoculture, were also degraded in the co-culture (Fig. 5, Am, Rt, AmRt, 0–6 h, e.g. *m/z* 1114.4 and *m/z* 1155.4). Degradation of the different core structures by the co-culture *A. muciniphila*/*R. torques* illustrated a similar pattern compared to monoculture *R. torques* and *A. muciniphila* suggesting influence of both bacteria on *O*-glycan degradation (Fig. 6C and D, AmRt, 0–24 h, AmRt 9 h relative abundance core 2 decreased, AmRt 24 h relative abundance core 2 increased). To note, considering that the relative abundance of *A. muciniphila* was higher compared to *R. torques* after 12 h (ratio 70:30) and 24 h (ratio 80:20) in the co-culture *A. muciniphila*/*R. torques*, the low abundance of *R. torques* is sufficient for efficient *O*-glycan degradation. Since both bacteria are mucin glycan degradation specialists and therefore their genome is specialized towards mucin degradation (Geerlings et al. 2018, Kostopoulos et al. 2021, Schaus et al. 2024), the presence of both bacteria after 24 h in co-culture on PGM could suggest that they can co-exist and find their own specific niche (Wang et al. 2013, Van Herreweghen et al. 2021, Schaus et al. 2024). However, the increase in abundance of *A. muciniphila* overtime could also indicate a competitive advantage over *R. torques* when grown together on PGM (SI Fig. S4, AmRt).

Co-culture *A. muciniphila*/*B. thetaiotaomicron* (AmBt) degraded fucosylated and non-fucosylated *O-*glycans within 24 h (Fig. 5, AmBt, 0–24 h, Fig. 6A and B, AmBt, 0–24 h, and SI Fig. S5). Core 2 *O*-glycans were preferentially degraded compared to other core structures as their relative abundance decreased overtime (Fig. 6C and D, AmBt, 0–24 h). Compared to monoculture *B. thetaiotaomicron*, increased bacterial growth (SI Figs S1 and S2) and glycan degradation (Fig. 5, Am, Bt, AmBt, 9–24 h) were observed by co-culture *A. muciniphila*/*B. thetaiotaomicron*. A minimal decrease was demonstrated in relative abundance of *B. thetaiotaomicron* over time in co-culture with *A. muciniphila* (SI Fig. S5, AmBt, 0–24 h, 14:86 after 12 h and 10:90 after 24 h). The steady presence of *A. muciniphila* in co-culture with *B. thetaiotaomicron* emphasised the robustness of *A. muciniphila* in a mucin-rich environment. Kostopoulos et al. (2021) indicated that *B. thetaiotaomicron*, in presence of *A. muciniphila*, increased the expression of its hydrolytic machinery to compete with *A. muciniphila* for mucin glycans (Kostopoulos et al. 2021). This could suggest that the presence of other bacteria or the released *O-*glycan products could function as a competitive trigger for *B. thetaiotaomicron* resulting in increased growth and *O-*glycan degradation of *B. thetaiotaomicron* co-cultures compared to *B. thetaiotaomicron* monoculture. Another option is cross-feeding by *B. thetaiotaomicron* using the released *O-*glycan products made available by *A. muciniphila*. Research performed by Schaus et al. (2024) indicated that *A. muciniphila* supported *B. thetaiotaomicron* in co-culture on PGM. Both competition and cooperation could, again, lead to a relative increase in abundance of *B. thetaiotaomicron*, which could be beneficial for human health as *B. thetaiotaomicron* supports growth of butyrate-producing bacteria and metabolizes sugars to provide nutrients to the host and other microbiota (Chia et al. 2020, Townsend et al. 2020, Zafar and Saier 2021).

Co-culture *R. torques*/*B. thetaiotaomicron* degraded all PGM *O-*glycans within 9–12 h (Fig. 5, RtBt, 0–12 h, e.g. *m/z* 733.3 GalNAcα1–3(Fucα1–2)Galβ1–3GalNAc/GalNAcα1–3(Fucα1–2)Galβ1–4GlcNAc and *m/z* 952.4 GlcNAcβ1–3Galβ1–4GlcNAcβ1–3Galβ1–3GalNAc and SI Fig. S6). Fucosylated *O*-glycans were preferentially degraded compared to non-fucosylated as illustrated by the decrease in ratio of fucosylated *O*-glycans overtime (Fig. 6A and B, RtBt, 0–24 h). The different core structures were all degraded by co-culture *R. torques*/*B. thetaiotaomicron* (Fig. 6C and D, RtBt, 0–24 h). Degradation of complex *O-*glycans after 6 h (Fig. 5, RtBt, 6 h, *m/z* > 1000, presumably by *R. torques*) was followed by degradation of less complex *O-*glycans (Fig. 5, RtBt, 9 h, *m/z* < 1000, presumably by both *R. torques* and *B. thetaiotaomicron*). Furthermore, complete degradation of small dimers and trimeric glycans, presumably by *B. thetaiotaomicron*, was observed [Fig. 5, Rt, Bt, RtBt, 0–24 h, not degraded within 24 h by monoculture *R. torques* (Fig. 3)]. This could suggest cross-feeding by *B. thetaiotaomicron* as it has easier access to degraded products or remaining glycans when in co-culture with *R. torques*. Such mechanism is supported by research performed by Schaus et al. (2024) where it was shown that *R. torques* can release products from PGM, making the remaining *O-*glycans better accessible for *B. thetaiotaomicron* (Schaus et al. 2024).

The co-culture of the three bacteria *A. muciniphila*/*R. torques/B. thetaiotaomicron* degraded all present *O-*glycans within 3–12 h (Fig. 5, AmRtBt and SI Fig. S7). Fucosylated *O*-glycans were preferentially degraded compared to non-fucosylated *O*-glycans (Fig. 6A and B, AmRtBt, 0–24 h and Fig. 5, e.g. *m/z* 895.4 and *m/z* 1089.4). All core structures were degraded by the co-culture *A. muciniphila*/*R. torques/B. thetaiotaomicron* (Fig. 6C and D, AmRtBt, 0–24 h). Furthermore, a preferential degradation towards larger *O-*glycans was seen (Fig. 5, AmRtBt, 3 h, *m/z* > 900) over small *O-*glycans (Fig. 5, AmRtBt, 3 h, *m/z* < 900). This related back to degradation preferences of the bacteria *A. muciniphila* and *R. torques* in monoculture. It should be noted that the apparent lower preference to degrade small *O-*glycans could be due to the production of these smaller glycans as the break-down products of longer *O-*glycans.

All co-cultures with *B. thetaiotaomicron* were able to degrade small *O-*glycans *m/z* 384.2, *m/z* 425.3, and *m/z* 587.3 to completion after 24 h, suggesting that the partly degraded structures are an easier substrate for *B. thetaiotaomicron* to degrade and utilize. The co-cultures containing *R. torques* showed fast degradation of fucosylated and larger mucin *O-*glycans (Fig. 5, AmRt, RtBt, AmRtBt, 0–3 h, e.g. *m/z* 895.4, *m/z* 1089.4, *m/z* 1520.5 and Fig. 6A and B, AmRt, RtBt, AmRtBt, 0–24 h), which was less pronounced by co-culture *A. muciniphila/B. thetaiotaomicron* (Fig. 5, AmBt and Fig. 6A and B, AmBt, 0–24 h). A low relative abundance of *R. torques* was observed in the co-cultures already after 12 h and a further decrease in relative abundance was observed after 24 h (SI Fig. S3). The qPCR data illustrated that the total bacterial abundance of *A. muciniphila/R. torques* decreased after 12 h suggesting all mucin glycan resources were utilized. For *A. muciniphila/B. thetaiotaomicron* and *R. torques*/*B. thetaiotaomicron* the total bacterial abundance still increased after 12 h suggesting that there were still mucin glycan resources left for the bacteria to grow on (more pronounced for *A. muciniphila* and *B. thetaiotaomicron* than *R. torques* as the relative abundance of *R. torques* decreased between 12 and 24 h). Therefore, it could be speculated that *R. torques* in relatively low abundance already has an impact on mucin glycan degradation by degradation of fucosylated and larger mucin *O-*glycans. Furthermore, other bacteria present might have an essential role in regulating abundance of *R. torques*, which is supported by these results showing a decrease in relative abundance of *R. torques* over time when grown on PGM in co-culture (SI Fig. S3). It could be hypothesised that the presence of *R. torques* is essential in mucin glycan degradation and utilization but specifically in low abundance. In literature, a relatively high abundance of *R. torques* in humans has been correlated with undesired mucus layer break-down and gastrointestinal disease (Tailford et al. 2015, Lloyd-Price et al. 2019, Schaus et al. 2024).

The MDSC showed complete degradation of all present *O*-glycans within 24 h (Fig. 5, MDSC, 0–24 h). This indicates that the bacteria present in the MDSC were capable of producing all enzymes necessary to degrade the PGM *O-*glycans completely, highlighting the effectivity of mucin glycan degraders and cross-feeders for complete degradation of mucin *O-*glycans. It should be taken into account that, while the total amount of bacteria is similar between all inocula at 0 h, the absolute number of each individual bacterium in the MDSC inoculum was lower compared to that of the tested monocultures and co-cultures. The MDSC preferentially degraded fucosylated *O*-glycans as their relative abundance decreased compared to non-fucosylated *O*-glycans (Fig. 5, MDSC, 0–9 h, e.g. *m/z* 895.4, 1089.4, and *m/z* 1301.5 and Fig. 6A and B, MDSC, 0–24 h). The MDSC degraded all core structures and no specific preference was observed as there was little change in the ratios between the core structure (Fig. 5, MDSC, 0–9 h, e.g. isomers *m/z* 895.4 and *m/z* 952.4 and Fig. 6C and D, MDSC, 0–24 h). Since there is a wide variety of bacteria present in the MDSC, it could be speculated that there is minimal preference for core structure as a large variety of enzymes is present (*A. muciniphila, R. torques*, and *B. thetaiotaomicron* together can degrade all structures).

Even though it cannot be fully excluded that some *O*-glycan structures identified at 0 h could emerge by chemical degradation resulting from the manufactural preparation of PGM or the release of PGM *O*-glycans, the degradation behaviour by the tested bacteria was based on comparison of the remaining *O*-glycan structures overtime (6, 9, 12, and 24 h compared to 0 h). Therefore, the observed distinct degradation will mostly be the result from enzymatic degradation by the bacteria. The obtained results support that co-cultures or a bacterial community can more efficiently and completely degrade mucin *O-*glycans compared to monocultures. This is likely due to ecological interactions between bacteria through competition for available and preferred substrate (Kostopoulos et al. 2021), mutualistic or commensal interactions (species benefitting from mucin glycan degradation by other species) (Belzer et al. 2017), and through the availability of a broader range of enzymes involved in mucin degradation (Glover et al. 2022). This could suggest opportunities to steer the microbiome in a specific direction beneficial for human health based on individual circumstances and knowledge on present mucin glycan-degrading bacteria (Qingbo et al. 2024). This is also reflected by the degradation of different blood group antigens based on mucosal composition (Hoskins et al. 1985b, Jöud et al. 2018) and differences in abundance of specific mucin glycan degraders in the three commonly identified human GIT enterotypes (*Bacteroides, Prevotella*, and *Ruminococcus*) (Cheng and Ning 2019, Alemao et al. 2021, Yuan et al. 2022).

### A bacterial community is necessary to degrade all different *N*-glycan structures present in PGM

**Figure 7.**
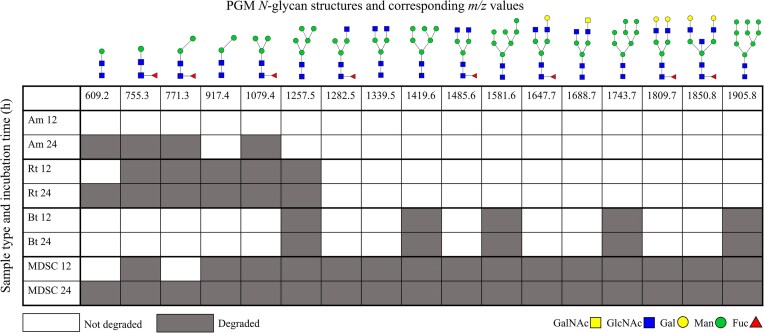
Heatmap of the identified *N-*glycans resulting from PGM incubation with *A. muciniphila* (Am), *R. torques* (Rt), *B. thetaiotaomicron* (Bt), and the MDSC, demonstrating the degradation rates of *N-*glycans during 24 h incubation. The intensity values (obtained from the peak height of the MALDI-TOF mass spectra, average of three replicates) of the *N-*glycans at 0 h were set to 100% and the intensity values of 12 and 24 h are shown in relation to this. The left *y*-axis displays bacterial cultures and sampling time (h). The top *x*-axis displays the *m/z* values of the corresponding [M+Na]^+^  *N-*glycan structures.

Besides *O-*glycans, PGM also contains *N-*glycans, even though present in relatively low levels (Luis and Hansson 2023). Analysis of PGM demonstrated mostly high-mannose type *N-*glycans, with up to eight mannosyl residues attached, as well as complex type *N-*glycans consisting of Galβ1–3GlcNAc and GalNAcβ1–4GlcNAc attached to the *N-*glycan core (Fig. 7). Furthermore, multiple fucosylated *N-*glycans were identified. Sialylated *N-*glycans [poorly detected using MALDI-TOF-MS (Van Der Post et al. 2014, Gallego et al. 2023)] are not shown in the results as only a very low abundance was present (confirmed by PGM-LC-MS/MS analysis, data not shown).

*A. muciniphila* and *R. torques* only partially degraded the smaller *N-*glycan structures within 24 h (Fig. 7, Am, Rt, 12–24 h and SI Figs S13 and S14 show the MALDI-TOF mass spectra). *B. thetaiotaomicron* degraded the high-mannose structures but limited degradation of all other structures was seen within 24 h (Fig. 7, Bt, 12–24 h, e.g. *m/z* 1419.6, *m/z* 1581.6, and *m/z* 1743.7 and SI Fig. S15). Mannosidases are required for breaking down high-mannose type *N-*glycans (Robb et al. 2017, Hobbs et al. 2018, Higgins et al. 2021, Crouch 2023). These enzymes are present in the genome of *B. thetaiotaomicron* (glycan-degrading generalist), but are lacking in the genomes of *A. muciniphila* and *R. torques* (mucin glycan-degrading specialists), explaining the limited *N-*glycan degradation by *A. muciniphila* and *R. torques* and suggesting a potentially unique niche for *B. thetaiotaomicron* during growth in a community on mucin glycans. Furthermore, the removal of Fuc was proposed via similar enzymes applicable for cleavage of fucosylated *O-*glycans. However, our results demonstrated incomplete Fuc release in *N-*glycans by *A. muciniphila, R. torques*, and *B. thetaiotaomicron* after 24 h suggesting different enzyme efficiency or specificity or sterical hindrance (Fig. 7, Am, Rt, Bt, 12–24 h). The MDSC completely degraded all identified *N-*glycan structures within 24 h (Fig. 7, MDSC, 12–24 h and SI Fig. S16). The complete degradation of PGM *N-*glycans suggest that the selected bacterial community produced all the necessary enzymes for complete PGM *N-*glycan degradation. This supports that bacterial interaction is necessary for efficient and complete mucin glycan degradation.

### Mucin glycan-degrading bacteria grown on PGM exhibit distinct metabolite patterns

**Figure 8.**
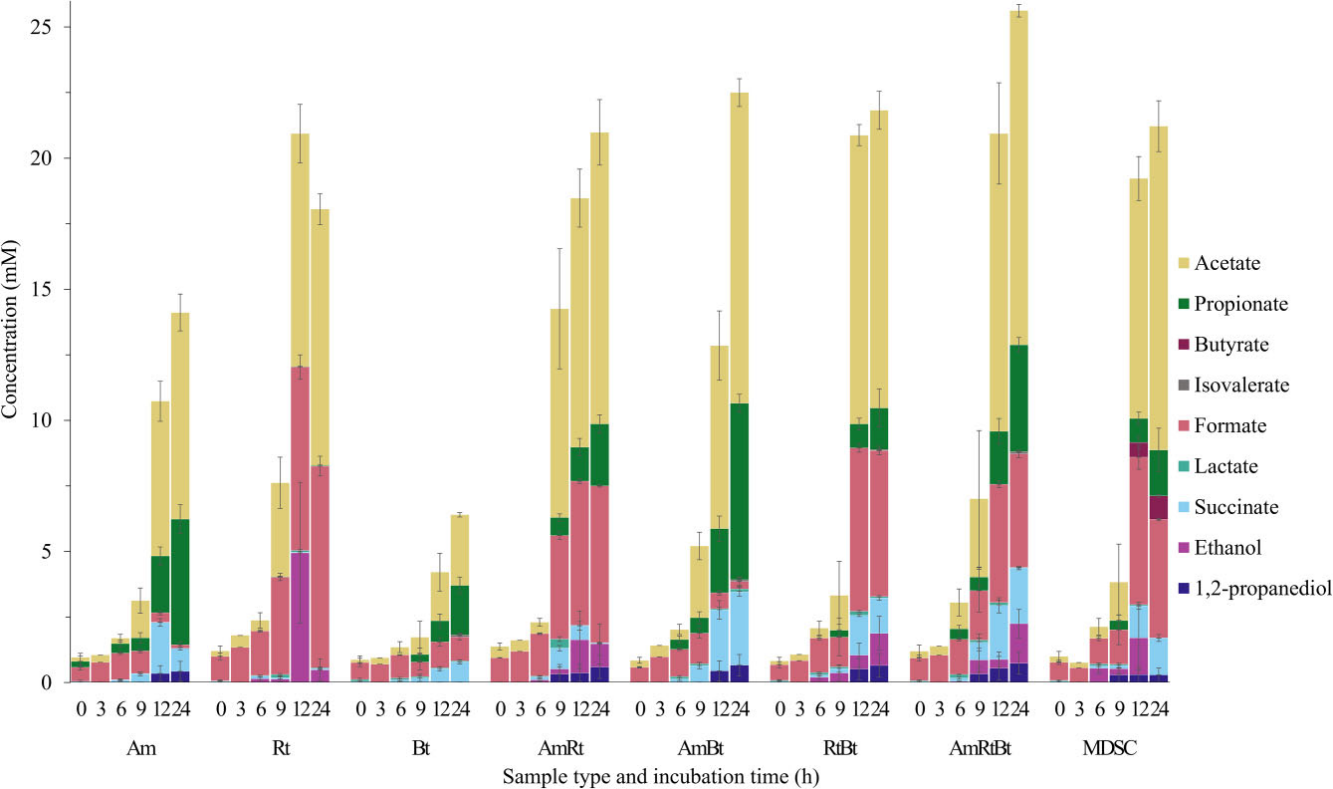
Metabolite formation during 24 h incubation of PGM by *A. muciniphila* (Am), *R. torques* (Rt), *B. thetaiotaomicron* (Bt), co-cultures (AmRt, AmBt, RtBt, AmRtBt), and an MDSC. The *x*-axis indicates the different cultures and the sampling time (h) and the *y*-axis depicts the concentration of total produced metabolites in mM (values shown are average of three replicates).

Metabolite production upon incubation with PGM was culture specific and different metabolite production profiles were observed for the monocultures, co-cultures, and the MDSC (Fig. 8 and SI Figs S17–S19 show calibration curves and example chromatograms). *A. muciniphila* produced formate, acetate, propionate, and succinate, showing especially an increase in the concentration of propionate between 9 and 24 h (Fig. 8). Decrease in formate, a by-product of anaerobic fermentation, and increase in acetate between 9 and 24 h is in accordance with literature and indicates that *A. muciniphila* can utilize formate for acetate production (Morrison and Preston 2016, Venegas et al. 2019). The increase in acetate and propionate between 9 and 24 h also confirms that *A. muciniphila* can utilize mucin glycans for production of these metabolites (Fig. 8) (Derrien et al. 2004, Rodrigues et al. 2022). *R. torques* produced acetate and formate in equal abundance between 9 and 24 h (Fig. 8). In addition, EtOH was formed as intermediate product at 12 h and had been almost utilized at 24 h. *B. thetaiotaomicron* produced the lowest concentration of metabolites after 24 h compared to all tested cultures, which relates to the limited growth of *B. thetaiotaomicron* on PGM (SI Figs S1 and S2). *B. thetaiotaomicron* produced acetate, formate, propionate, succinate, and isovalerate, the latter three increasing in concentration between 9 and 24 h (Fig. 8). Succinate serves as a circulating metabolite involved in cellular nutrient metabolism and it is an essential intermediate substrate for propionate production by certain bacteria, including *B. thetaiotaomicron* (Macy et al. 1978, Morrison and Preston 2016).

The co-cultures produced combinations of the metabolites as formed by the monocultures in distinct ratios. This could be correlated with the relative abundance of the bacteria in co-culture (SI Fig. S3) and the proportions of the produced metabolites indicate glycan utilization by all three bacteria. For example, production of propionate is associated with *A. muciniphila* and *B. thetaiotaomicron*, lactate with *B. thetaiotaomicron*, and production of EtOH and a high concentration of formate is correlated to the presence of *R. torques*. As expected, the MDSC was the only culture that produced butyrate. Butyrate, specifically associated with health benefits, can be produced by the cross-feeding (butyrate producing) bacteria present, such as *F. duncaniae*, using available acetate and lactate (Morrison and Preston 2016, Hodgkinson et al. 2023, Chollet et al. 2024).

Type and level of the various metabolites show that the monocultures, the co-cultures, and the MDSC all produced unique profiles of metabolites in distinct ratios (Fig. 8, 9–24 h), indicating the use of different metabolic pathways by the bacteria upon incubation on PGM. This could be due to variable availability of sugars from the PGM glycan chains that specific microbes can access. The fermentation pathways, depend on the released sugars, will lead to different produced metabolite profiles. The production of total amount of metabolites differed among the tested cultures; *B. thetaiotaomicron* produced the lowest amount and the co-cultures produced increased metabolite levels compared to the monocultures (Fig. 8). This correlated with the observed OD600 and qPCR data (SI Figs S1 and S2). It should be taken into account that less bacterial growth and glycan degradation are directly related to less metabolite production as well. Nonetheless, these results support the importance of a diverse microbiota for effective and efficient saccharolytic fermentation of mucin glycans in the colon (Morrison and Preston 2016).

## Conclusion

The mucin glycan-degrading bacteria *A. muciniphila, R. torques*, and *B. thetaiotaomicron* in monoculture, co-culture, and as part of a synthetic community employ different strategies for PGM *O-*glycan and *N-*glycan degradation and utilization. Consequently, it can be speculated that the abundance of specific bacteria in the human GIT influences mucin glycan degradation as well as the microbiome composition. This knowledge is an important first step in understanding mucin glycan driven host–microbe interactions. Future research could further explore enzyme production by mucin-degrading bacteria regarding cleavage of glycan linkages, looking into the *O*- and *N*-glycan degradation potential of other known mucin glycan-degrading bacteria, and exploring more human-like mucin sources to better understand mucin glycan driven host–microbe interactions occurring in the human GIT.

## Supplementary Material

fiaf066_Supplemental_File

## Data Availability

Data will be made available on request.
