# Supplementary material for: Distinct in vitro utilization and degradation of porcine gastric mucin glycans by human intestinal bacteria
Source: FEMS Microbiol Ecol. 2025 Jun 23;101(8):fiaf066. doi: 10.1093/femsec/fiaf066 (PMC12258148; doi:10.1093/femsec/fiaf066)
Supplement: fiaf066_Supplemental_File [file fiaf066_supplemental_file.docx]

**Supporting information**

**Distinct *in vitro* utilisation and degradation of porcine gastric mucin glycans by human intestinal bacteria**

Carol de Ram^a‡^, Maryse D. Berkhout^b‡^, Carolina O. Pandeirada^a^, Jean-Paul Vincken^a^, Guido J. E. J. Hooiveld^c^, Clara Belzer^b°^, Henk A. Schols^a*°^

^a^ *Laboratory of Food Chemistry, Wageningen University & Research, Wageningen, the Netherlands*

^b^ *Laboratory of Microbiology, Wageningen University & Research, Wageningen, the Netherlands*

*^c^ Division of Human Nutrition and Health, Wageningen University & Research, Wageningen, the Netherlands*

*Corresponding author

*E-mail address:* [henk.schols@wur.nl](mailto:henk.schols@wur.nl) (H.A. Schols)

‡These authors contributed equally to this work

°These authors also contributed equally to this work

**Table of contents**

[Figure S1. Bacterial growth curves measured by OD600 measurement of *A. muciniphila* (Am), *R. torques* (Rt), *B. thetaiotaomicron* (Bt), co-cultures (Am/Rt, Am/Bt, Rt/Bt, Am/Rt/Bt), and the mucin-degrading synthetic community (MDSC) incubated on porcine gastric mucin (PGM) during 24 h. The OD600 values (three replicates) used for the graph were measured after 0, 6, 9, 12, and 24 h. 5](#_Toc198888191)

[Figure S2. Total bacterial abundance of *A. muciniphila* (Am), *R. torques* (Rt), *B. thetaiotaomicron* (Bt), co-cultures (Am/Rt, Am/Bt, Rt/Bt, Am/Rt/Bt), and the the mucin-degrading synthetic community (MDSC) upon incubation on PGM for 12 and 24 h was determined by qPCR (three replicates). The x-axis indicates the samples at the different timepoints (12 and 24 h) and the y-axis shows the average 16S copies per mL culture (16S rRNA gene copy number/uL in Log scale). 6](#_Toc198888192)

[Figure S3. Overview of bacterial composition of monocultures *A. muciniphila* (Am), *R. torques* (Rt), and *B. thetaiotaomicron* (Bt), co-cultures Am/Rt, Am/Bt, Rt/Bt, and Am/Rt/Bt, and the mucin-degrading synthetic community (MDSC) upon incubation on PGM for 12 and 24 h. The composition is based on measured relative abundance (three replicates) by 16S rRNA gene amplicon sequencing (protocol by Shetty et al (2022) ^1^ and corrected for number of 16S rRNA gene copies (Table 1 ^2^). 7](#_Toc198888193)

[Figure S4. PGC-LC-MS chromatograms demonstrating the *O-*glycan patterns remaining on the protein backbone after incubation of PGM with co-culture *A. muciniphila/R. torques* during 24 h. *O-*glycans were released prior to analysis. Structures of *O-*glycans are based on MS/MS fragmentation data. IS MP5: internal standard maltopentaose DP5. *Peaks could not be assigned to PGM *O-*glycans based on *m/z* and MS/MS fragmentation data. **Structure is a suggestion based on *m/z* and MS/MS fragmentation pattern. ***Structure is suspected to have emerged from chemical degradation. 8](#_Toc198888194)

[Figure S5. PGC-LC-MS chromatograms demonstrating the *O-*glycan patterns remaining on the protein backbone after incubation of PGM with co-culture *A. muciniphila/B. thetaiotaomicron* during 24 h. *O-*glycans were released prior to analysis. Structures of *O-*glycans are based on MS/MS fragmentation data. IS MP5: internal standard maltopentaose DP5. *Peaks could not be assigned to PGM *O-*glycans based on *m/z* and MS/MS fragmentation data. **Structure is a suggestion based on *m/z* and MS/MS fragmentation pattern. ***Structure is suspected to have emerged from chemical degradation. 9](#_Toc198888195)

[Figure S6. PGC-LC-MS chromatograms demonstrating the *O-*glycan patterns remaining on the protein backbone after incubation of PGM with co-culture *R. torques/B. thetaiotaomicron* during 24 h. *O-*glycans were released prior to analysis. Structures of *O-*glycans are based on MS/MS fragmentation data. IS MP5: internal standard maltopentaose DP5. *Peaks could not be assigned to PGM *O-*glycans based on *m/z* and MS/MS fragmentation data. **Structure is a suggestion based on *m/z* and MS/MS fragmentation pattern. ***Structure is suspected to have emerged from chemical degradation. 10](#_Toc198888196)

[Figure S7. PGC-LC-MS chromatograms demonstrating the *O-*glycan patterns remaining on the protein backbone after incubation of PGM with co-culture *A. muciniphila*/*R. torques/B. thetaiotaomicron* during 24 h. *O-*glycans were released prior to analysis. Structures of *O-*glycans are based on MS/MS fragmentation data. IS MP5: internal standard maltopentaose DP5. *Peaks could not be assigned to PGM *O-*glycans based on *m/z* and MS/MS fragmentation data. **Structure is a suggestion based on *m/z* and MS/MS fragmentation pattern. ***Structure is suspected to have emerged from chemical degradation. 11](#_Toc198888197)

[Figure S8. PGC-LC-MS chromatograms demonstrating the *O-*glycan patterns remaining on the protein backbone after incubation of PGM with the MDSC during 24 h. *O-*glycans were released prior to analysis. Structures of *O-*glycans are based on MS/MS fragmentation data. IS MP5: internal standard maltopentaose DP5. *Peaks could not be assigned to PGM *O-*glycans based on *m/z* and MS/MS fragmentation data. **Structure is a suggestion based on *m/z* and MS/MS fragmentation pattern. ***Structure is suspected to have emerged from chemical degradation. 12](#_Toc198888198)

[Figure S9. Heatmap of the foremost abundant mucin *O-*glycans resulting from replicate R1 PGM incubation with *A. muciniphila* (Am), *R. torques* (Rt), *B. thetaiotaomicron* (Bt), co-cultures (AmRt, AmBt, RtBt, AmRtBt), and the mucin-degrading synthetic community (MDSC), demonstrating the degradation rates of *O-*glycans during 24 h incubation. The intensity values (obtained from the peak areas) of the *O-*glycans at 0 h were set to 100% and the intensity values of 3 – 24 h are shown relative to the values at 0 h. The left y-axis indicates the bacterial cultures and the sampling time (h). The top x-axis displays the *m/z* values of the corresponding [M-H]^-^ *O-*glycan structures. *O-*glycan structures are based on acquired fragmentation data (PGC-LC-MS/MS). Sialylated *O-*glycan structures are not shown due to their low abundance and complete degradation within 3 - 6 h by each culture. 13](#_Toc198888199)

[Figure S10. Heatmap of the foremost abundant mucin *O-*glycans resulting from replicate R2 PGM incubation with *A. muciniphila* (Am), *R. torques* (Rt), *B. thetaiotaomicron* (Bt), co-cultures (AmRt, AmBt, RtBt, AmRtBt), and the mucin-degrading synthetic community (MDSC), demonstrating the degradation rates of *O-*glycans during 24 h incubation. The intensity values (obtained from the peak areas) of the *O-*glycans at 0 h were set to 100% and the intensity values of 3 – 24 h are shown relative to the values at 0 h. The left y-axis indicates the bacterial cultures and the sampling time (h). The top x-axis displays the *m/z* values of the corresponding [M-H]^-^ *O-*glycan structures. *O-*glycan structures are based on acquired fragmentation data (PGC-LC-MS/MS). Sialylated *O-*glycan structures are not shown due to their low abundance and complete degradation within 3 - 6 h by each culture. 14](#_Toc198888200)

[Figure S11. Heatmap of the foremost abundant mucin *O-*glycans resulting from replicate R3 PGM incubation with *A. muciniphila* (Am), *R. torques* (Rt), *B. thetaiotaomicron* (Bt), co-cultures (AmRt, AmBt, RtBt, AmRtBt), and the mucin-degrading synthetic community (MDSC), demonstrating the degradation rates of *O-*glycans during 6 - 24 h incubation (insufficient sample was available from 0 h and 3 h). The intensity values (obtained from the peak areas) of the *O-*glycans at 6 h were set to 100% and the intensity values of 9 – 24 h are shown relative to the values at 0 h. The left y-axis indicates the bacterial cultures and the sampling time (h). The top x-axis displays the *m/z* values of the corresponding [M-H]^-^ *O-*glycan structures. *O-*glycan structures are based on acquired fragmentation data (PGC-LC-MS/MS). Sialylated *O-*glycan structures are not shown due to their low abundance and complete degradation within 6 h by each culture as shown. 15](#_Toc198888201)

[Figure S12. PGC-LC-MS/MS fragmentation spectra demonstrating the identification of the present *O-*glycans remaining on the protein backbone after incubation of PGM with co-culture monocultures *A. muciniphila*, *R. torques*, *B. thetaiotaomicron*, co-cultures thereof, and the MDSC during 24 h. *O-*glycans were released prior to analysis. 24](#_Toc198888202)

[Figure S13. MALDI-TOF mass spectra demonstrating the *N-*glycan patterns remaining on the protein backbone after incubation of PGM with *A. muciniphila* during 24 h. *N-*glycans were enzymatically released prior to analysis. The x-axis displays the *m/z* values of the corresponding [M+Na]^+^ *N-*glycan structures. IS MP5: internal standard maltopentaose DP5. 25](#_Toc198888203)

[Figure S14. MALDI-TOF mass spectra demonstrating the *N-*glycan patterns remaining on the protein backbone after incubation of PGM with *R. torques* during 24 h. *N-*glycans were enzymatically released prior to analysis. The x-axis displays the *m/z* values of the corresponding [M+Na]^+^ *N-*glycan structures. IS MP5: internal standard maltopentaose DP5. 26](#_Toc198888204)

[Figure S15. MALDI-TOF mass spectra demonstrating the *N-*glycan patterns remaining on the protein backbone after incubation of PGM with *B. thetaiotaomicron* during 24 h. *N-*glycans were enzymatically released prior to analysis. The x-axis displays the *m/z* values of the corresponding [M+Na]^+^ *N-*glycan structures. IS MP5: internal standard maltopentaose DP5. 27](#_Toc198888205)

[Figure S16. MALDI-TOF mass spectra demonstrating the *N-*glycan patterns remaining on the protein backbone after incubation of PGM with the MDSC during 24 h. *N-*glycans were enzymatically released prior to analysis. The x-axis displays the *m/z* values of the corresponding [M+Na]^+^ *N-*glycan structures. IS MP5: internal standard maltopentaose DP5. 28](#_Toc198888206)

[Figure S17. Calibration curves used for quantification of metabolites produced by the bacteria (Am, Rt, Bt, co-cultures, and the MDSC) when grown on PGM over time (0 – 24 h). 29](#_Toc198888207)

[Figure S18. Standards used for calibration measured using HPLC. Two standards were used as there was overlap in the peaks originating from butyrate and ethanol. Therefore, 1 standard contained succinate, lactate, formate, acetate, propionate, butyrate, and iso-valerate (0.1 – 4.0 mg/mL; top figure) and the other standard contained 1,2-propanediol and ethanol (0.1 – 4.0 mg/mL; bottom figure). These standards were used for quantification of metabolites produced by the bacteria (Am, Rt, Bt, co-cultures, and the MDSC) when grown on PGM over time (0 – 24 h). 30](#_Toc198888208)

[Figure S19. Example chromatograms of the metabolite production by (A) *A. muciniphila*, (B) *R. torques*, and (C) *B. thetaiotaomicron* grown on PGM over 24 h. 31](#_Toc198888209)

[Table S1. Bactrial strains used and the associated number of 16S rRNA gene copies ^2^. 23](#_Toc195775971)


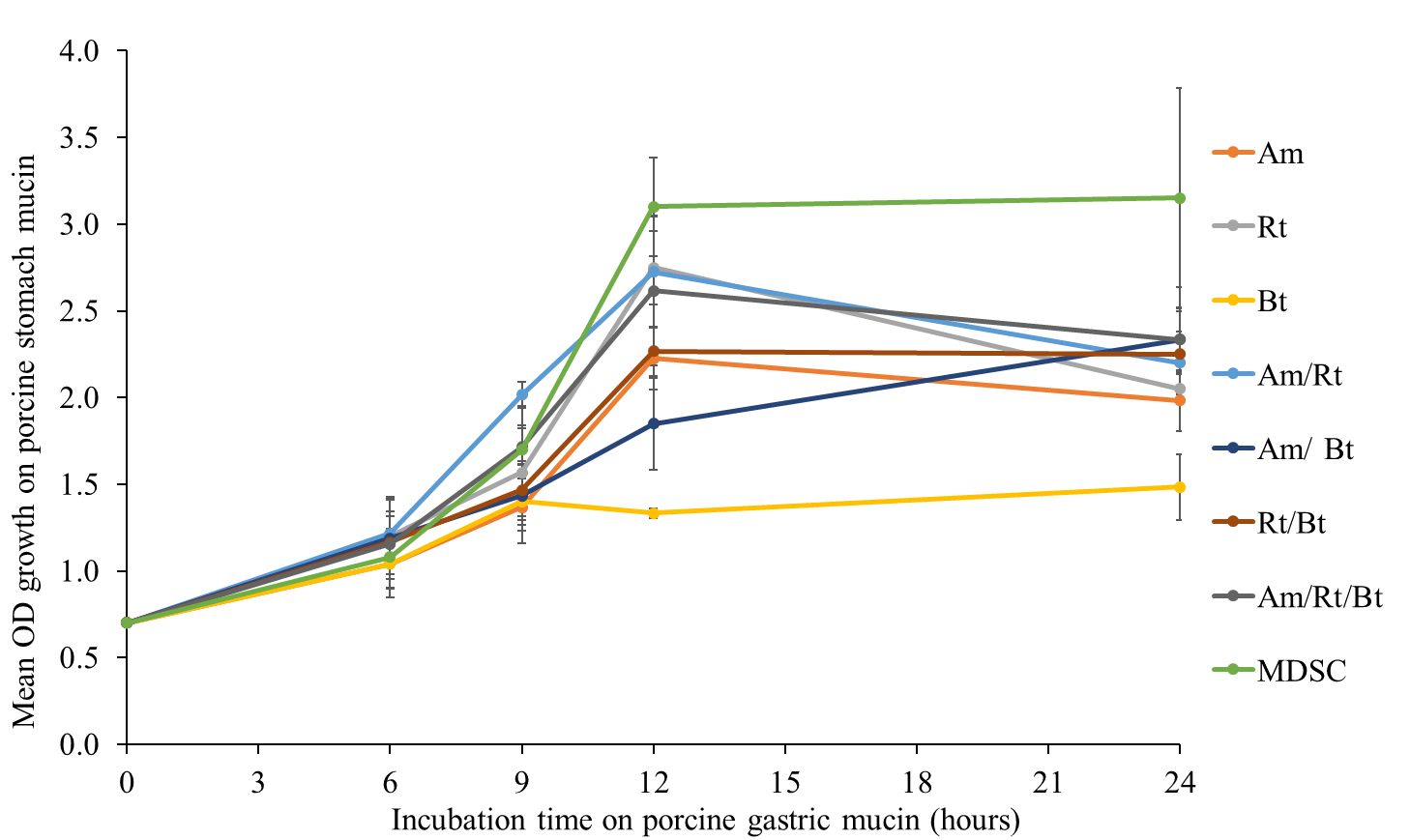


Figure S1. Bacterial growth curves measured by OD600 measurement of *A. muciniphila* (Am), *R. torques* (Rt), *B. thetaiotaomicron* (Bt), co-cultures (Am/Rt, Am/Bt, Rt/Bt, Am/Rt/Bt), and the mucin-degrading synthetic community (MDSC) incubated on porcine gastric mucin (PGM) during 24 h. The OD600 values (three replicates) used for the graph were measured after 0, 6, 9, 12, and 24 h.


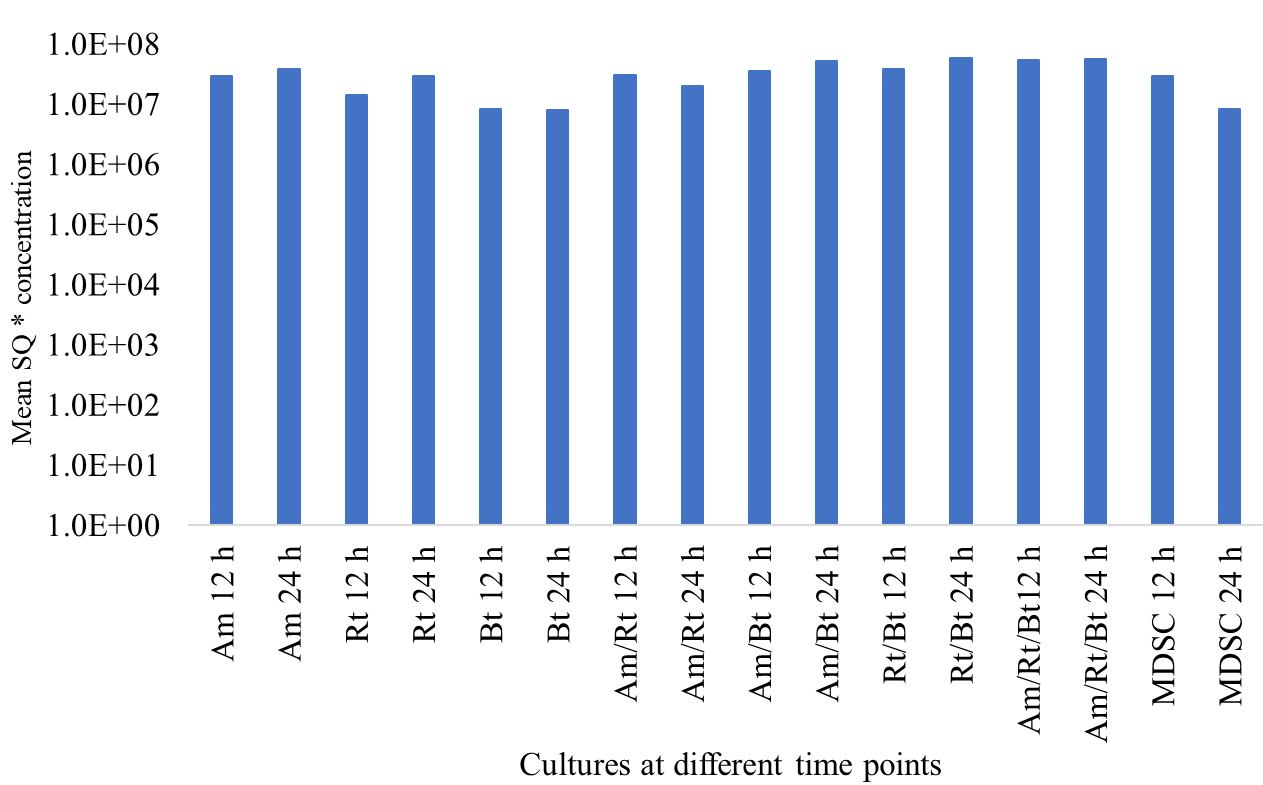


Figure S2. Total bacterial abundance of *A. muciniphila* (Am), *R. torques* (Rt), *B. thetaiotaomicron* (Bt), co-cultures (Am/Rt, Am/Bt, Rt/Bt, Am/Rt/Bt), and the the mucin-degrading synthetic community (MDSC) upon incubation on PGM for 12 and 24 h was determined by qPCR (three replicates). The x-axis indicates the samples at the different timepoints (12 and 24 h) and the y-axis shows the average 16S copies per mL culture (16S rRNA gene copy number/uL in Log scale).


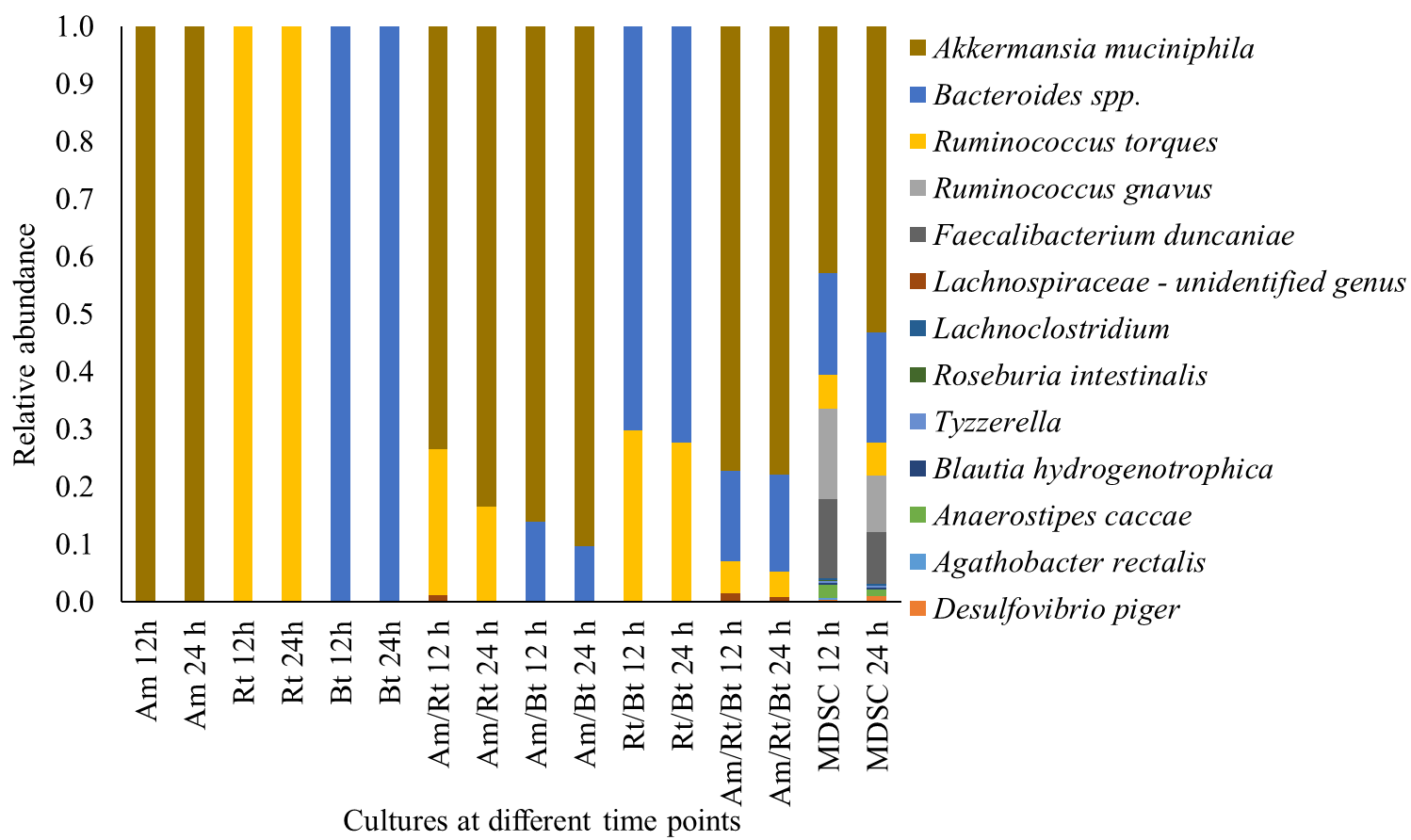


Figure S3. Overview of bacterial composition of monocultures *A. muciniphila* (Am), *R. torques* (Rt), and *B. thetaiotaomicron* (Bt), co-cultures Am/Rt, Am/Bt, Rt/Bt, and Am/Rt/Bt, and the mucin-degrading synthetic community (MDSC) upon incubation on PGM for 12 and 24 h. The composition is based on measured relative abundance (three replicates) by 16S rRNA gene amplicon sequencing (protocol by Shetty et al (2022) ^1^ and corrected for number of 16S rRNA gene copies (Table 1 ^2^).


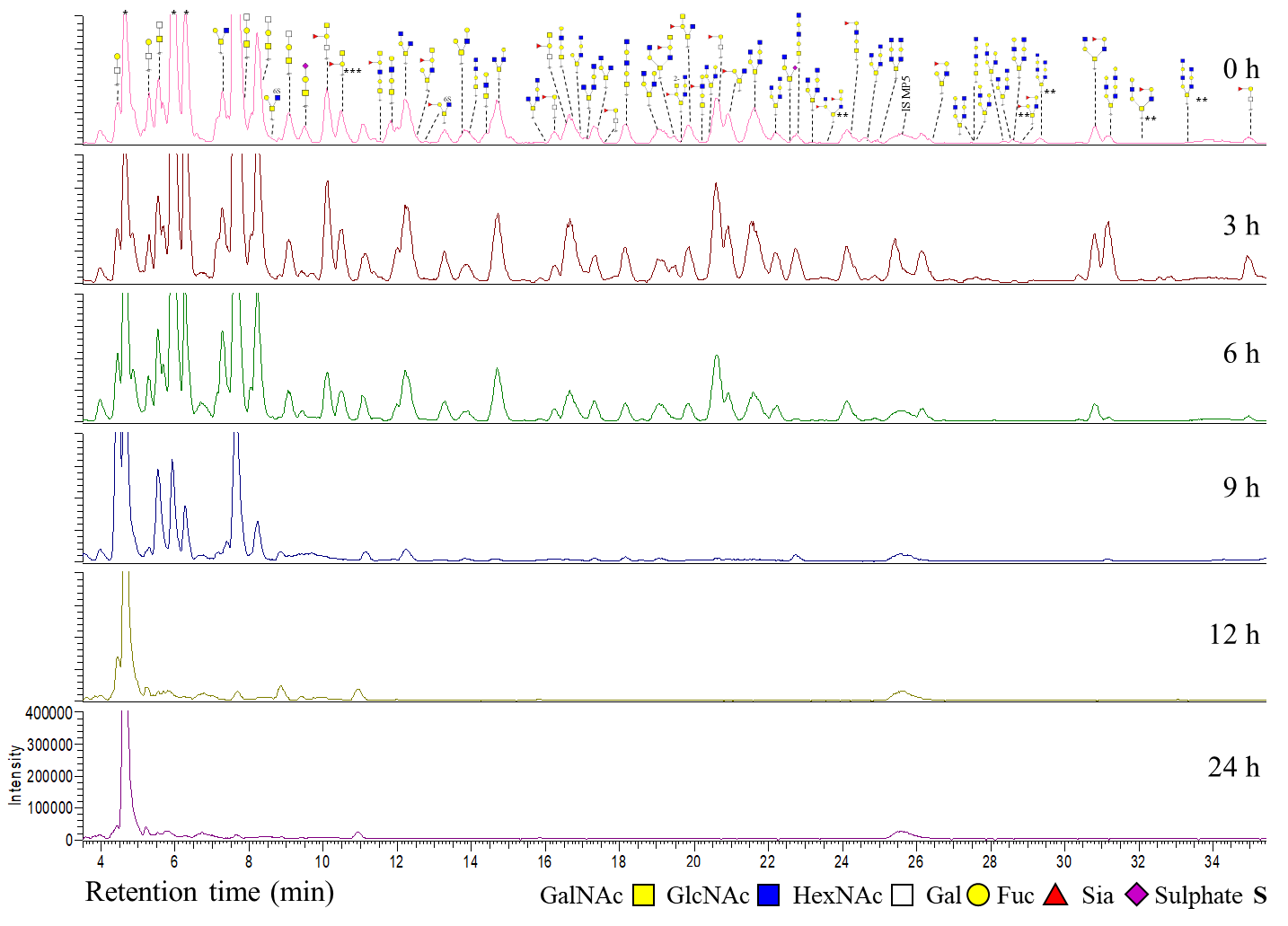


Figure S4. PGC-LC-MS chromatograms demonstrating the *O-*glycan patterns remaining on the protein backbone after incubation of PGM with co-culture *A. muciniphila/R. torques* during 24 h. *O-*glycans were released prior to analysis. Structures of *O-*glycans are based on MS/MS fragmentation data. IS MP5: internal standard maltopentaose DP5. *Peaks could not be assigned to PGM *O-*glycans based on *m/z* and MS/MS fragmentation data. **Structure is a suggestion based on *m/z* and MS/MS fragmentation pattern. ***Structure is suspected to have emerged from chemical degradation.

C


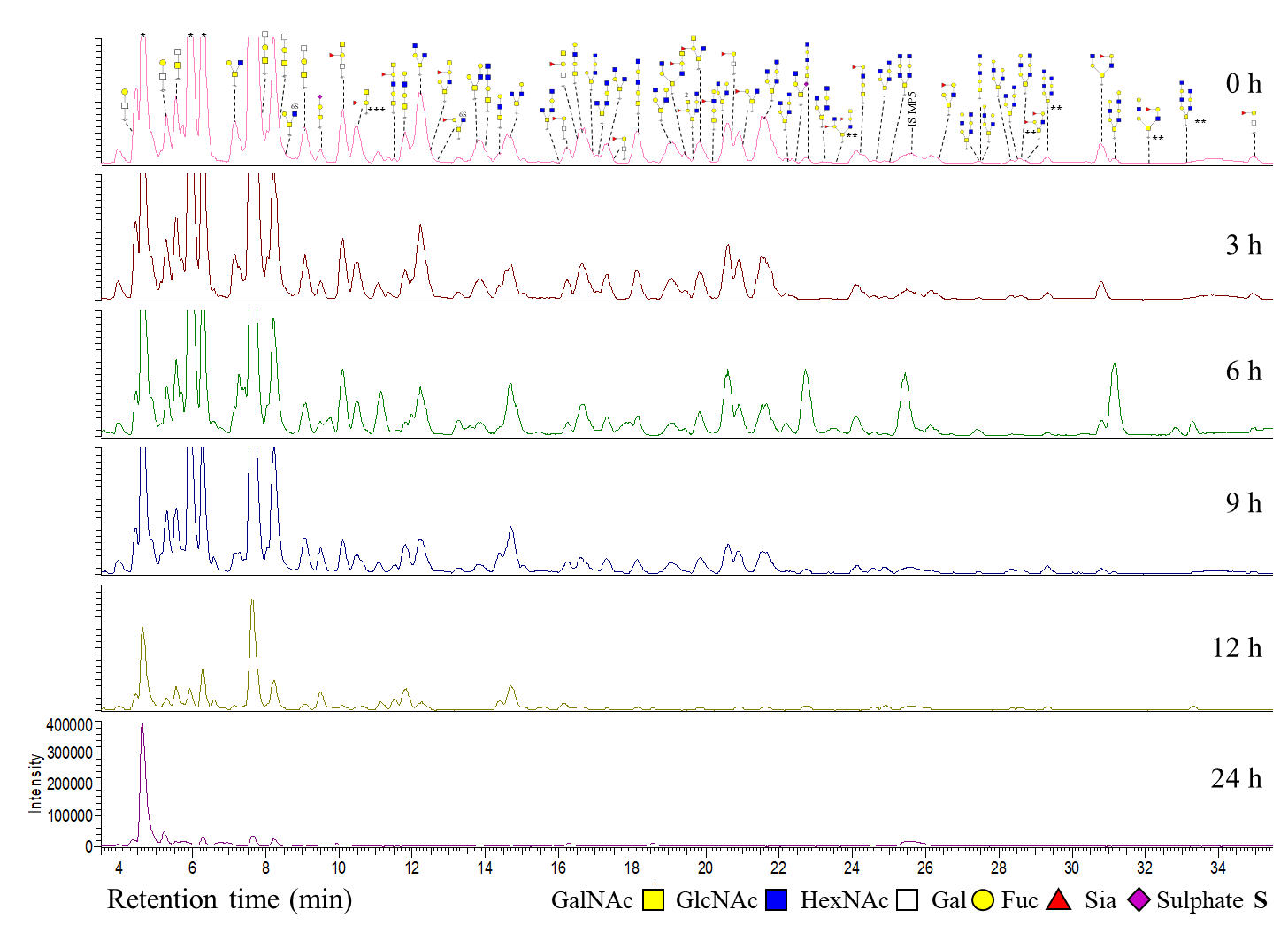


Figure S5. PGC-LC-MS chromatograms demonstrating the *O-*glycan patterns remaining on the protein backbone after incubation of PGM with co-culture *A. muciniphila/B. thetaiotaomicron* during 24 h. *O-*glycans were released prior to analysis. Structures of *O-*glycans are based on MS/MS fragmentation data. IS MP5: internal standard maltopentaose DP5. *Peaks could not be assigned to PGM *O-*glycans based on *m/z* and MS/MS fragmentation data. **Structure is a suggestion based on *m/z* and MS/MS fragmentation pattern. ***Structure is suspected to have emerged from chemical degradation.


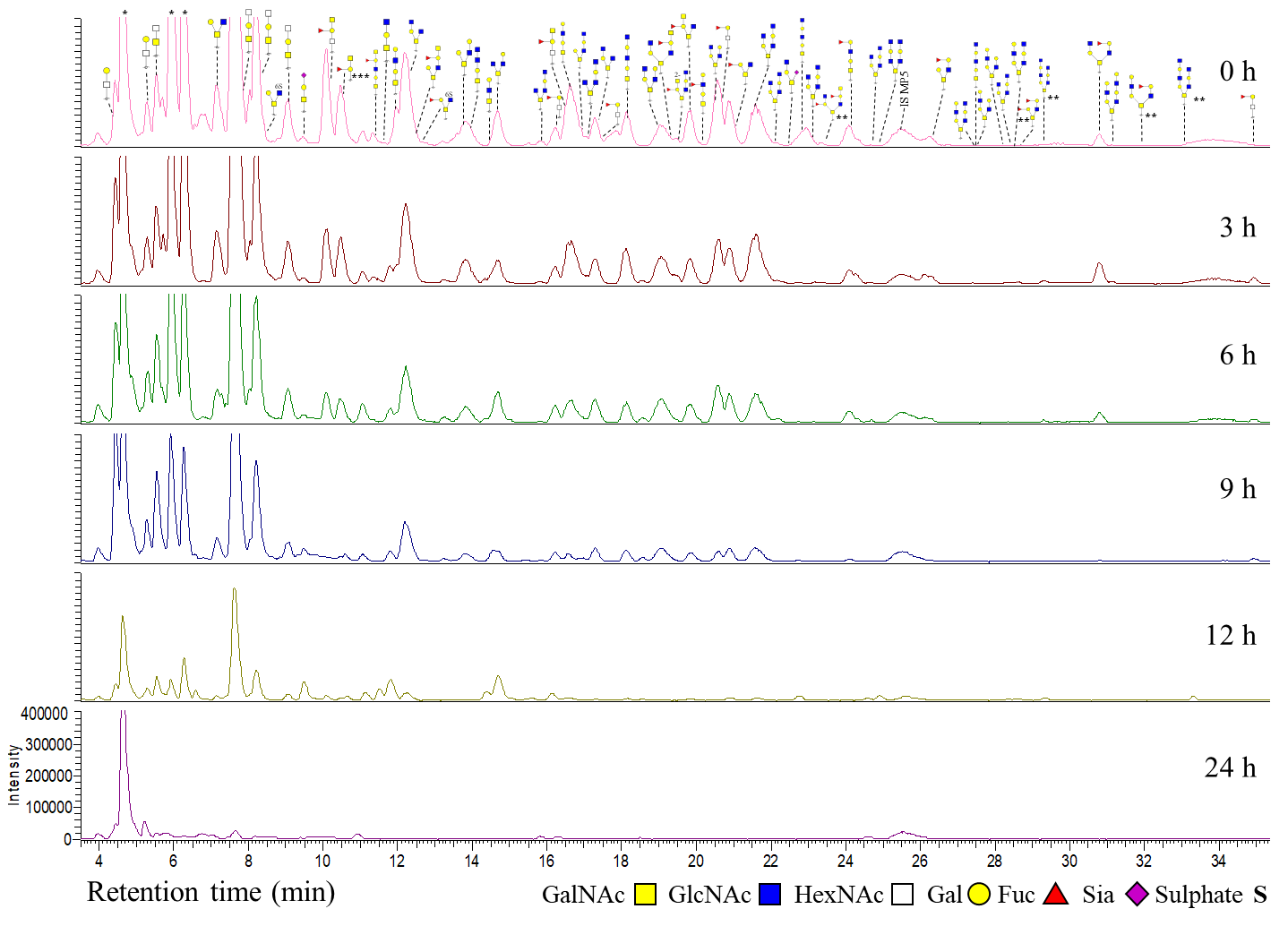


Figure S6. PGC-LC-MS chromatograms demonstrating the *O-*glycan patterns remaining on the protein backbone after incubation of PGM with co-culture *R. torques/B. thetaiotaomicron* during 24 h. *O-*glycans were released prior to analysis. Structures of *O-*glycans are based on MS/MS fragmentation data. IS MP5: internal standard maltopentaose DP5. *Peaks could not be assigned to PGM *O-*glycans based on *m/z* and MS/MS fragmentation data. **Structure is a suggestion based on *m/z* and MS/MS fragmentation pattern. ***Structure is suspected to have emerged from chemical degradation.


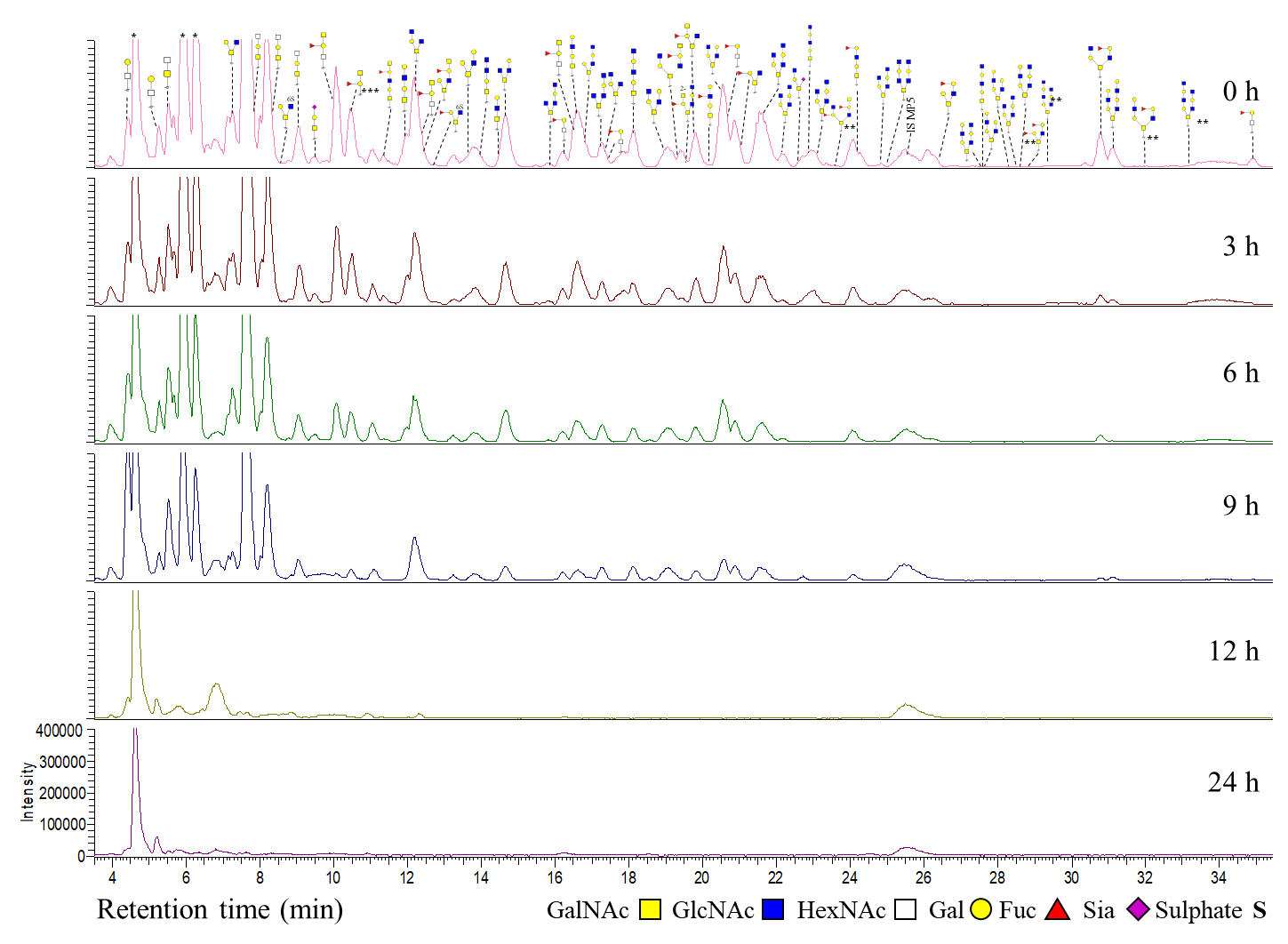


Figure S7. PGC-LC-MS chromatograms demonstrating the *O-*glycan patterns remaining on the protein backbone after incubation of PGM with co-culture *A. muciniphila*/*R. torques/B. thetaiotaomicron* during 24 h. *O-*glycans were released prior to analysis. Structures of *O-*glycans are based on MS/MS fragmentation data. IS MP5: internal standard maltopentaose DP5. *Peaks could not be assigned to PGM *O-*glycans based on *m/z* and MS/MS fragmentation data. **Structure is a suggestion based on *m/z* and MS/MS fragmentation pattern. ***Structure is suspected to have emerged from chemical degradation.


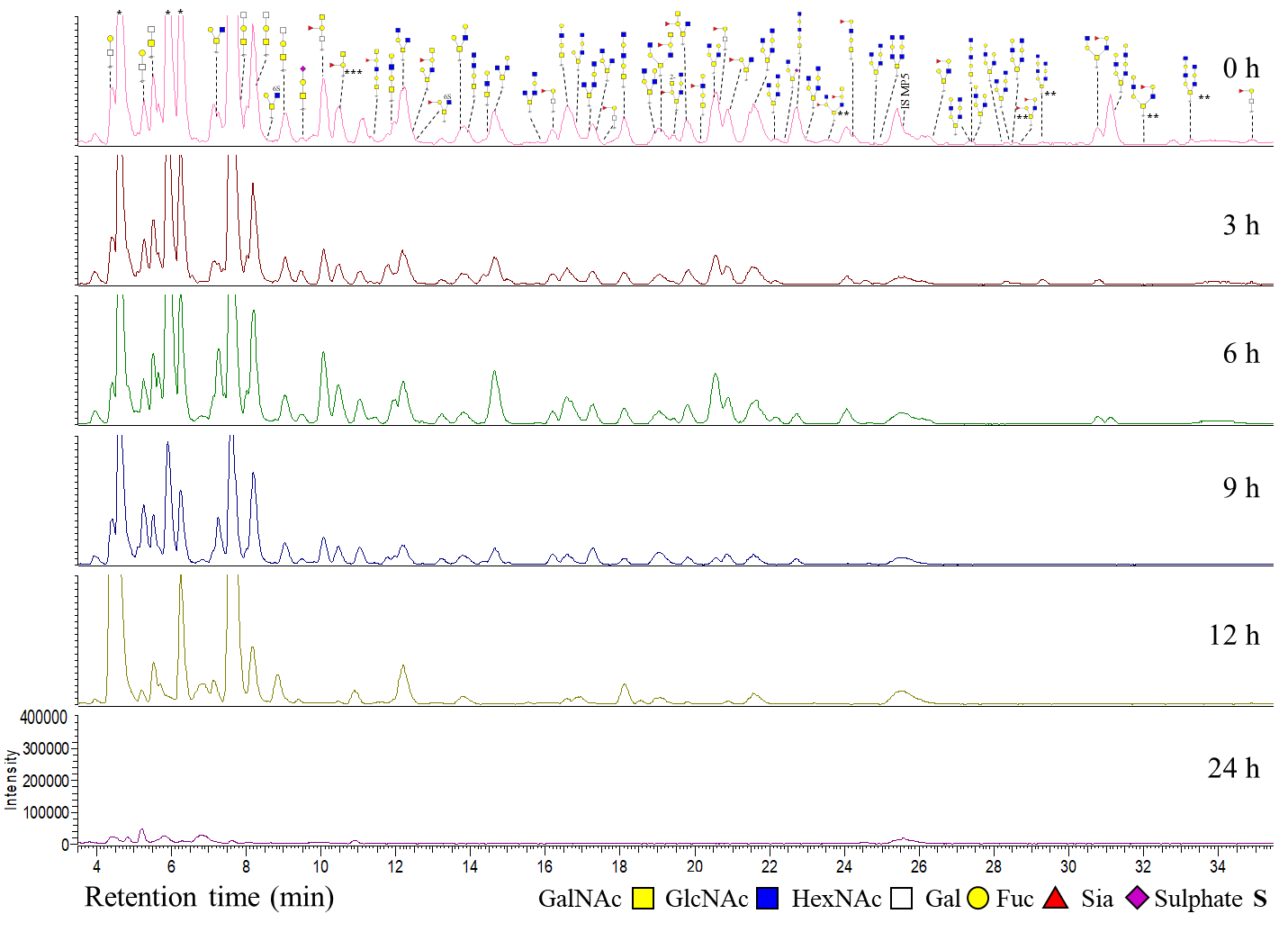


Figure S8. PGC-LC-MS chromatograms demonstrating the *O-*glycan patterns remaining on the protein backbone after incubation of PGM with the MDSC during 24 h. *O-*glycans were released prior to analysis. Structures of *O-*glycans are based on MS/MS fragmentation data. IS MP5: internal standard maltopentaose DP5. *Peaks could not be assigned to PGM *O-*glycans based on *m/z* and MS/MS fragmentation data. **Structure is a suggestion based on *m/z* and MS/MS fragmentation pattern. ***Structure is suspected to have emerged from chemical degradation.


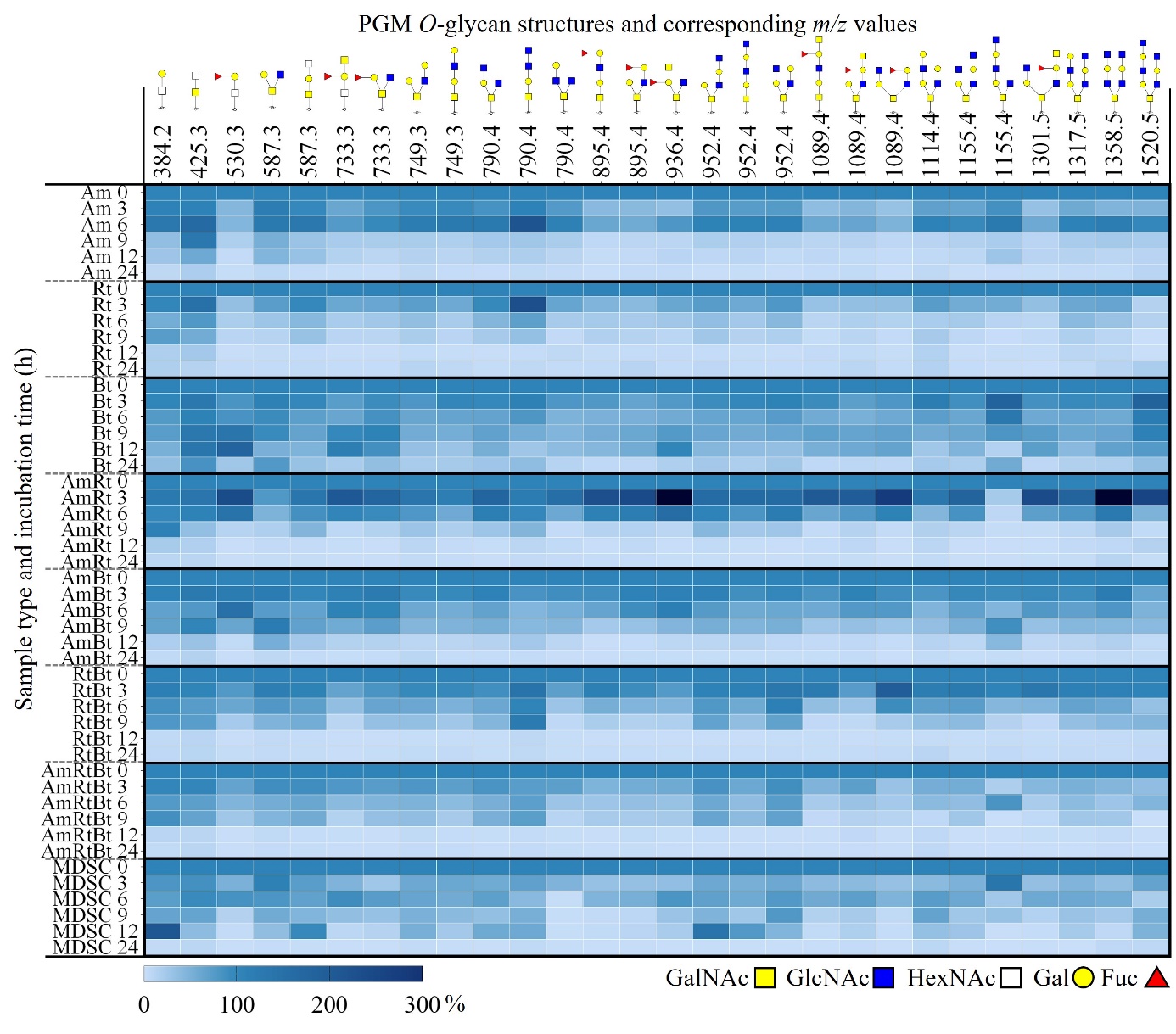


Figure S9. Heatmap of the foremost abundant mucin *O-*glycans resulting from replicate R1 PGM incubation with *A. muciniphila* (Am), *R. torques* (Rt), *B. thetaiotaomicron* (Bt), co-cultures (AmRt, AmBt, RtBt, AmRtBt), and the mucin-degrading synthetic community (MDSC), demonstrating the degradation rates of *O-*glycans during 24 h incubation. The intensity values (obtained from the peak areas) of the *O-*glycans at 0 h were set to 100% and the intensity values of 3 – 24 h are shown relative to the values at 0 h. The left y-axis indicates the bacterial cultures and the sampling time (h). The top x-axis displays the *m/z* values of the corresponding [M-H]^-^ *O-*glycan structures. *O-*glycan structures are based on acquired fragmentation data (PGC-LC-MS/MS). Sialylated *O-*glycan structures are not shown due to their low abundance and complete degradation within 3 - 6 h by each culture.


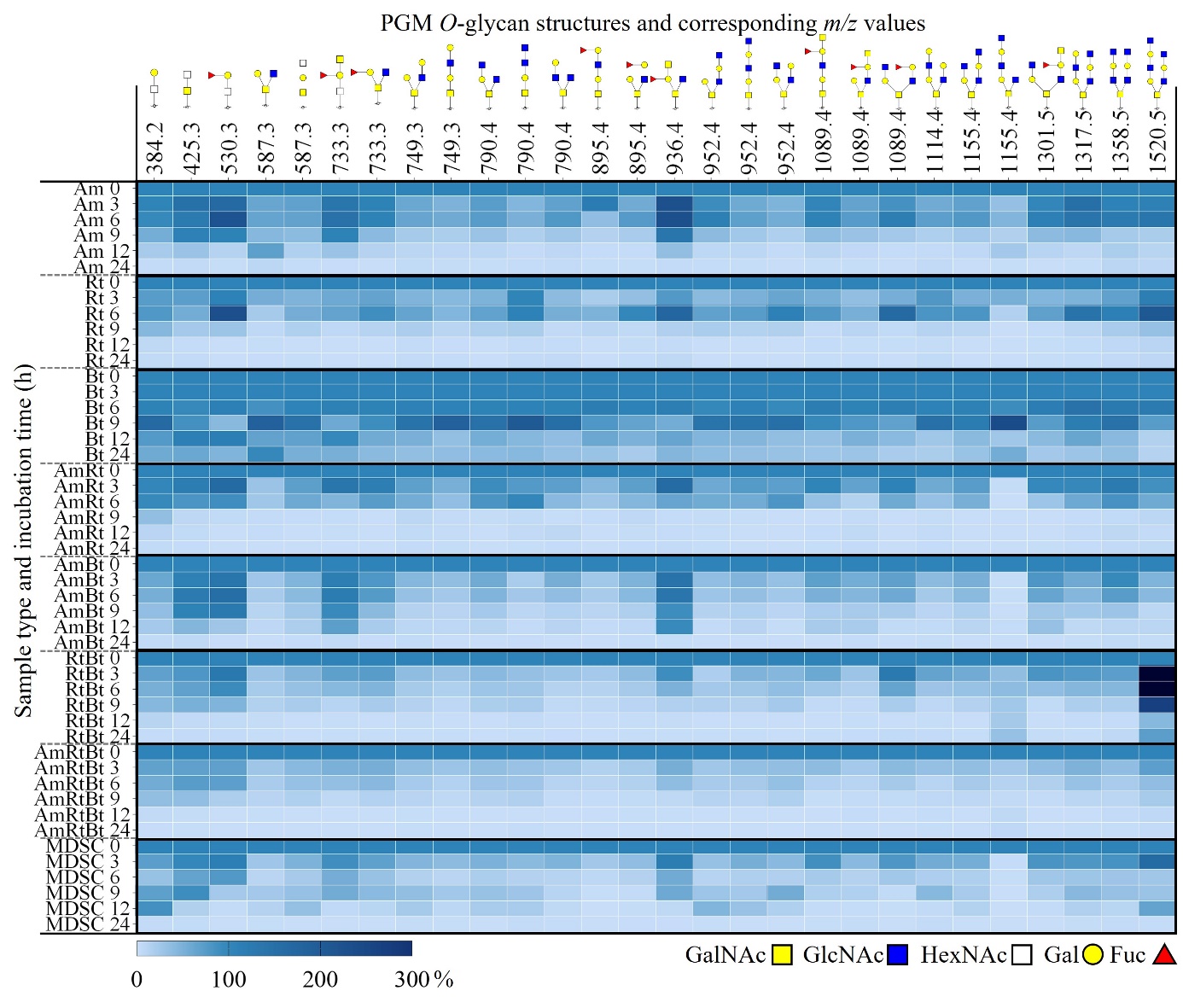


Figure S10. Heatmap of the foremost abundant mucin *O-*glycans resulting from replicate R2 PGM incubation with *A. muciniphila* (Am), *R. torques* (Rt), *B. thetaiotaomicron* (Bt), co-cultures (AmRt, AmBt, RtBt, AmRtBt), and the mucin-degrading synthetic community (MDSC), demonstrating the degradation rates of *O-*glycans during 24 h incubation. The intensity values (obtained from the peak areas) of the *O-*glycans at 0 h were set to 100% and the intensity values of 3 – 24 h are shown relative to the values at 0 h. The left y-axis indicates the bacterial cultures and the sampling time (h). The top x-axis displays the *m/z* values of the corresponding [M-H]^-^ *O-*glycan structures. *O-*glycan structures are based on acquired fragmentation data (PGC-LC-MS/MS). Sialylated *O-*glycan structures are not shown due to their low abundance and complete degradation within 3 - 6 h by each culture.


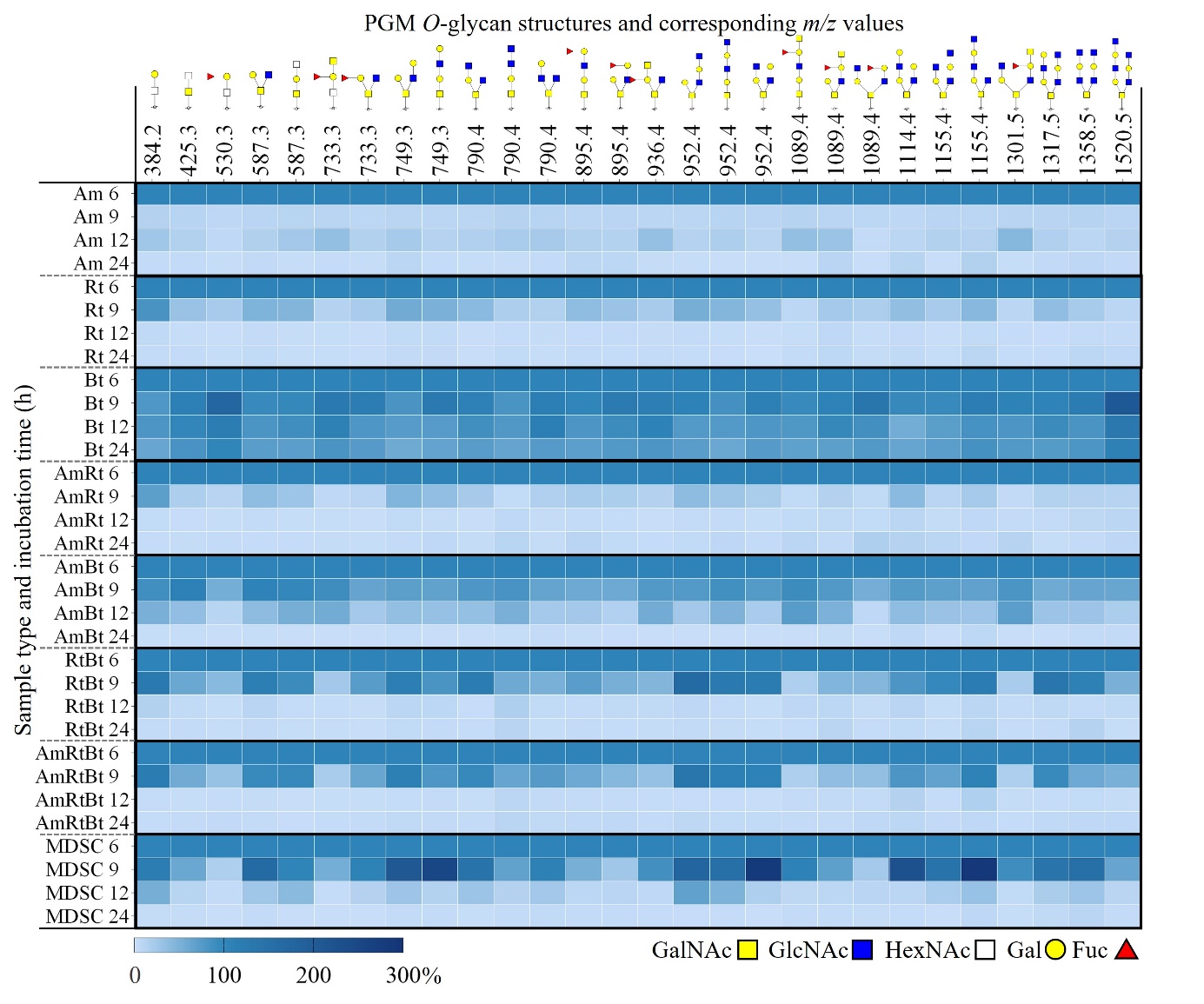


Figure S11. Heatmap of the foremost abundant mucin *O-*glycans resulting from replicate R3 PGM incubation with *A. muciniphila* (Am), *R. torques* (Rt), *B. thetaiotaomicron* (Bt), co-cultures (AmRt, AmBt, RtBt, AmRtBt), and the mucin-degrading synthetic community (MDSC), demonstrating the degradation rates of *O-*glycans during 6 - 24 h incubation (insufficient sample was available from 0 h and 3 h). The intensity values (obtained from the peak areas) of the *O-*glycans at 6 h were set to 100% and the intensity values of 9 – 24 h are shown relative to the values at 0 h. The left y-axis indicates the bacterial cultures and the sampling time (h). The top x-axis displays the *m/z* values of the corresponding [M-H]^-^ *O-*glycan structures. *O-*glycan structures are based on acquired fragmentation data (PGC-LC-MS/MS). Sialylated *O-*glycan structures are not shown due to their low abundance and complete degradation within 6 h by each culture as shown.


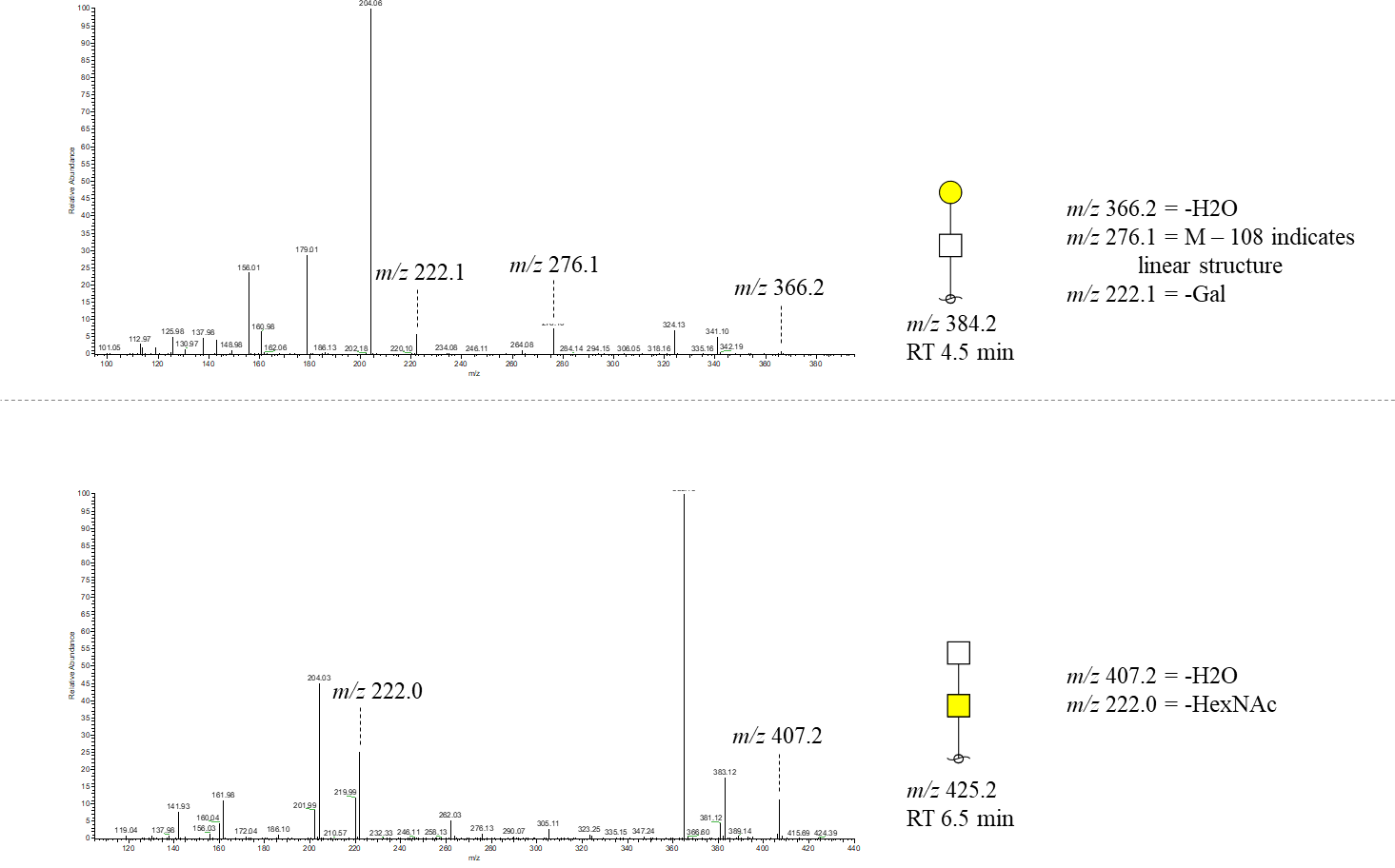


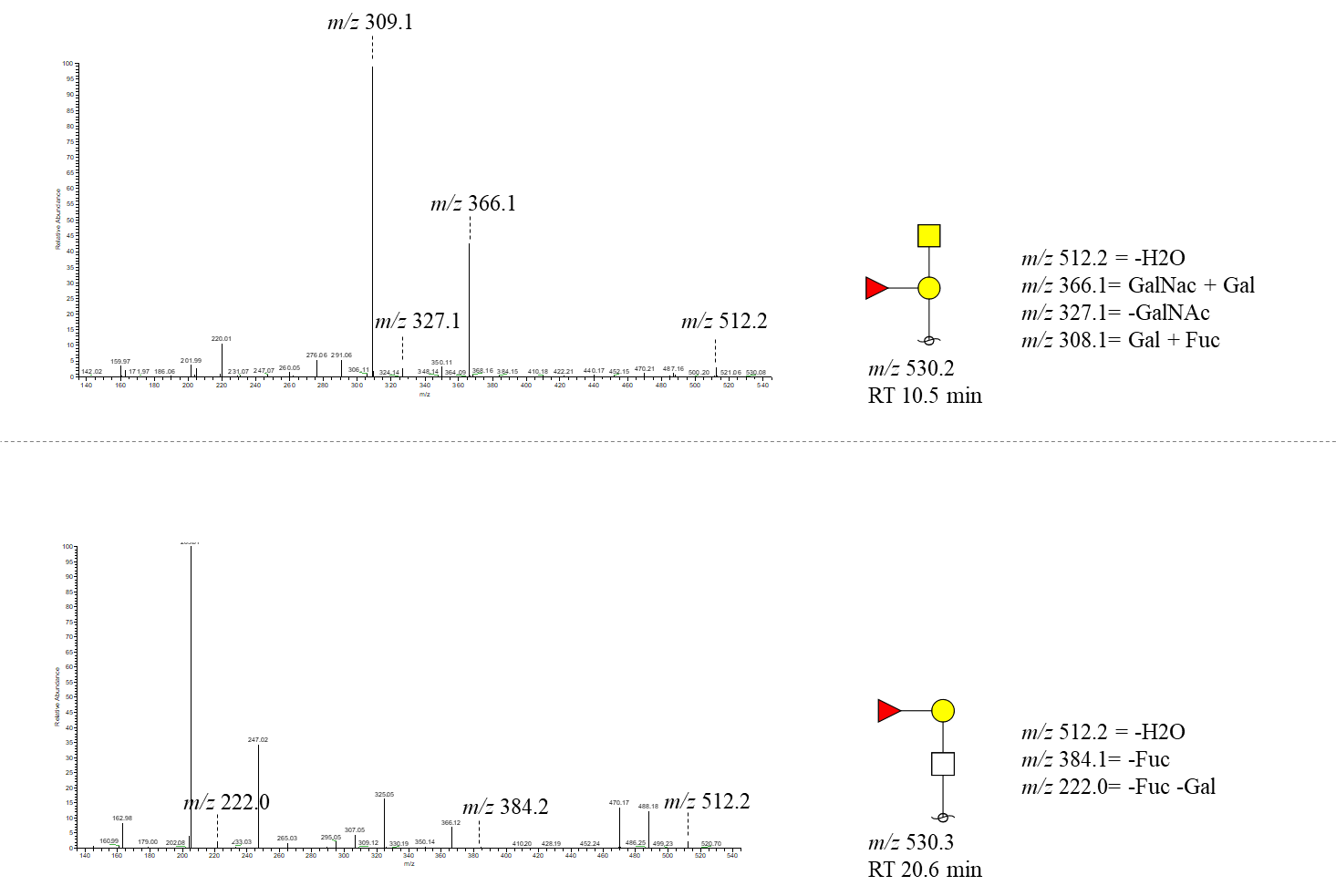


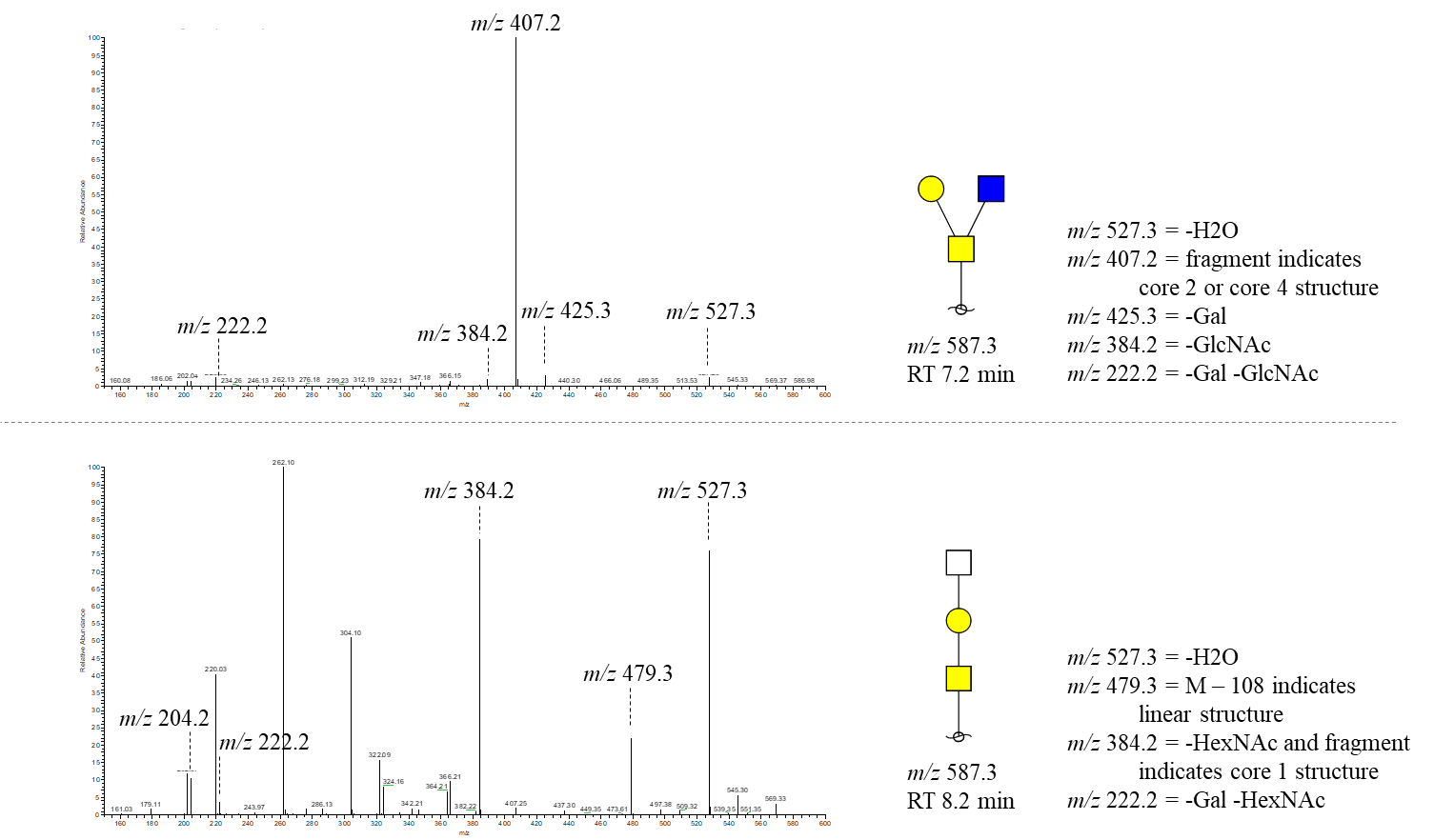


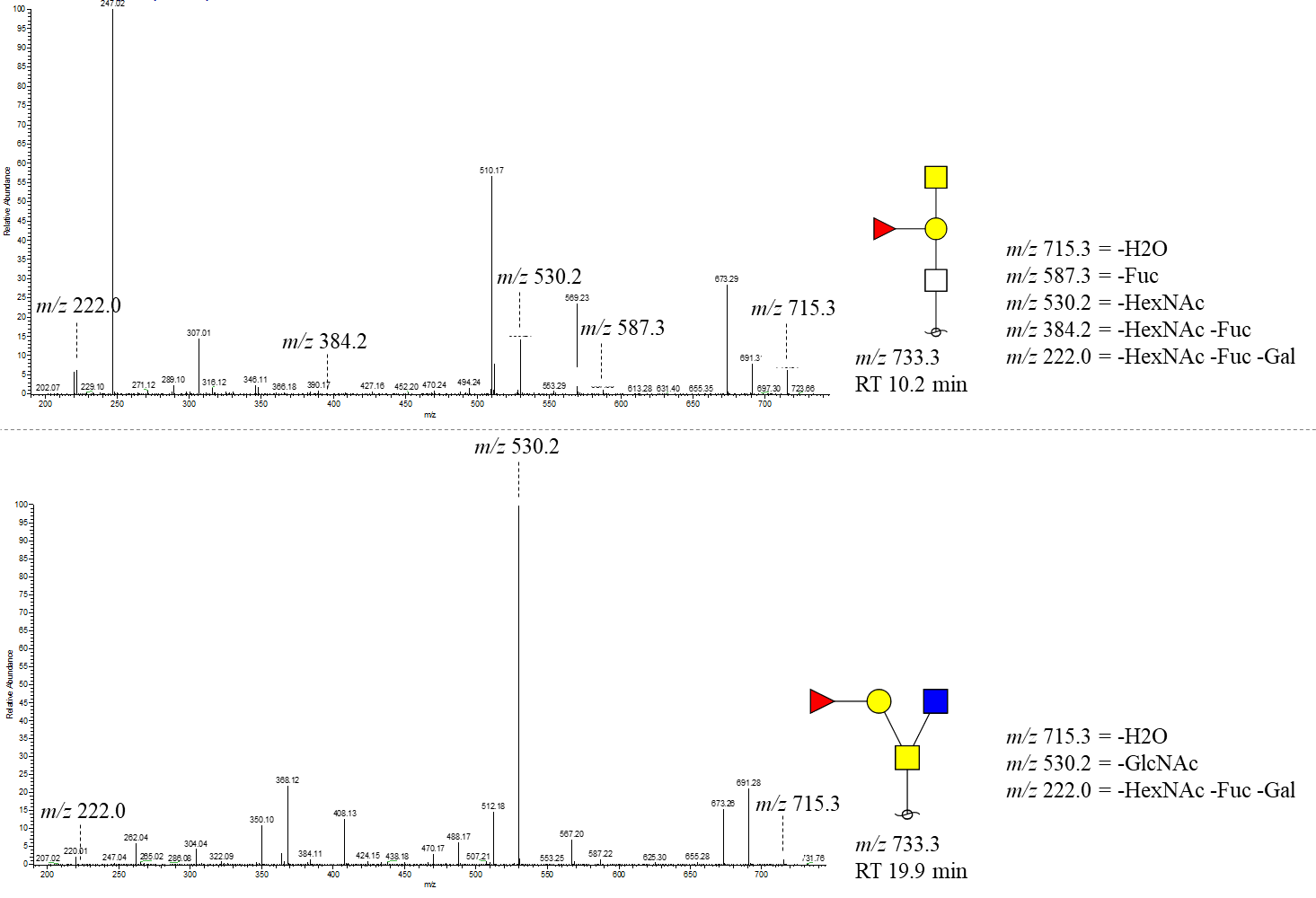


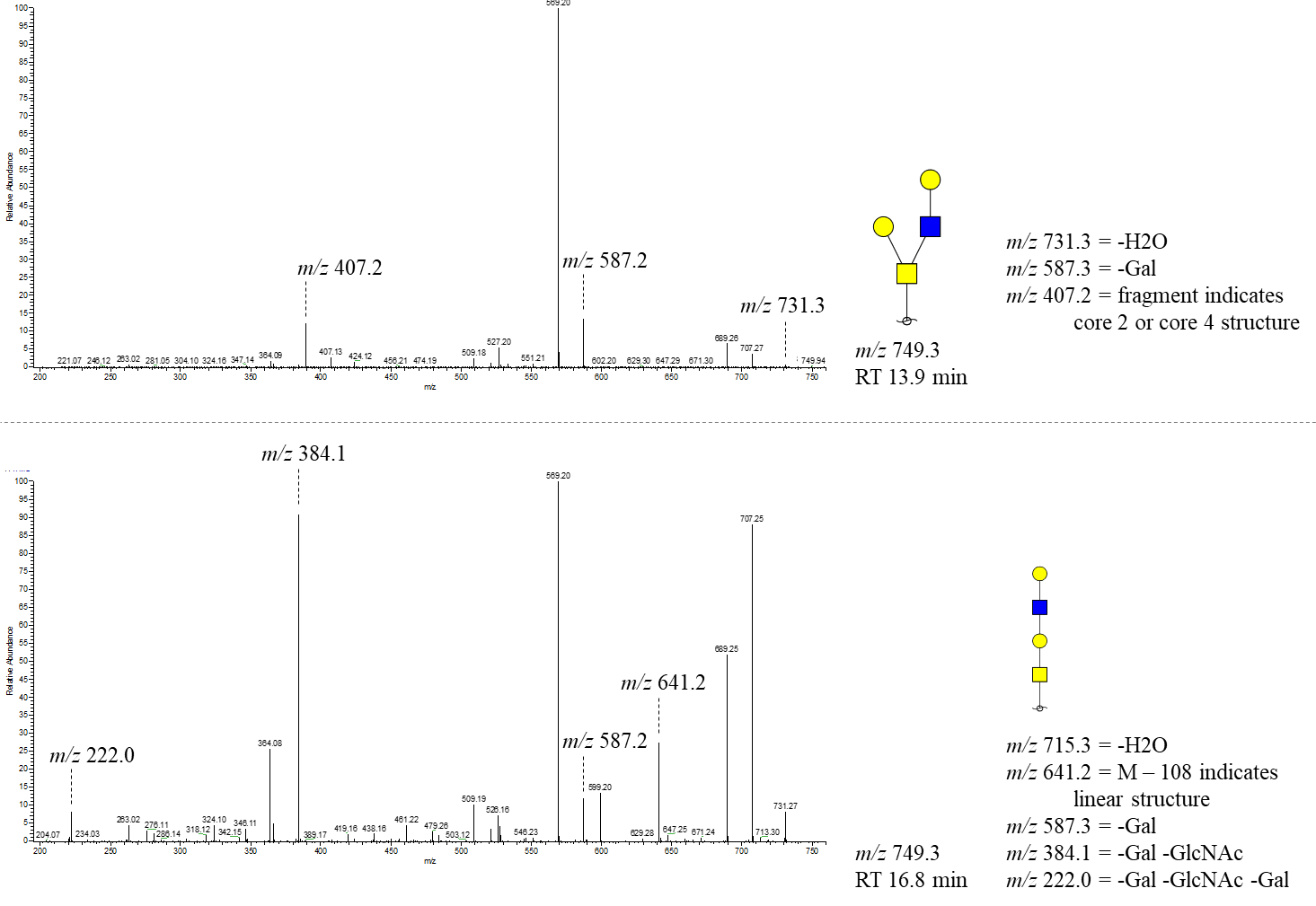


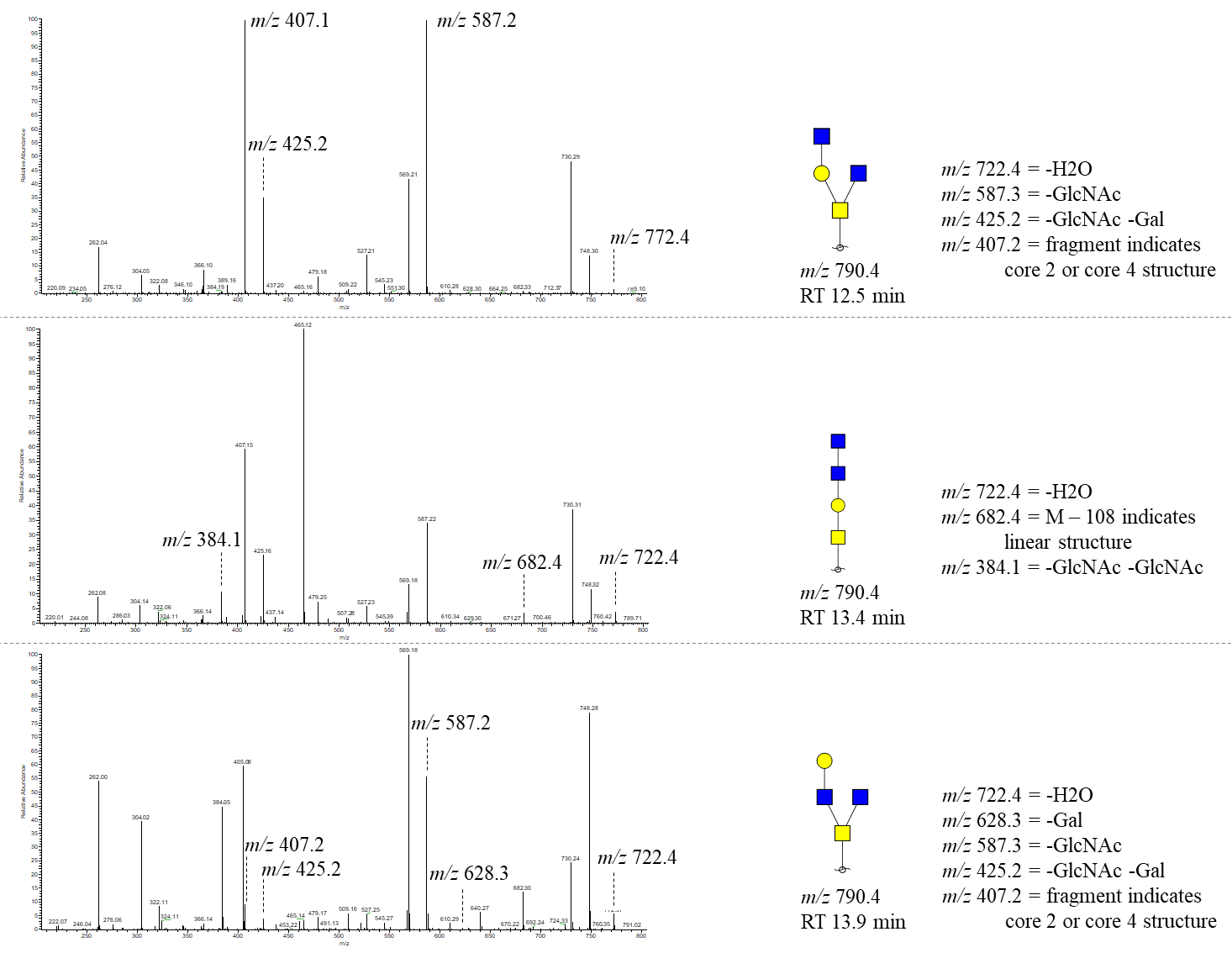


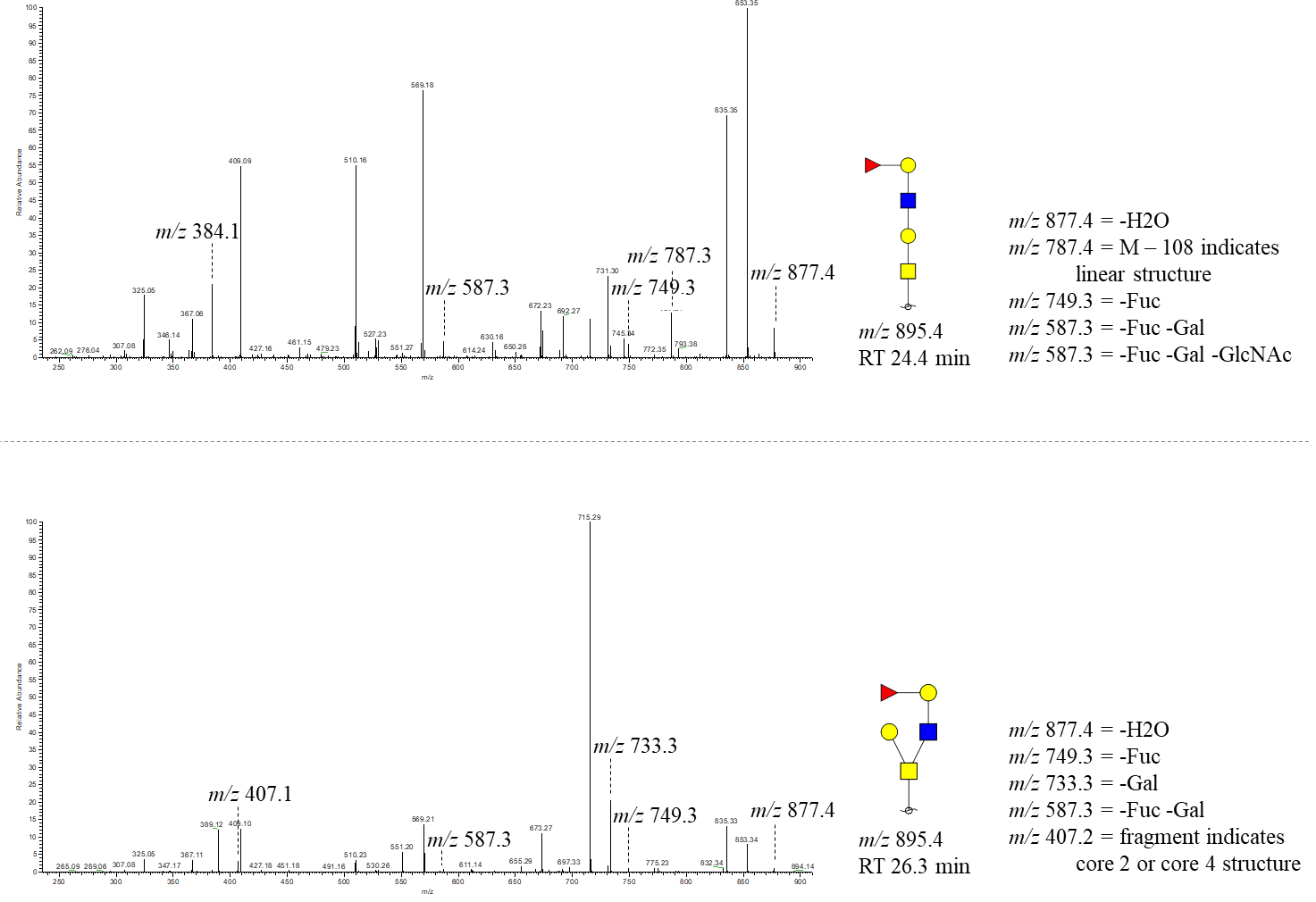


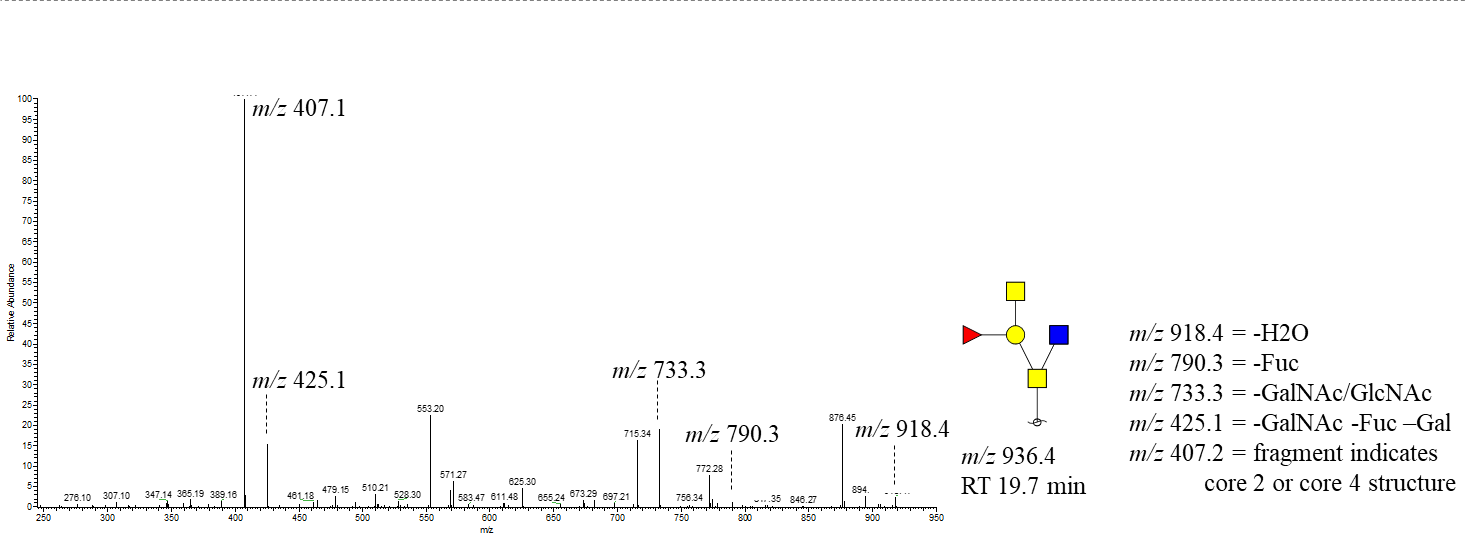


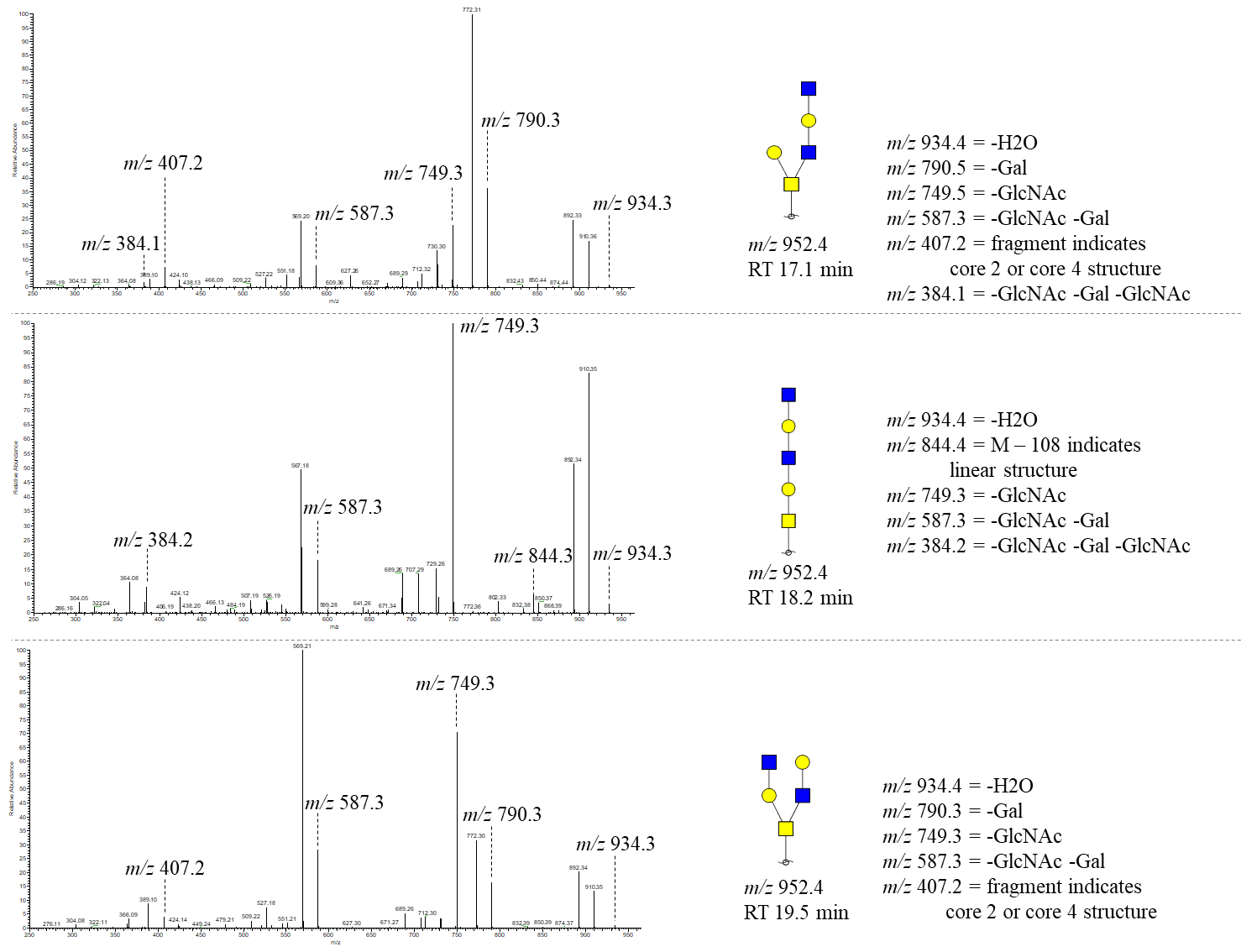


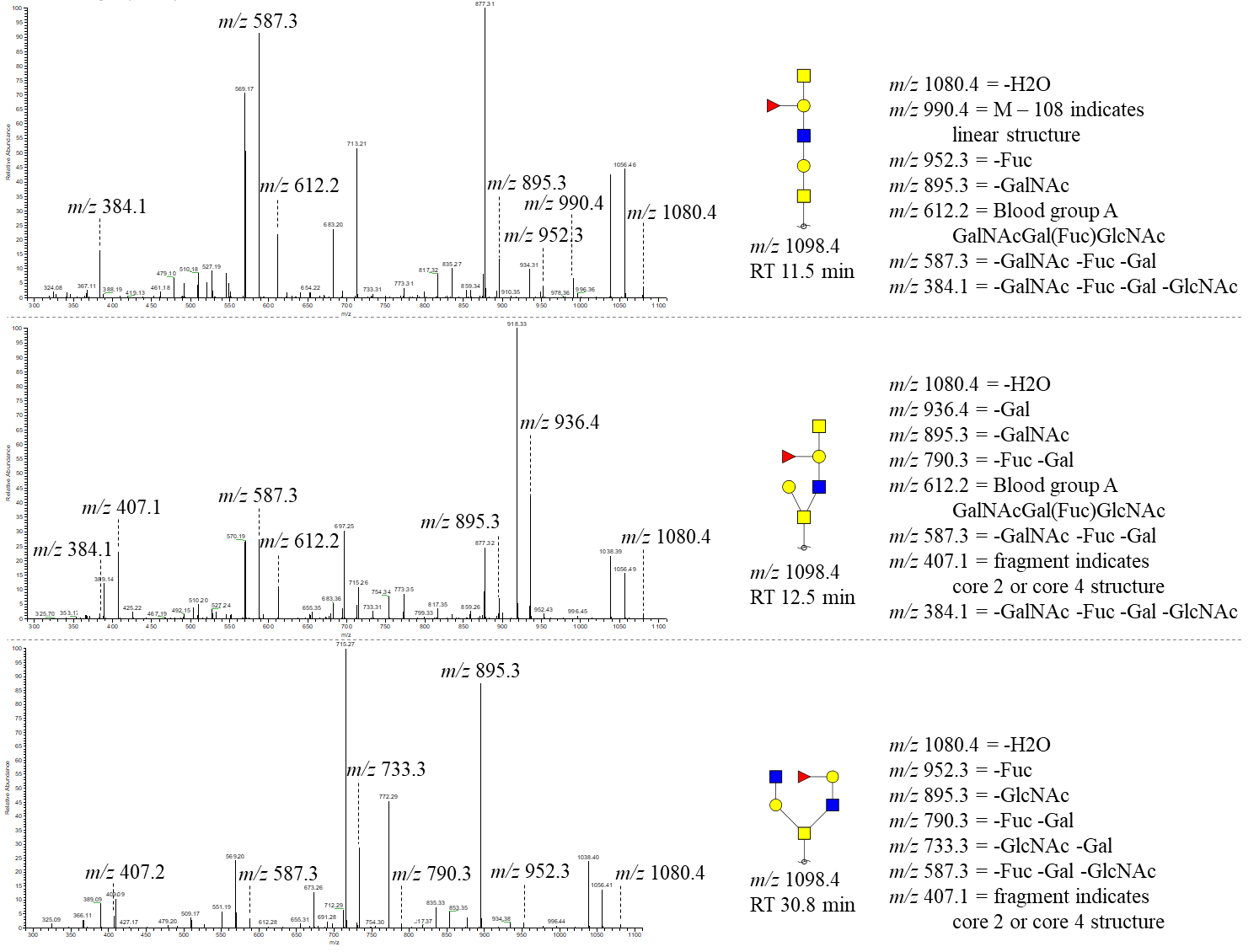


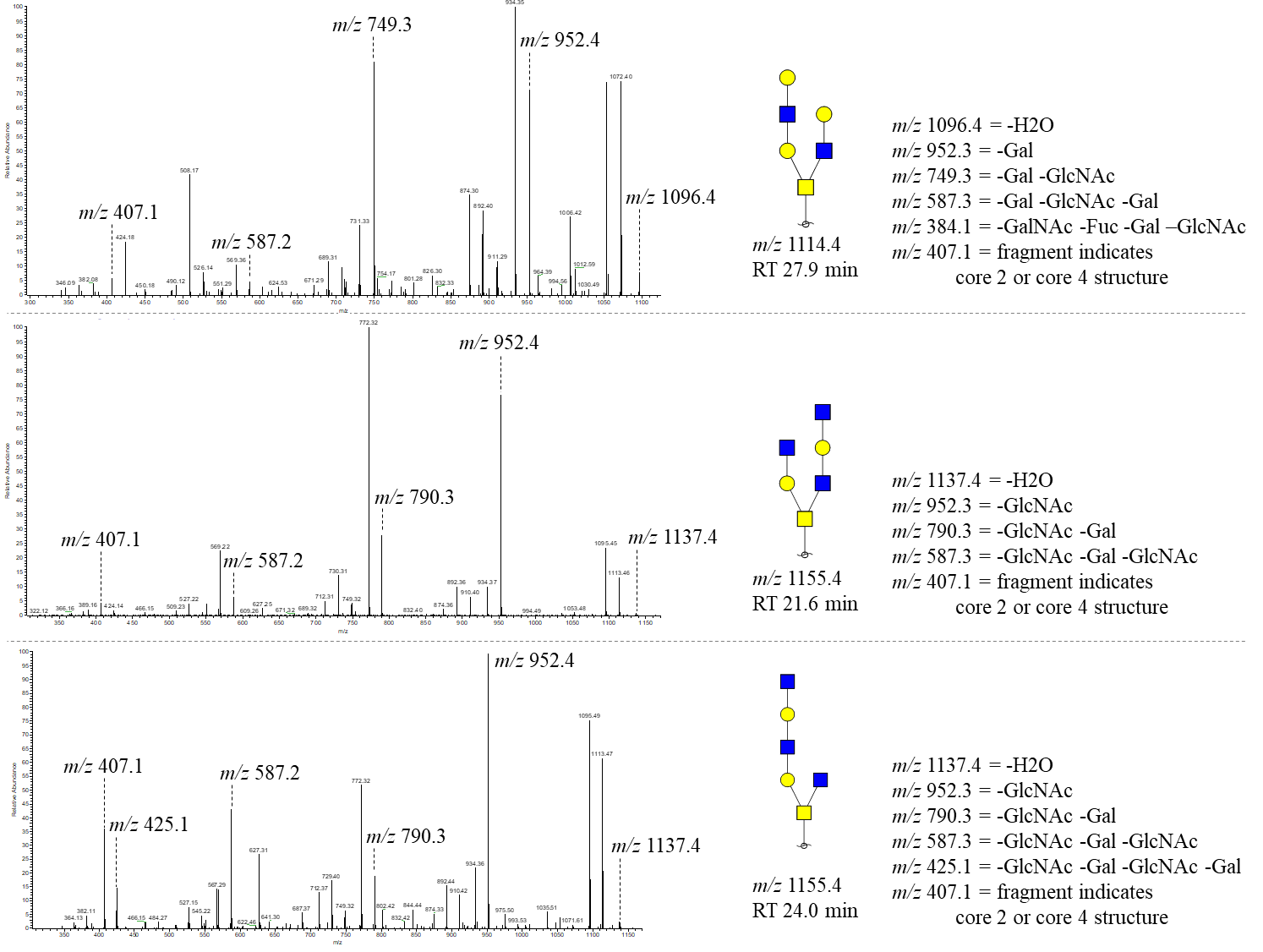


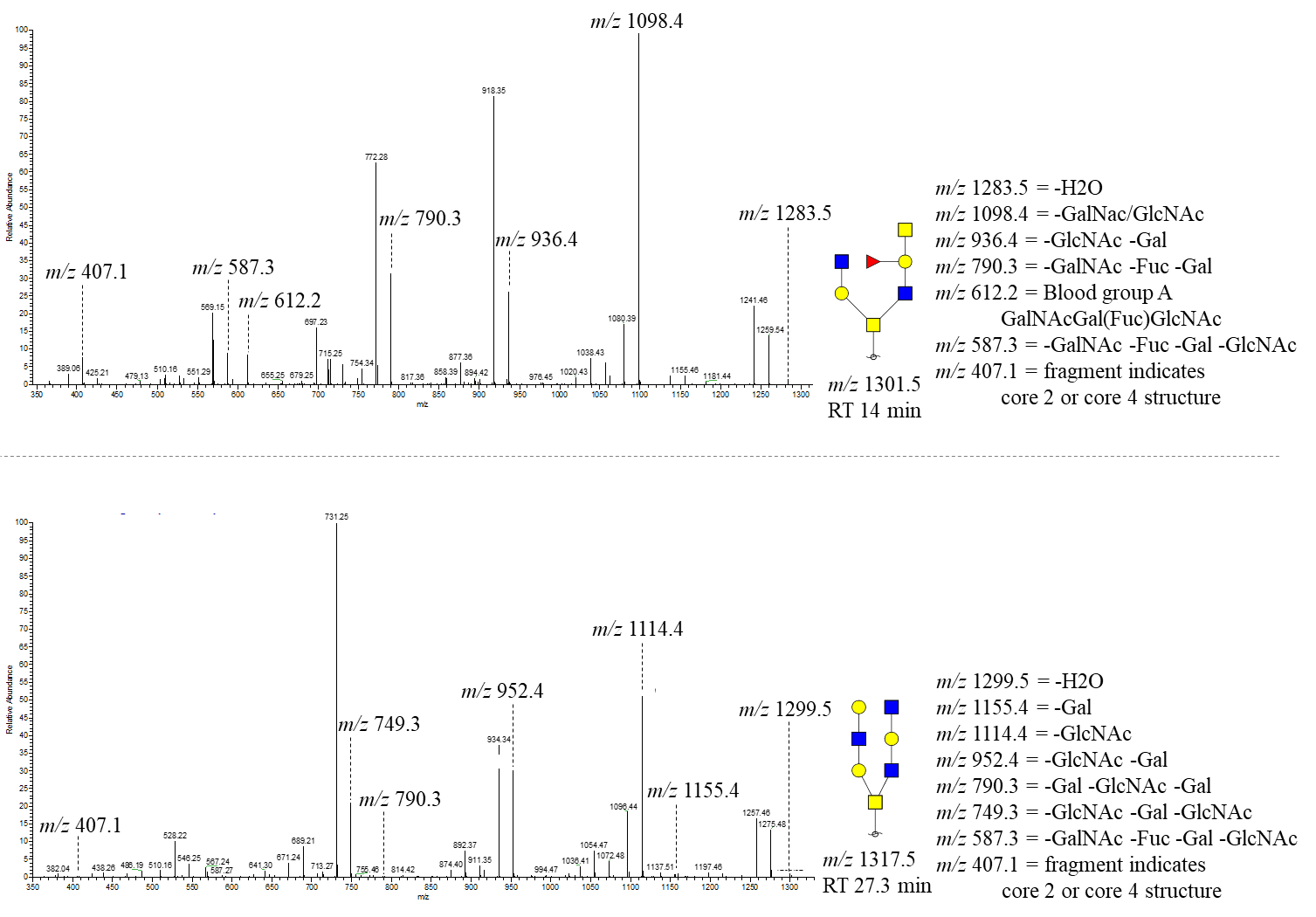


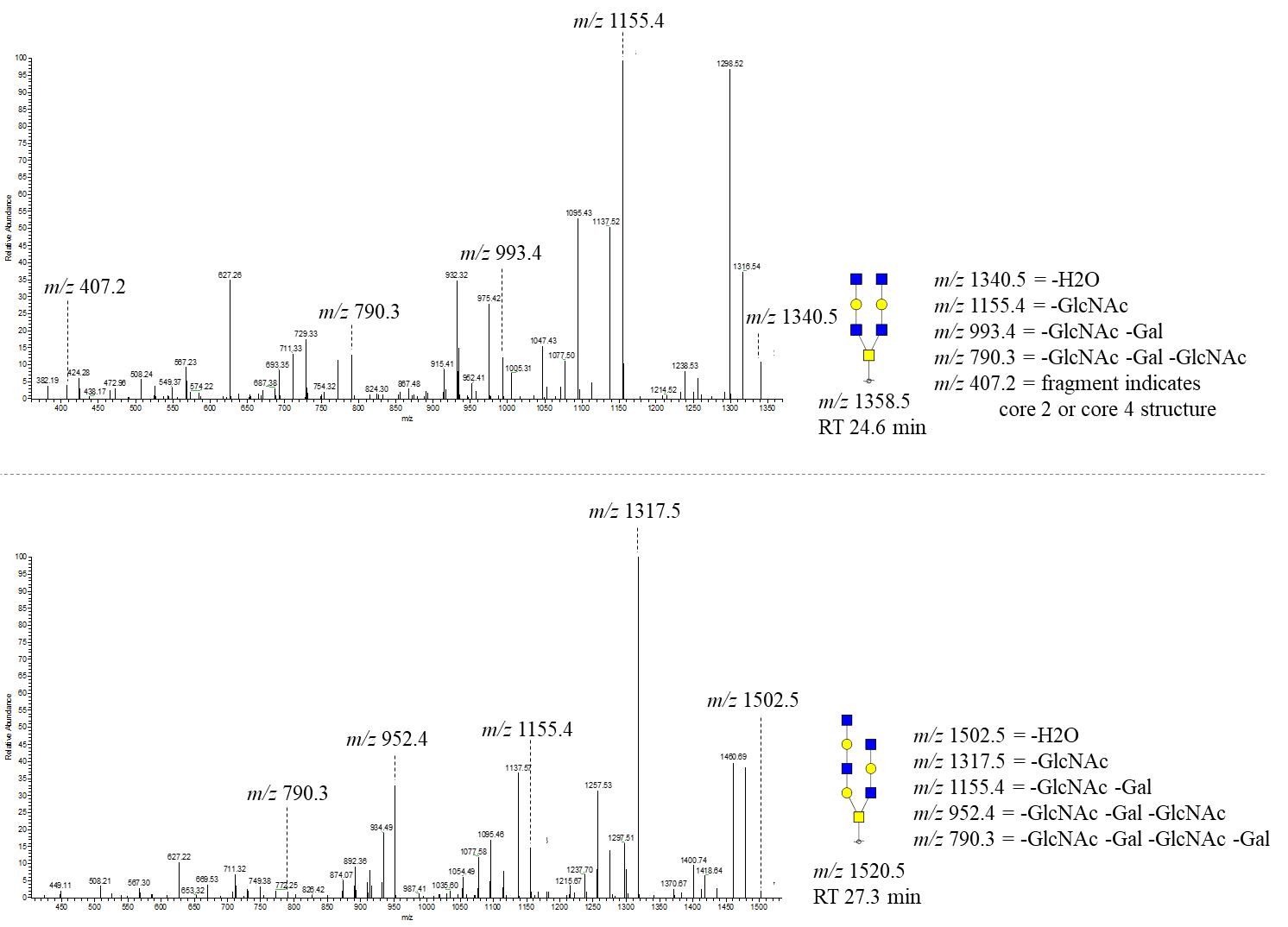


Figure S12. PGC-LC-MS/MS fragmentation spectra demonstrating the identification of the present *O-*glycans remaining on the protein backbone after incubation of PGM with co-culture monocultures *A. muciniphila*, *R. torques*, *B. thetaiotaomicron*, co-cultures thereof, and the MDSC during 24 h. *O-*glycans were released prior to analysis.


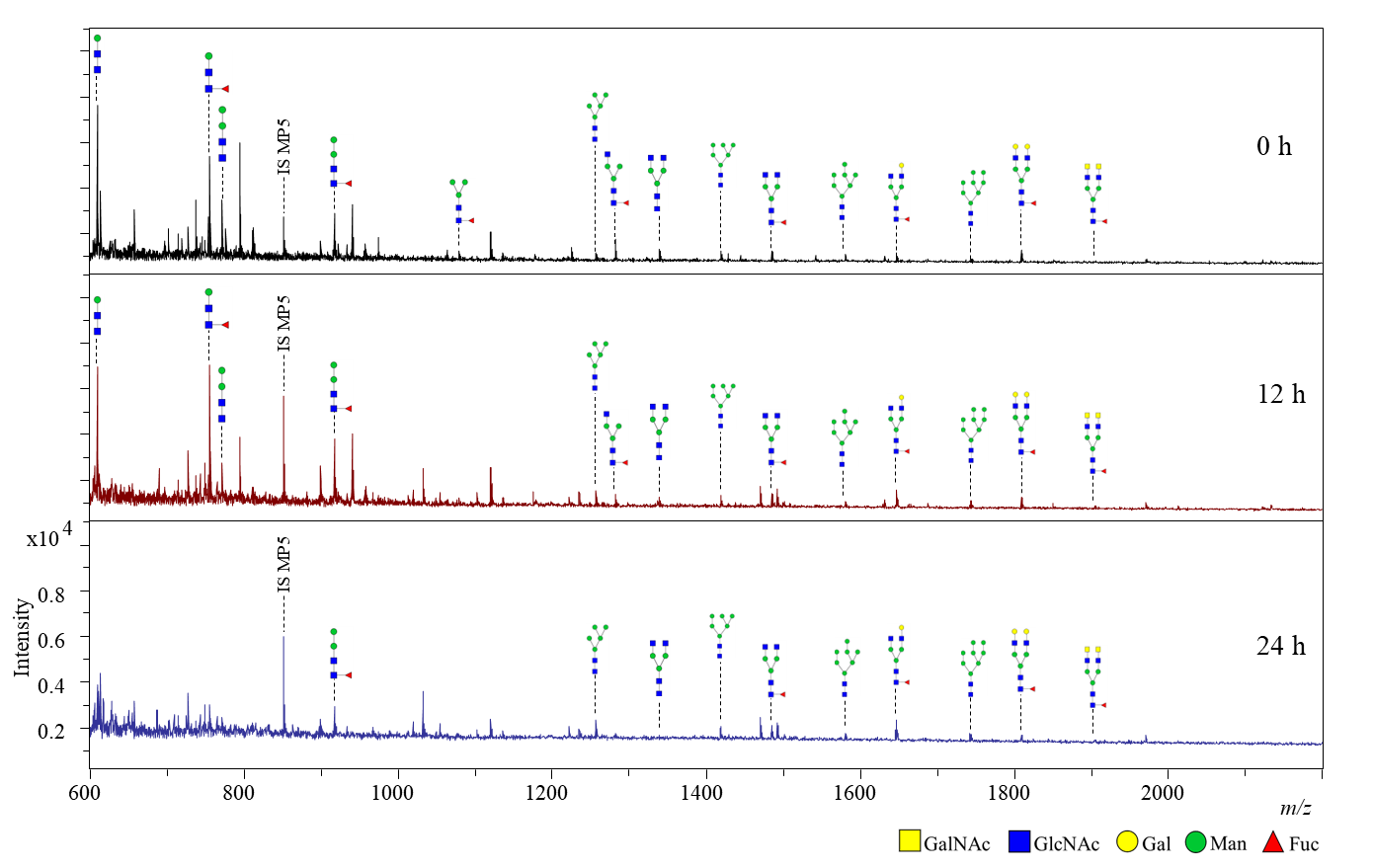


Figure S13. MALDI-TOF mass spectra demonstrating the *N-*glycan patterns remaining on the protein backbone after incubation of PGM with *A. muciniphila* during 24 h. *N-*glycans were enzymatically released prior to analysis. The x-axis displays the *m/z* values of the corresponding [M+Na]^+^ *N-*glycan structures. IS MP5: internal standard maltopentaose DP5.


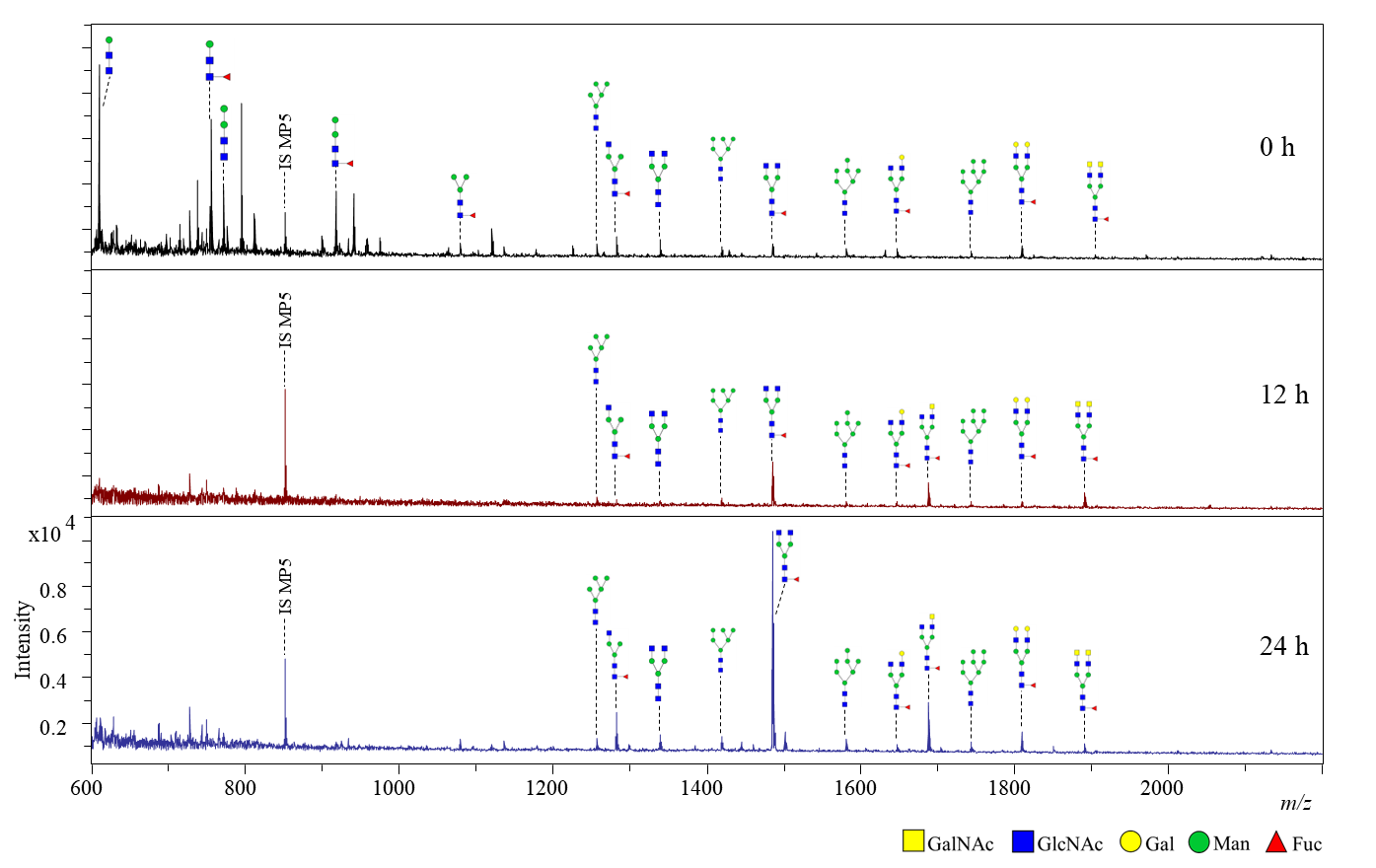


Figure S14. MALDI-TOF mass spectra demonstrating the *N-*glycan patterns remaining on the protein backbone after incubation of PGM with *R. torques* during 24 h. *N-*glycans were enzymatically released prior to analysis. The x-axis displays the *m/z* values of the corresponding [M+Na]^+^ *N-*glycan structures. IS MP5: internal standard maltopentaose DP5.


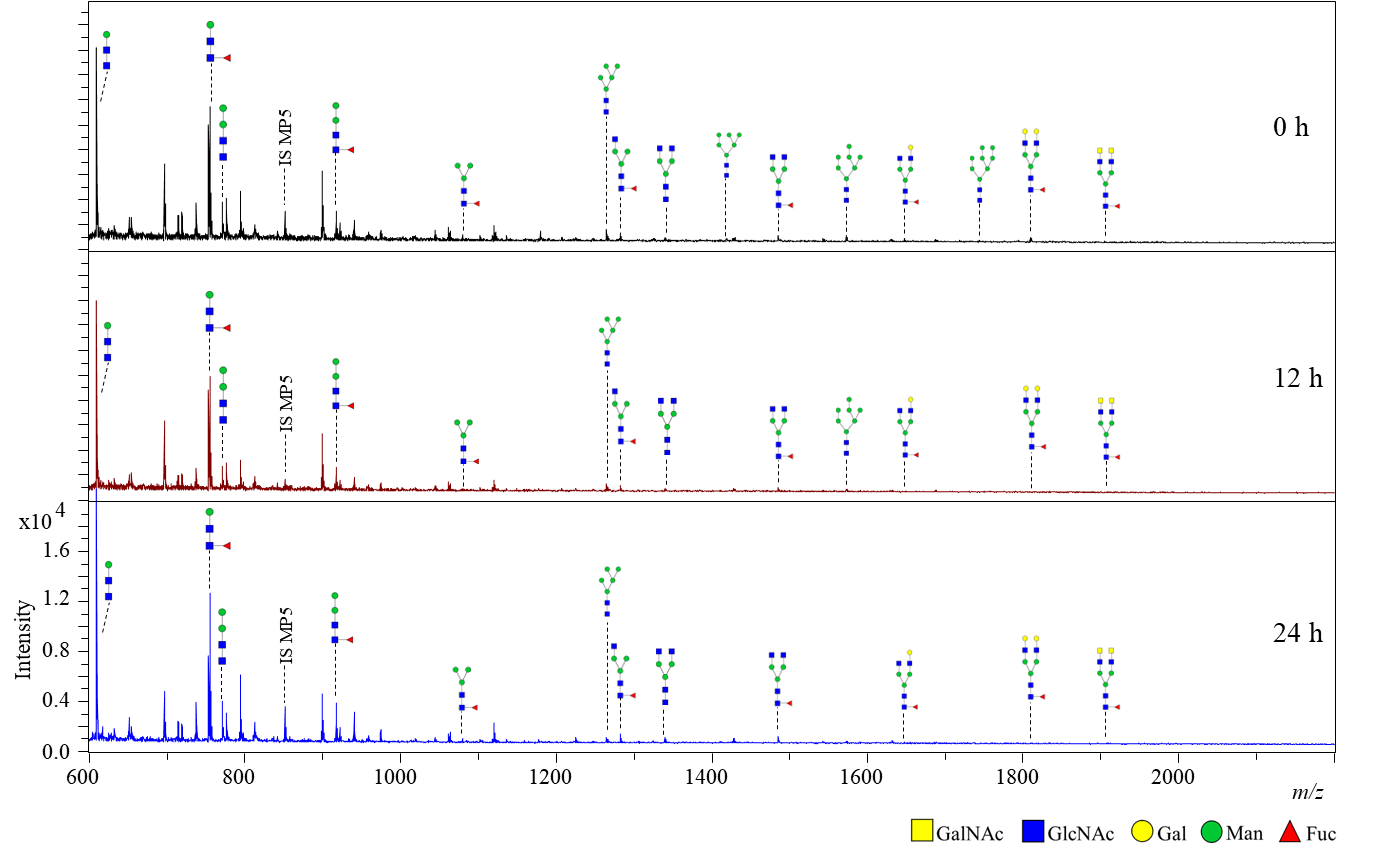


Figure S15. MALDI-TOF mass spectra demonstrating the *N-*glycan patterns remaining on the protein backbone after incubation of PGM with *B. thetaiotaomicron* during 24 h. *N-*glycans were enzymatically released prior to analysis. The x-axis displays the *m/z* values of the corresponding [M+Na]^+^ *N-*glycan structures. IS MP5: internal standard maltopentaose DP5.


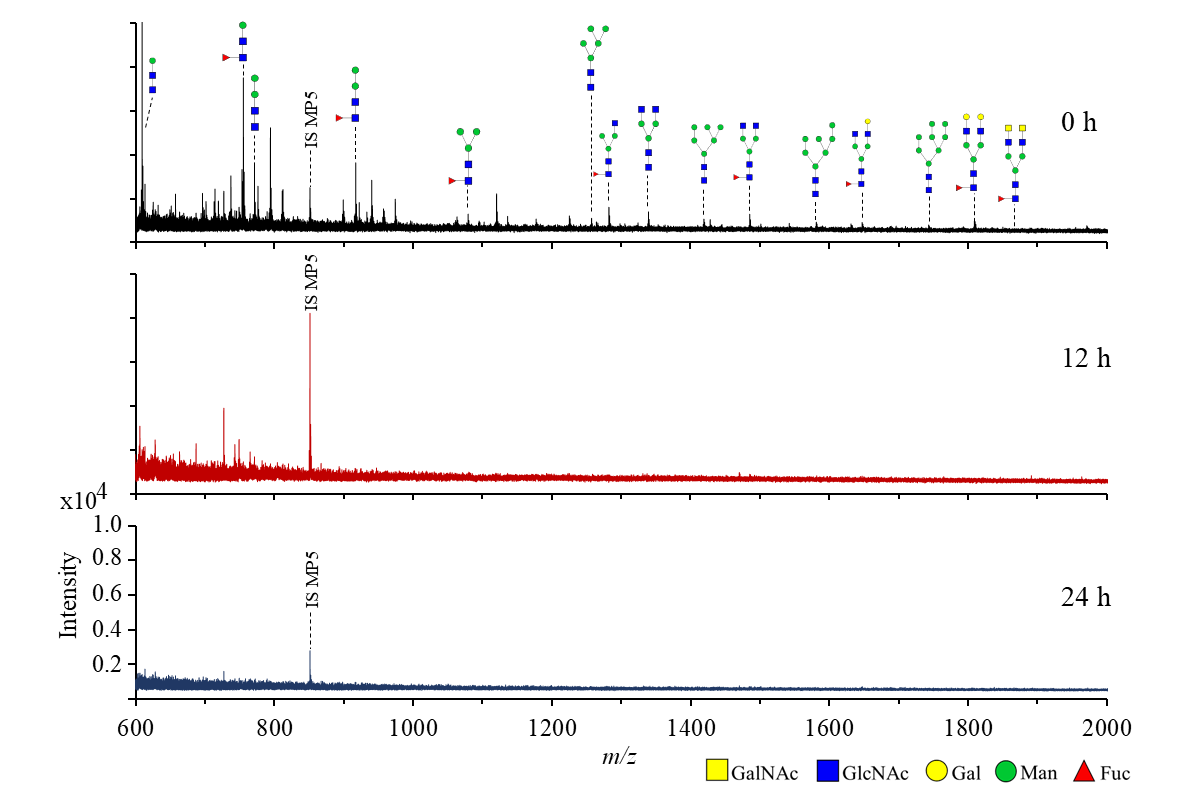


Figure S16. MALDI-TOF mass spectra demonstrating the *N-*glycan patterns remaining on the protein backbone after incubation of PGM with the MDSC during 24 h. *N-*glycans were enzymatically released prior to analysis. The x-axis displays the *m/z* values of the corresponding [M+Na]^+^ *N-*glycan structures. IS MP5: internal standard maltopentaose DP5.


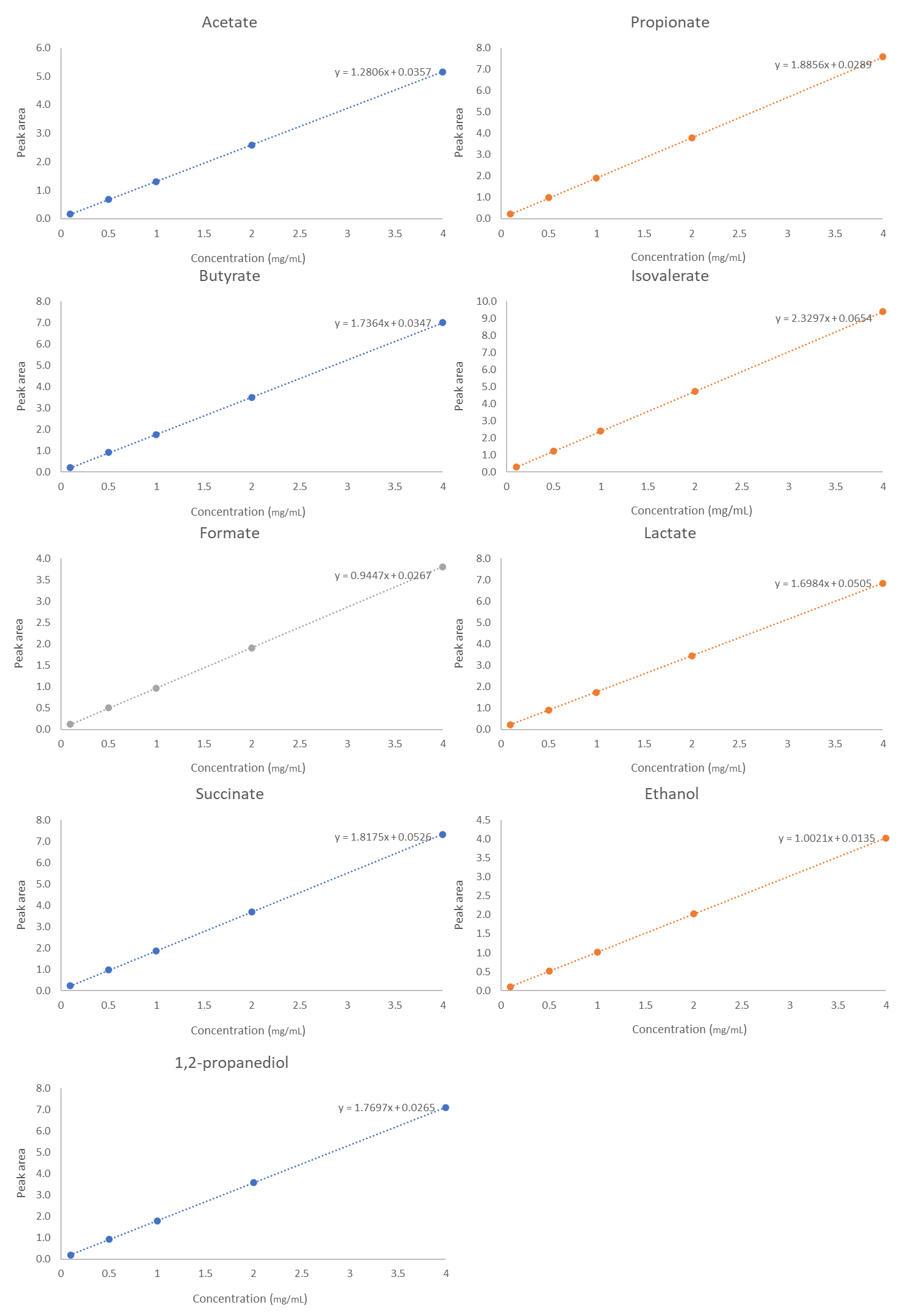


Figure S17. Calibration curves used for quantification of metabolites produced by the bacteria (Am, Rt, Bt, co-cultures, and the MDSC) when grown on PGM over time (0 – 24 h).


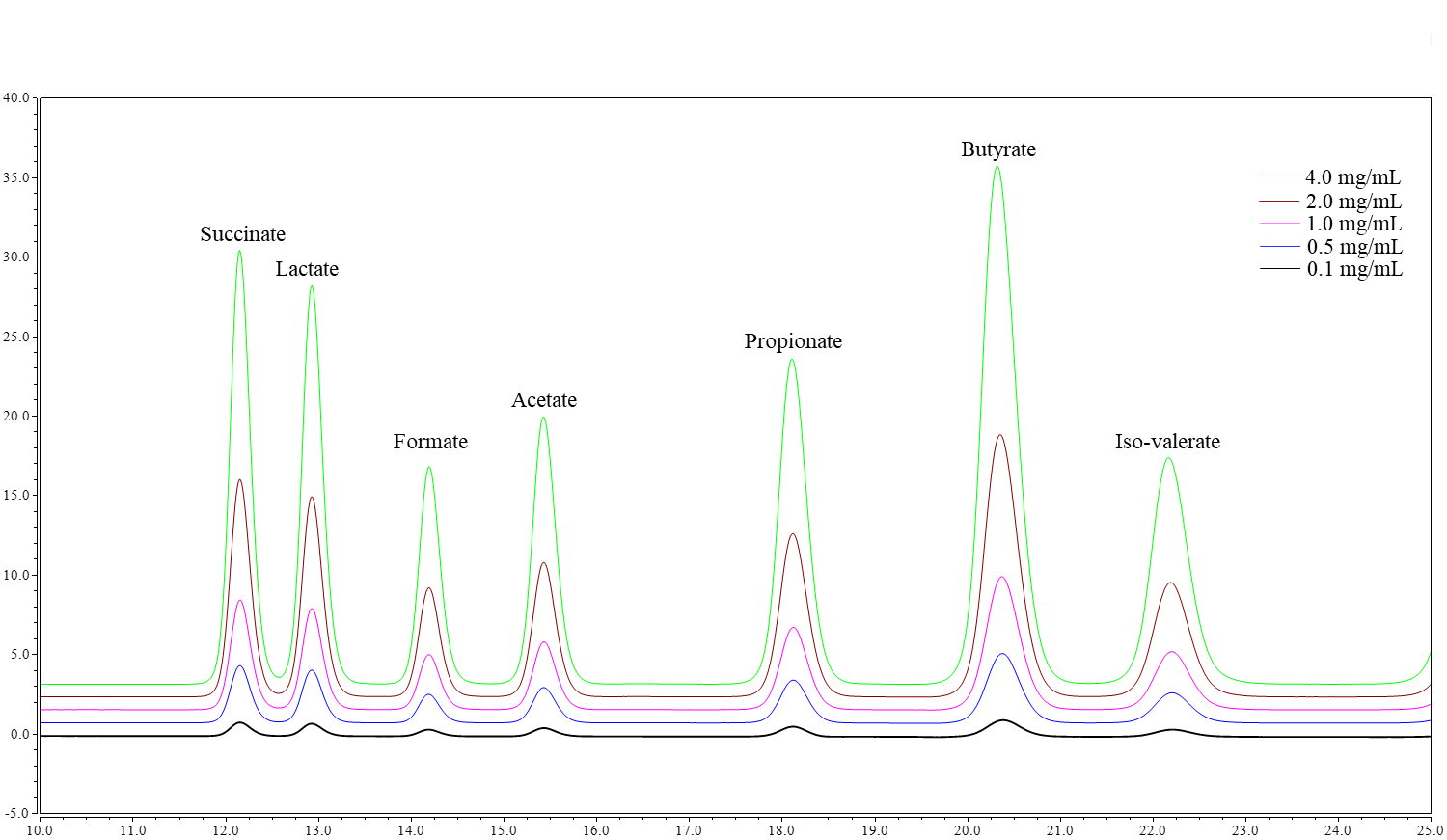


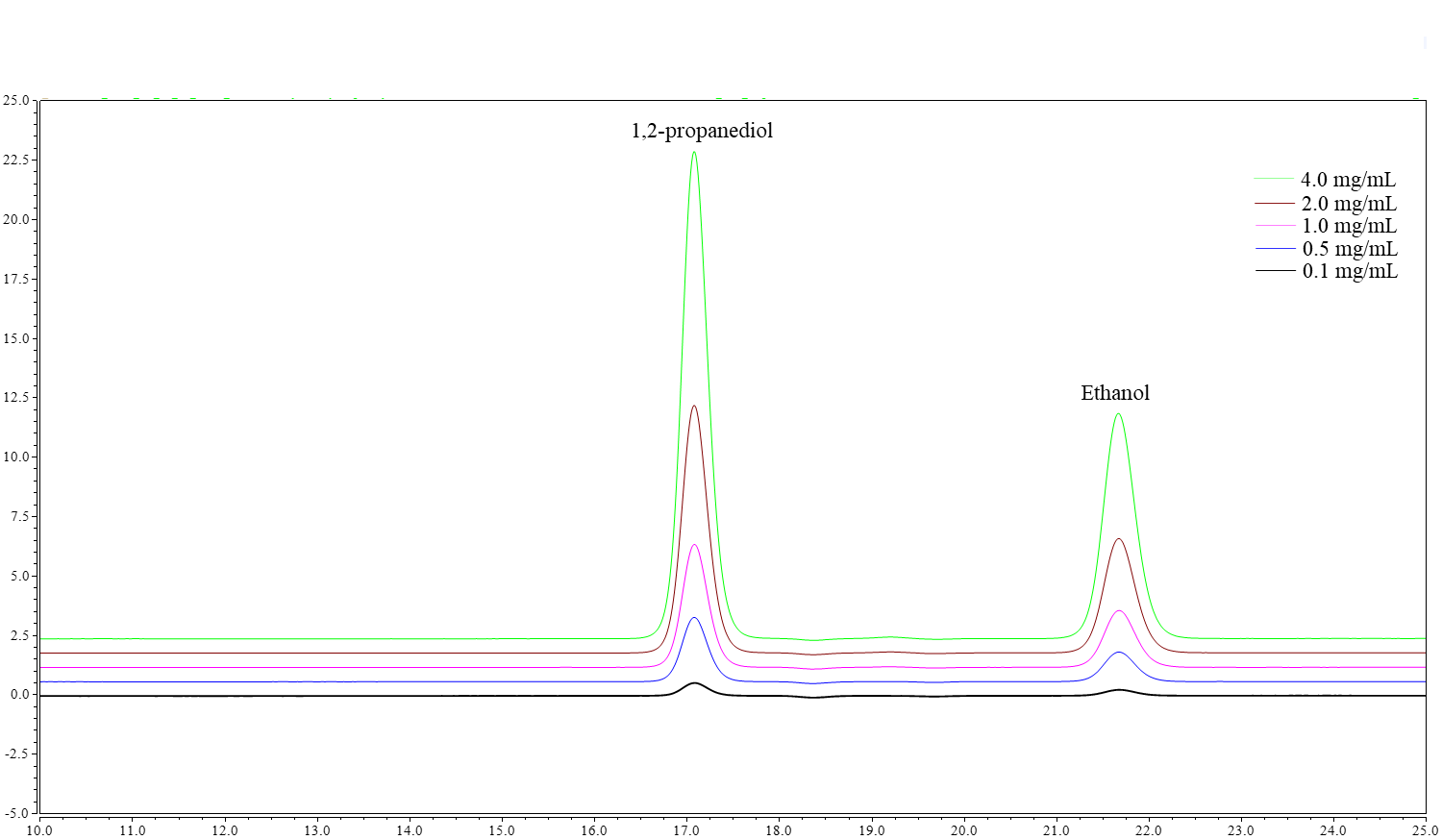


Figure S18. Standards used for calibration measured using HPLC. Two standards were used as there was overlap in the peaks originating from butyrate and ethanol. Therefore, 1 standard contained succinate, lactate, formate, acetate, propionate, butyrate, and iso-valerate (0.1 – 4.0 mg/mL; top figure) and the other standard contained 1,2-propanediol and ethanol (0.1 – 4.0 mg/mL; bottom figure). These standards were used for quantification of metabolites produced by the bacteria (Am, Rt, Bt, co-cultures, and the MDSC) when grown on PGM over time (0 – 24 h).


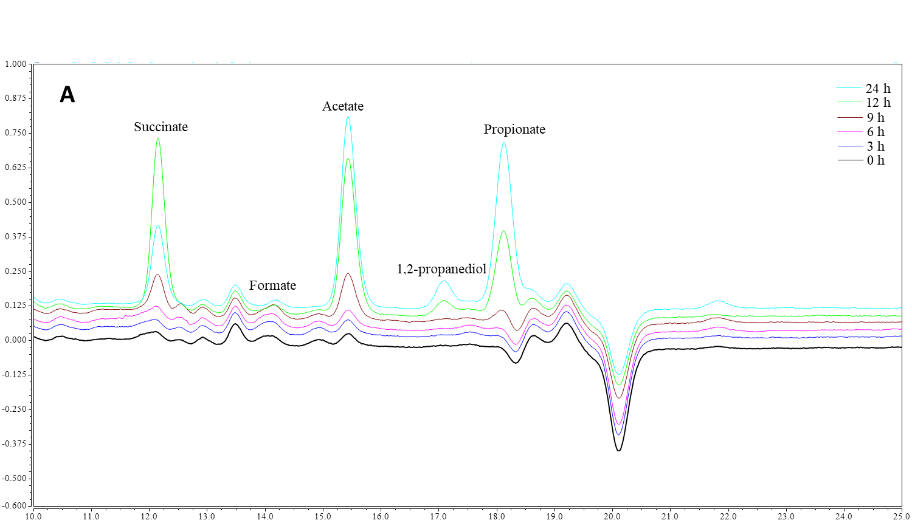


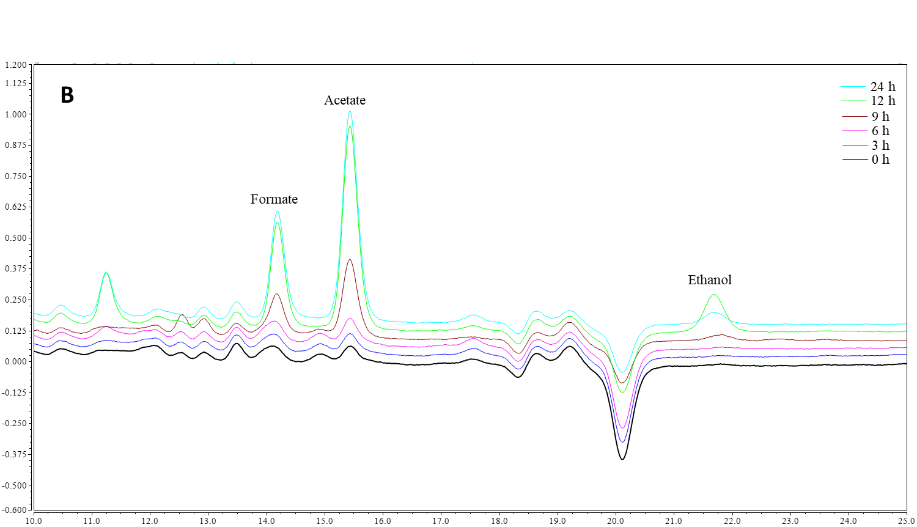


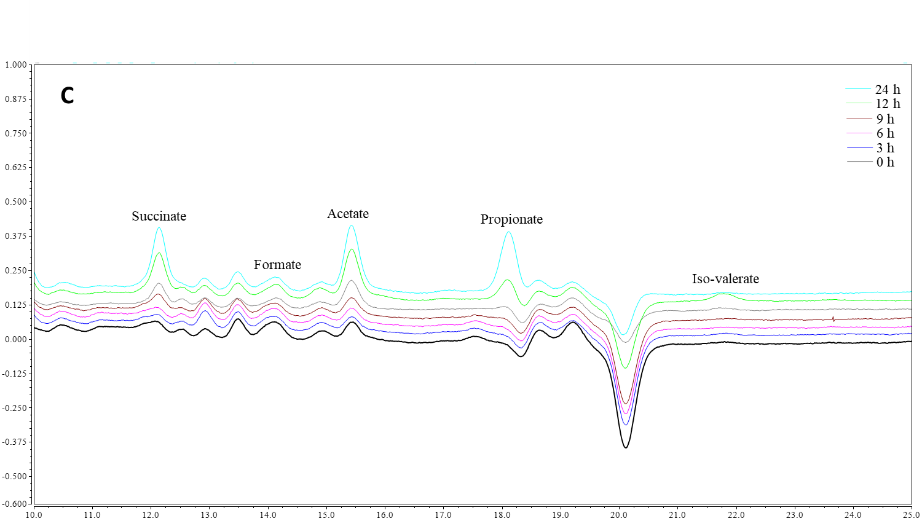


Figure S19. Example chromatograms of the metabolite production by (A) *A. muciniphila*, (B) *R. torques*, and (C) *B. thetaiotaomicron* grown on PGM over 24 h.

Table S1. Bactrial strains used and the associated number of 16S rRNA gene copies ^2^.

| **Strain** | **Number of 16S rRNA gene copies** |
| --- | --- |
| *Akkermansia muciniphila*DSM 22959*T* | 3 |
| *Bacteroides caccae*DSM 19024*T* | 5 |
| *Bacteroides fragilis*DSM 2151*T* | 6 |
| *Bacteroides thetaiotaomicron*DSM 2079*T* | 5 |
| *Phocaeicola vulgatus*ATCC 8482*T* | 7 |
| *Ruminococcus gnavus* ATCC 29149*T* | 5 |
| *Ruminococcus torques* ATCC 27756*T* | 10 |
| *Anaerostipes caccae*DSM 14662*T* | 4 |
| *Faecalibacterium duncaniae*DSM 17677*T* | 6 |
| *Anaerobutyricum hallii*DSM 3353*T* | 8 |
| *Agathobacter rectalis*ATCC 33656*T* | 5 |
| *Roseburia intestinalis*DSM 14610*T* | 6 |
| *Blautia hydrogenotrophica*DSM 10507*T* | 5 |
| *Desulfovibrio piger*DSM 749*T* | 7 |
| *Methanobrevibacter smithii*DSM 11975 | 3 |
| *Bacillus thuringiensis*(contamination) | 14 |

**References**

1. Shetty S.A., Kostopoulos I., Geerlings S.Y., Smidt H., de Vos W.M., Belzer C. Dynamic metabolic interactions and trophic roles of human gut microbes identified using a minimal microbiome exhibiting ecological properties. *ISME Journal*. **2022**; 16(9), 2144-2159. doi:10.1038/s41396-022-01255-2

2. Berkhout M.D., Ioannou A., de Ram C., Boeren S., Plugge C.M., Belzer C. Mucin-driven ecological interactions in an *in vitro* synthetic community of human gut microbes. *Glycobiology*. **2024**; 34(12), 1. doi:10.1093/glycob/cwae085
